# Cardiac Alterations in Human African Trypanosomiasis (*T.b. gambiense*) with Respect to the Disease Stage and Antiparasitic Treatment

**DOI:** 10.1371/journal.pntd.0000383

**Published:** 2009-02-17

**Authors:** Johannes A. Blum, Caecilia Schmid, Christian Burri, Christoph Hatz, Carol Olson, Blaise Fungula, Leon Kazumba, Patrick Mangoni, Florent Mbo, Kambau Deo, Alain Mpanya, Amadeo Dala, Jose R. Franco, Gabriele Pohlig, Michael J. Zellweger

**Affiliations:** 1 Swiss Tropical Institute, Basel, Switzerland; 2 Immtech Pharmaceuticals Inc., Vernon Hills, Illinois, United States of America; 3 Hôpital Evangélique de Vanga, Vanga, Democratic Republic of Congo; 4 Centre Neuro Psycho Pathologique, Kinshasa, Democratic Republic of Congo; 5 Hôpital General de Reference Bandundu, Bandundu, Democratic Republic of Congo; 6 Hôpital Evangélique de Kikongo, Kikongo, Democratic Republic of Congo; 7 CDTC Maluku, Maluku, Democratic Republic of Congo; 8 Instituto de Combate e de Controlo das Tripanossomíases, Luanda, Angola; 9 Malteser International, Malteser Hospital, Yei, Southern Sudan; 10 Cardiology Department, University Hospital, Basel, Switzerland; Institute of Tropical Medicine, Belgium

## Abstract

**Background:**

In Human African Trypanosomiasis, neurological symptoms dominate and cardiac involvement has been suggested. Because of increasing resistance to the available drugs for HAT, new compounds are desperately needed. Evaluation of cardiotoxicity is one parameter of drug safety, but without knowledge of the baseline heart involvement in HAT, cardiologic findings and drug-induced alterations will be difficult to interpret. The aims of the study were to assess the frequency and characteristics of electrocardiographic findings in the first stage of HAT, to compare these findings to those of second stage patients and healthy controls and to assess any potential effects of different therapeutic antiparasitic compounds with respect to ECG changes after treatment.

**Methods:**

Four hundred and six patients with first stage HAT were recruited in the Democratic Republic of Congo, Angola and Sudan between 2002 and 2007 in a series of clinical trials comparing the efficacy and safety of the experimental treatment DB289 to the standard first stage treatment, pentamidine. These ECGs were compared to the ECGs of healthy volunteers (n = 61) and to those of second stage HAT patients (n = 56).

**Results:**

In first and second stage HAT, a prolonged QTc interval, repolarization changes and low voltage were significantly more frequent than in healthy controls. Treatment in first stage was associated with repolarization changes in both the DB289 and the pentamidine group to a similar extent. The QTc interval did not change during treatment.

**Conclusions:**

Cardiac involvement in HAT, as demonstrated by ECG alterations, appears early in the evolution of the disease. The prolongation of the QTC interval comprises a risk of fatal arrhythmias if new drugs with an additional potential of QTC prolongation will be used. During treatment ECG abnormalities such as repolarization changes consistent with peri-myocarditis occur frequently and appear to be associated with the disease stage, but not with a specific drug.

## Author Summary

In Human African Trypanosomiasis (HAT), neurological symptoms dominate and cardiac involvement has been suggested. Because of increasing resistance to the available drugs for HAT, new compounds are desperately needed. Evaluation of cardiotoxicity is one parameter of drug safety, but without knowledge of the baseline heart involvement in HAT, cardiologic findings and drug-induced alterations will be difficult to interpret. The electrocardiogram (ECG) is a tool to evaluate cardiac involvement and the risk of arrythmias. We analysed the ECG of 465 HAT patients and compared them with the ECG of 61 healthy volunteers. In HAT patients the QTc interval was prolonged. This comprises a risk of fatal arrhythmias if new drugs with antiarrhythmic potential will be used. Further, repolarization changes and low voltage were more frequent than in healthy controls. This could be explained by an inflammation of the heart. Treatment of HAT was associated with appearance of repolarization changes but not with a QTc prolongation. These changes appear to be associated with the disease, but not with a specific drug. The main conclusion of this study is that heart involvement is frequent in HAT and mostly well tolerated. However, it can become relevant, if new compounds with antiarrhythmic potential will be used.

## Introduction

Human African Trypanosomiasis (HAT) or sleeping sickness evolves in two stages, the first or early (hemo-lymphatic) stage and the second or late (meningo-encephalitic) stage which is characterized by invasion of the central nervous system (CNS) by trypanosomes. Neuropsychiatric disturbances are the most prominent and best documented features of the disease [1]. Cardiac involvement plays an important role in American trypanosomiasis (Chagas' disease); however, in the African form, cardiac involvement has been suggested but has never been studied systematically in the first stage of the disease. Cardiac involvement has been observed in up to 73% of HAT patients in post mortem histological studies [2],[3]. Those findings are supported by the recent study of Blum *et al* [4] that showed cardiac alterations in 71% of second stage HAT patients, but are in contrast to previous studies, where ECG findings were reported in only 35–48% of the patients [5]–[7]. The latter studies included both first and second-stage HAT patients. A low prevalence of ECG findings in first stage disease could explain this discrepancy. Thus, cardiac involvement, as documented by ECG findings, may parallel CNS involvement and ECG findings could be used as additional tool for assessing the advancement of the disease.

Because of increasing resistance to the available drugs for HAT, new compounds or drug combinations are desperately needed. Evaluation of cardiotoxicity and the risk of cardiac arrhythmia is one parameter of drug safety, but without knowledge of heart involvement in HAT, cardiologic findings and drug-induced ECG alterations will be difficult to interpret.

Pentamidine administered intramuscularly is currently the primary treatment for first stage HAT. A large number of diamidine compounds have been synthesized in an attempt to develop an oral agent for this disease. DB289 (pafuramidine maleate) is one of these diamidine compounds. It can be orally administered and showed good efficacy against first stage HAT in Phase II trials. Since diamidines such as pentamidine have been shown to have arrhythmic potential [8], knowledge of HAT cardiopathy and scrupulous analysis of the potential cardiac effects of new antiparasitic drugs is essential.

The overall aim of the study was to assess the cardiac involvement in first stage HAT by ECG examination and to study the effect of different antiparasitic drugs on ECG findings.

## Methods

### Objectives

The objectives of this study were to assess the frequency and character of ECG findings in patients with first stage HAT and to compare them to healthy control subjects and to second stage HAT patients. Secondary objectives were to assess differences between administered HAT therapies, including ECG changes during and after treatment, and to discuss the findings with respect to clinical relevance and tolerability of medical therapy.

### Criteria of selection of trials

The objectives were to study and characterize ECG alterations in T.*b. gambiense* patients with respect to the stage of the disease (first versus second stage) and treatment induced alterations (baseline versus after treatment).

Only studies with clear definition of ECG criteria and complete ECG description (including QTc intervals), description of the stage of the disease and ECG before and after treatment were included. Using these criteria the following studies were not included:

- Studies on ECG alterations of patients infected with *T.b. rhodesiense* [9]–[11]
- Studies without indication, in which stage the patient was [5]–[7],[12],[13]
- Studies without ECG at baseline and after treatment alterations [3],[6],[7],[12],[13]
- Studies without clear inclusion/exclusion criteria or definition of ECG
  - Unclear, why only 28/100 patients had an ECG [3]
  - ECG description vague and no indication of QTc [5]–[7],[13],[14]

### Participants

Electrocardiograms (ECG) were performed prior to and following treatment and analyzed in a total of 523 participants; 406 were patients with first stage HAT, 56 with second stage HAT and 61 were healthy controls. Patients and controls from different clinical trials are included in the present analysis.

#### First stage patients

First stage HAT patients were recruited in the frame of phase II & III trials (International Standard Randomised Controlled Trial Number Register: ISRCTN85534673, 289-C-003 NCT 00802594, 289-C-006 NCT 00803933) conducted for the development of DB289 (pafuramidine maleate) in the Democratic Republic of Congo (Maluku, Vanga, Kikongo, Bandundu), Angola (Uíge) and Sudan (Yei). Between April 2002 and March 2007 a total of 415 patients older than 12 years were enrolled in these trials and 406 of them had sufficient ECG and treatment data to be included in this analysis. 177 patients received pentamidine (4 mg/kg daily) for 7 consecutive days by intramuscular injection. 64 patients received oral DB289 (200 mg total daily dose, divided twice daily) for 5 days and 165 patients had oral DB289 (200 mg total daily dose, divided twice daily) for 10 days.

#### Second stage patients and healthy controls

59 consecutive patients older than 15 years with parasitologically confirmed second stage HAT were enrolled in a separate prospective cohort study [4]. Three patients died either before or during treatment and were excluded from the current analysis. The control group consisted of 61 healthy persons with no previous history of HAT, matched for gender and age (+/−5 years). Data were collected between July 2004 and September 2005 in the DRC at the University Clinic in Kinshasa (Centre Neuro Psycho Pathologique), and at the Hôpital Evangélique de Vanga, Bandundu. Melarsoprol was the treatment of first line in patients with stage II HAT, 18 patients got melarsoprol intravenously (2.2 mg/kg/day) for 10 consecutive days [15] and 29 patients received the standard melarsoprol treatment [16] of increasing doses in 3 series of 3 days with rest periods of 7 days between the series. Eflornithine was reserved for patients with clinically advanced disease or after treatment failure with melarsoprol; 9 patients received eflornithine (400 mg/kg/day) via intravenous infusion over 14 days [17].

### Procedures

All HAT patients and healthy controls underwent a clinical assessment, including medical history, baseline physical examination, blood sampling for hematology and chemistry, and ECG. To estimate the normal intra-individual fluctuation of ECG parameters, two ECG recordings per subject were obtained at baseline. ECG changes appearing after treatment were compared to these intra-individual ECG changes. ECG data were also obtained during treatment in first stage HAT patients in all patients treated with DB 289 and in 40 patients treated with pentamidine. A clinical assessment was performed and the ECG was repeated after completion of treatment of HAT.

All ECG tracings were interpreted by a single reader using standardized criteria as described below. The ECG data for the first stage HAT patients were compared to the ECG data from the healthy controls and the second stage HAT patients.

### ECG interpretation

The PQ, QRS and QT intervals were measured manually by the principal investigator in three consecutive cycles and mean values were calculated. Measures of the intervals were performed in lead II, when feasible with lead V2 or I as second choice. QTc was calculated by the Bazett formula (QTc = QT/SQR (RR). QTc shorter than 440 ms and shorter than 460 ms were considered normal for men and women, respectively [18],[19] and because a QTc longer than 500 ms is known as predictor of torsades de pointes [19] both limits were used for the analysis. For overall ECG interpretation, the following criteria were used. Right atrial hypertrophy (RAH): p>2.5 mV; left atrial hypertrophy (LAH): p>120 ms; right ventricular hypertrophy (RVH): Sokolow index right: RV1 and S V5>1.05 mV, left ventricular hypertrophy (LVH): Cornell voltage: R aVL and SV3 men >2.8; women >2.0; peripheral low voltage: R I and RII and R III<1.6 mV; PR depression: >0.8 mV; ST elevation: >0.1 mV without notch, concave, from deep S; ST depression: >1 mV; repolarization changes: limb leads: discordant in at least one lead; precordial leads: negative in either V3, V4, V5 or V6. Early repolarization type: ST elevation concave, notch at the J point, positive T waves. Normal ECG included axis deviation, early repolarization type [20], ST elevation >0.1 mV without notch, concave, from deep S in precordial leads [20] and partial right bundle brunch block (RBBB). Minor ECG changes included intraventricular conduction delay, left or right atrial hypertrophy, isolated premature atrial or ventricular captures and left anterior hemiblock (LAHB). Major changes included: AV block I–III, low voltage, left and right ventricular hypertrophy, complete bundle branch block, PR depression, ST depression and repolarization changes.

### Ethical considerations

Written informed consent (illiterates signed by fingerprint) was obtained from all study participants. Ethical approval was granted by the Ethics Committees of the DRC, Angola, South Sudan and the Ethics Committee for the two cantons of Basel, Switzerland (Ethikkomission beider Basel).

### Data analysis

Data from the various studies were pooled into one single database that contained the clinical examination, demographic data and the ECG details. Analysis was done using the statistical software package STATA 9.0 (www.stata.com). All continuous variables are reported as mean±SD. A p-value<0.05 was considered statistically significant. Nominal variables were compared using the *Χ* ^2^- test. Comparisons between the patient groups and treatments were performed using the t-test, ANOVA plus Bonferroni correction , the Kruskal Wallis or Mann Whitney U tests where appropriate.

## Results

Patient baseline characteristics are summarized in Table 1. Age and gender distribution were similar among the disease stages, the HAT treatment groups and the healthy controls, respectively. Pyrimethamine-sulfadoxine (SP) was given as first line malaria treatment prior to HAT treatment in all centers with the exception of CNPP Kinshasa, where quinine was used as standard malaria treatment due to the high level of SP resistance in Kinshasa.

**Table 1:** Demographics and baseline description of the HAT patients and controls.

	Healthy controls		HAT stage 1				HAT stage 2			
			DB289		pentamidine		melarsoprol		eflornithine	
	n	(%)	n	(%)	n	(%)	n	(%)	n	(%)
**Demographics**										
Number of patients (Total N = 526)	61		229		177		47		9	
Age, mean (SD)	35 (13)		33 (13)		33 (14)		34 (12)		36 (11)	
Gender (male/female)	33/28	1.2	80/149	0.5	67/110	0.6	23/24	1.0	4/5	0.8
**Diagnostics**										
Malaria postive^a^	na		22	9.6	18	10.2	2	4.3	0	0.0
Filaria positive^a^	na		20	8.7	18	10.2	0	0.0	0	0.0
Pulse, mean (SD)	72 (11)		76 (14)		75 (13)		75 (14)		79 (16)	
Pulse>100	0		10	4.4	4	2.3	1	2.1	0	0.0
Blood pressure, mean systolic (SD)	111 (13)		107 (13)		108 (15)		105 (14)		109 (24)	
**Malaria pre-treatment** b										
Pyrimethamine/Sulfadoxine	na		203	88.6	154	87.0	29	61.7	0	0.0
Amodiaquine	na		4	1.7	6	3.4	0	0.0	0	0.0
Chloroquin	na		2	0.9	2	1.1	0	0.0	0	0.0
Quinine	na		9	3.9	7	4.0	18	38.3	9	100.0

na: not applicable, tests not done or data not available

a malaria and filaria testing are not standard and were only systematically applied in the DB289 trial series

b other anti-parasitic treatment was administered during the 3 days prior to initiation of HAT treatment

The ECG baseline intervals and characteristics are listed by disease stage in Table 2. At baseline, QTc prolongation, which was defined as >440 ms in men and, >460 ms in women, was observed in 11–13% of all HAT patients. These QTc values are considered to represent an increased risk for arrhythmia. Only one patient had a QTc interval over 500 ms, which is associated with an elevated risk for torsade de pointes. The proportion of major ECG findings indicating heart involvement was significantly lower (p-value = 0.0001) in first stage (53.5%) than in second stage HAT (69.5%). The QTc interval of HAT patients treated with melarsoprol following malaria treatment was 412 msec in the sulfadoxin/pyrimethamin group (SD 19) and 431 msec (SD 24) in the quinine group.

**Table 2:** ECG findings at baseline, by disease stage compared to healthy subjects.

	Healthy controls	HAT stage 1	HAT stage 2	p-value
	N = 61	N = 406	N = 56	
**ECG Intervals (msec; mean (SD))**				
PQ	163 (16)	159 (24)	169 (25)	0.008γ
QRS	82 (11)	80 (9)	82 (8)	0.076
QTc	403 (21)	421 (28)	423 (25)	<0.001α β
**ECG Findings (%)**				
atrial fibrillation/flutter	0.0	0.3	0.0	0.862
premature atrial capture	1.6	0.5	0.0	0.450
premature ventricular capture	1.6	0.7	3.6	0.285
bigeminus	0.0	0.2	0.0	0.871
AV block 1	3.3	3.7	7.1	0.449
AV block 2	0.0	0.0	0.0	1.000
RAH (Right atrial hypertrophy)	0.0	1.7	1.8	0.334
LAH (Left atrial hypertrophy)	1.6	2.5	5.4	0.394
RVH (Right ventricular hypertrophy)	3.3	5.2	3.6	0.732
LVH (Left ventricular hypertrophy)	1.6	2.0	1.7	0.977
pathologic Q	0.0	1.0	0.0	0.551
RBBB (Right bundle brunch block)	0.0	1.0	0.0	0.581
LBBB (Left bundle brunch block)	1.6	0.0	0.0	0.011
LAHB (Left anterior hemiblock)	1.6	1.7	0.0	0.584
RBBB and LAHB	0.0	0.5	1.7	0.469
repolarisation changes	6.6	35.2	32.1	<0.001α β
low voltage	6.7	20.0	30.4	0.005α β γ
QTc prolongation^a^	0	11.1	12.5	0.021α β
minor changes	16.4	9.6	7.1	0.193
major changes	19.7	53.5	67.9	<0.001α β γ

a for male >440 msec, female >460 msec

**α:** for healthy control vs HATstage 1 p<0.05

**β:** for healthy control vs HATstage 2 p<0.05

**γ:** for HAT stage 1 vs HATstage 2 p<0.05

Changes of ECG intervals and findings according to the different treatment groups are shown in the Table 3 and 4.

**Table 3:** ECG interval changes over time, by treatment.

	HAT Stage 1			HAT Stage 2		
	DB289	pentamidine	p-value	melarsoprol	eflornithine	p-value
	(N = 229)	(N = 177)		(N = 47)	(N = 9)	
**PQ msec, mean (SD)**						
Baseline	161 (26)	157 (20)	0.091	169 (26)	170 (20)	0.914
During treatment	163 (24)	155 (26)*	0.056	nd	nd	na
At end of treatment	166 (25)	161 (21)	0.037	168 (26)	158 (14)	0.269
p-value	0.810	0.683		0.557	0.714	
**QRS msec, mean (SD)**						
Baseline	79 (8)	80 (9)	0.237	82 (8)	79 (11)	0.337
During treatment	79 (8)	82 (9)*	0.033	nd	nd	na
At end of treatment	79 (8)	81 (13)	0.057	79 (6)	77 (5)	0.353
p-value	0.699	0.939		0.483	0.155	
**QTc msec, mean (SD)**						
Baseline	422 (26)	419 (30)	0.282	420 (23)	441 (29)	0.020
During treatment	419 (27)	413 (22)*	0.185	nd	nd	na
At end of treatment	419 (24)	417 (24)	0.406	427 (27)	408 (23)	0.053
p-value	0.355	0.417		0.183	0.021	

*: ECG during treatment was only performed in n = 40 patients in the pentamidine group

nd: ECGs not done

na: not applicable

**Table 4:** ECG changes during treatment (baseline vs end of treatment), by treatment.

	Intraindividual changes at baseline	HAT Stage 1			HAT Stage 2	
		DB289	Pentamidine	p-value	Melarsoprol	DFMO
	(N = 526)	(N = 229)	(N = 177)		(N = 47)	(N = 9)
**Repolarisation changes (%)**						
appearance/aggravation	1.9	5.7	4.5	0.300	29.8	44.4
disappearance/improvement	1.5	13.1	9.6	0.138	12.8	11.1
**Low voltage (%)**						
appearance	0.2	2.2	1.7	0.363	6.4	0
disappearance	0	1.3	1.7	0.625	4.3	0
**AV block I (%)**						
appearance	1.3	2.6	1.1	0.190	0	0
disappearance	0.8	1.3	0.6	0.872	2.1	0

During treatment, no patient in the DB289 or the pentamidine groups developed a QTc longer than 500 ms. One patient with a QTc longer than 500 ms at baseline had a normal QTc after treatment. In the group of second stage patients, one patient developed a significantly prolonged QTc interval during melarsoprol treatment.

The development or disappearance of AV block I consisted mostly of increases or decreases of a few milliseconds, usually from just below to just above the upper limit of normal (200 ms) or vise versa. During the treatment period in the DB289 group no relevant conduction problems such as AV block II or III or ventricular arrhythmias were seen. In the pentamidine group, two patients developed an AV block II (Type Wenckebach), which resolved spontaneously and was asymptomatic. One patient with second stage HAT developed a bigeminal rhythm during treatment with melarsoprol, which subsided after administration of corticosteroids. There were no further significant changes in rhythm or conduction, such as ventricular arrhythmia, appearance of AV block III or formation of bundle branch block, observed in ECG recordings during treatment compared to baseline or during treatment compared to after treatment.

## Discussion

To our knowledge this is the first study comparing ECG data of first and second stage HAT patients and also healthy controls. This study comprises ECG recordings of 523 participants, the largest number of patients to date in a population with HAT.

The mean QTc interval was significantly longer in HAT patients compared to healthy controls and increased slightly with the progression of the disease from first to second stage. As HAT itself was associated with QTc prolongation in more than 10% of patients, the additional risk of a drug with potential QTc prolongation properties has to be considered because of the risk of fatal arrhythmias. The mean QTc interval of the eflornithine group at baseline was longer than in the other groups. Eflornithine was given to patients with more advanced disease. In addition, these patients were treated with quinine before the baseline ECG was obtained, which has a known effect on QTc [21],[22]. Pretreatment with quinine in the melarsoprol group had an additional influence on QTc prolongation. Most patients in the other groups received SP for antimalarial treatment prior to obtaining baseline ECG recordings, and SP has a negligible effect on the QTc interval [23].

Cardiac involvement, as defined by major ECG alterations, was found in more than half of the patients with first stage HAT and in about two thirds in the second stage. Thus, ECG alterations are not a suitable additional tool for stage determination. Repolarization changes (35%), low voltage (20%) and QTc prolongation (11%) were the major abnormalities observed. Conduction delays, such as bundle branch block or AV block, and pathologic Q waves (signs of necrosis) were not found more often than in healthy controls. An explanation of the repolarization changes and the low voltage observed in the current study cannot be inferred from these data. However, observations from former histological studies showing a diffuse, interstitial lympho-mono-histiocytic infiltration and edema in the pericardium, myocardium, and endocardium without myocardial necrosis [2], [3], [24]–[27], support the hypothesis that repolarization changes and low voltage represent peri-myocarditis. The presence of pericardial involvement has been reported in a prior echocardiographic study, where pericarditis with pericardial effusion was demonstrated in 12% of patients [7]. In our study, repolarization changes were similar in both stages suggesting that peri-myocarditis appears early in the disease. The lower proportion of low voltage in first stage compared to second stage patients (20% versus 30.5%, p = 0.004) suggests that pericardial effusion becomes more significant later in the disease.

None of the patients with first stage HAT were diagnosed with congestive heart failure. In second stage HAT, NT-proBNP values were significantly higher in patients than in controls [4], and 23% of these patients had values that have been associated with an ejection fraction of less than 40% [28]. However, the overall relatively low NT-proBNP levels indicate that most HAT patients probably did not have clinically relevant congestive heart failure. Thus, heart failure appears infrequently and/or late in the disease progression of HAT.

In the following sections the effect of antiparasitic treatment to ECG findings will be discussed. The mean PQ intervals and QTc time did not increase during treatment of first stage disease in either the DB289 or the pentamidine group. The appearance and disappearance of AV block I was in the range of the normal intra-individual fluctuation in all treatment groups. However, the appearance of AV block II and bigeminal rhythm showed the potential of relevant conduction problems under treatment. Clinical case reports of conduction delays in patients with HAT have been published. A German tourist with *T.b. gambiense* infection developed transient AV block III, supraventricular tachycardia and ventricular premature captures (class Lown IV b) [29] and a British soldier infected with *T.b. rhodesiense* developed transient second degree heart block [30]. Based on unpublished observations, there is a minority of patients who die suddenly without explanation and sudden cardiac death cannot be ruled out [3]. An inflammatory process with accentuation in the conduction system has been observed after treatment in histological studies from HAT patients [6],[31],[32].

The appearance and disappearance of repolarization changes at end of treatment were comparable between the DB289 and the pentamidine group. These changes were significantly more frequent than the normal intra-individual fluctuation at baseline and could be due to an unspecific inflammatory reaction to antiparasitic treatment or to a direct cardiotoxic effect of the medication. Histological findings of an accentuation of the inflammatory response have been interpreted as an immune response to dying trypanosomes in treated HAT patients, and could be manifested as repolarization changes [33]. A specific cardiotoxic effect due to a single drug is unlikely to be responsible for the observed repolarization changes, as they were observed with all therapies studied. There was no evidence for other etiologies such as ventricular hypertrophy or coronary heart disease; these were young patients without angina pectoris before and after treatment. Thus, an increase in inflammation is a likely etiology for these transient and dynamic ECG changes that occurred in the setting of anti-parasitic treatment.

A higher percentage of major ECG alterations after treatment was observed in second stage patients than in first stage patients. A higher grade of inflammation of the heart is likely to occur with progression of the disease and explains the higher rate of treatment-related ECG abnormalities in the eflornithine group. The ECG alterations were well tolerated.

Limitations of this analysis were that HAT patients and healthy controls were enrolled in different clinical trials. However, the criteria of the interpretation of the ECG recordings were the same in all subjects and were performed and analyzed by the same physician.

### Conclusions

Cardiac involvement, as demonstrated by ECG alterations, appears early in the evolution of HAT and precedes CNS involvement. As HAT itself was associated with QTc prolongation in more than 10% of patients, the additional risk of a drug with potential QTc prolongation properties has to be considered because of the risk of fatal arrhythmias. DB289 and pentamidine treatment were not associated with prolongation of the QTc intervals and they had no obvious cardiotoxic effect. During treatment, ECG changes such as repolarization alterations occurred frequently, were not associated with one specific drug, and were more common in the second stage of the disease.

## Supporting Information

Alternative Language Abstract S1 Translation of the Abstract into German and French by Johannes Blum
(0.04 MB DOC)

Poster S1 Poster on preliminary results #1
(0.03 MB DOC)

Poster S2 Poster on preliminary results #2
(2.09 MB PPT)

Protocol S1 (0.34 MB PDF)
