# Supplementary material for: Cardiac Alterations in Human African Trypanosomiasis (T.b. gambiense) with Respect to the Disease Stage and Antiparasitic Treatment
Source: PLoS Negl Trop Dis. 2009 Feb 17;3(2):e383. doi: 10.1371/journal.pntd.0000383 (PMC2640099; doi:10.1371/journal.pntd.0000383)
Supplement: Alternative Language Abstract S1 — Translation of the Abstract into German and French by Johannes Blum (0.04 MB DOC) [file pntd.0000383.s001.doc]

**Zusammenfassung**

**Translation of the abstract in to German by Johannes Blum (main author)**

*Hintergrund*

Bei der afrikanischen Trypanosomiasis (Schlafkrankheit) dominieren neurologische Symptome, aber auch kardiologische Pathologien wurden beobachtet.

Wegen zunehmender Resistenzen gegenüber den verfügbaren Medikamenten werden neue Substanzen dringend benötigt. Im Rahmen der Überprüfung der Sicherheit müssen auch kardiologische Nebenwirkungen eines Medikamentes erfasst werden. Da aber bis jetzt systematische Studien über die kardiologischen Veränderungen, insbesondere von EKG Veränderungen fehlen, wird es schwierig sein, krankheitsbedingte von medikamentenbedingten kardiologischen Abnormitäten zu unterscheiden.

Ziele der Studie sind die Häufigkeit und Charakteristika von EKG Veränderungen im ersten Stadium der Schlafkrankheit zu erfassen, die Veränderungen mit denen von gesunden Kontrollpersonen und Patienten im zweiten Schlafkrankheitsstadium zu vergleichen sowie das Potential für EKG Veränderungen bei verschiedenen Schlafkrankheitsmedikamenten zu vergleichen.

*Methoden*

Im Rahmen von klinischen Studien, bei denen die Wirksamkeit und das Sicherheitsprofil von DB 289 und Pentamidin geprüft wurden, konnten 406 Patienten mit Schlafkrankheit im ersten Stadium in den Ländern Demokratische Republik Kongo, Angola und Sudan im Zeitraum 2002 bis 2007 in die Studie eingeschlossen werden. Die EKG dieser Patienten wurden mit gesunden Kontrollpersonen (n=61) sowie mit Schlafkrankheitspatienten (n=56) im zweiten Stadium verglichen.

*Resultate*

Sowohl bei Patienten im ersten wie im zweiten Stadium der Schlafkrankheit wurden eine Verlängerung des QTc Intervalls, Repolarisationsveränderungen sowie eine Niedervoltage signifikant häufiger beobachtet als bei den gesunden Kontrollpersonen. Unter der Behandlung sowohl mit DB 289 als auch mit Pentamidin wurden Repolarisationsveränderungen, aber keine Verlängerung des QTc Intervalls festgestellt.

*Schlussfolgerungen*

Schon früh im Verlauf der Schlafkrankheit kommt es zu einer Herzbeteiligung, die mittels EKG Veränderungen dokumentiert werden kann. Eine Verlängerung des QTc Intervalls erhöht das Risiko von Herzrhythmusstörungen, vor allem wenn eine Substanz verwendet wird, die ihrerseits ein Potential für Herzrhythmusstörungen hat. Während der Behandlung der Schlafkrankheit kam es häufig zu EKG Veränderungen, die am ehesten auf eine Myo- Perikarditis hinweisen und nicht auf ein spezifisches Medikament zurückzuführen sind.

**Résumé**

**Translation of the abstract in to French by Johannes Blum (main author)**

*Introduction*

Les symptômes neurologiques dominent le tableau clinique de la trypanosomiase africaine, mais une atteinte cardiaque a été suspectée. Vu la résistance croissante aux médicaments disponibles contre la trypanosomiase, il y a un besoin urgent en nouvelles substances. Lors de l’évaluation des effets indésirables, une évaluation de la cardiotoxicité nécessite des connaissances sur l’atteinte cardiaque dans la trypanosomiase pour distinguer les altérations causées par l’atteinte cardiaque de la trypanosomiase des effets cardiotoxiques médicamenteux.

Les objectifs de cette étude sont les suivants :

- l’évaluation de la fréquence et le caractère des altérations de l’ECG des trypanosomés en premier stade,
- la comparaison de ces altérations entre les stades un et deux,
- la comparaison de ces altérations entre des patients souffrant de la trypanosomiase et des sujets en bonne santé,
- l’évaluation de la cardiotoxicité des différentes substances.

*Méthodes*

406 patients en premier stade de la trypanosomiase étaient inclus entre 2002 et 2007 en République Démocratique Congo, Angola et Soudan dans le cadre des études comparant l’efficacité et la sécurité de pentamidine et DB 289. Les ECG de ces patients ont été comparés aux sujets en bonne santé (n=61) et aux trypanosés en deuxième stade (n=56).

*Résultats*

Une prolongation de l’intervalle QTc, des changements de la repolarisation et un low voltage étaient significativement plus fréquents chez les trypanosés en premier et en deuxième stade que chez les sujets en bonne santé. Le traitement de la trypanosomiase en premier stade était associé aux altérations de la repolarisation dans la même mesure dans le groupe pentamidine que dans le groupe DB 289. L’intervalle QTc n’a pas changé durant le traitement.

*Conclusions*

Une atteinte cardiaque, démontrée par des altérations dans l’ECG apparaît dans les débuts de l’évolution de la trypanosomiase. La prolongation de l’intervalle QTc pourrait - si associée avec un médicament cardiotoxique - mener à des arythmies graves.

Souvent, des altérations de l’ECG telles que des changements de la repolarisation apparaissent, mais ces changements sont attribués plutôt à une myo-péricardite trypanosomienne qu’à un médicament spécifique.

**Abstract**

*Background*

In Human African Trypanosomiasis, neurological symptoms dominate and cardiac involvement has been suggested. Because of increasing resistance to the available drugs for HAT, new compounds are desperately needed. Evaluation of cardiotoxicity is one parameter of drug safety, but without knowledge of the baseline heart involvement in HAT, cardiologic findings and drug-induced alterations will be difficult to interpret. The aims of the study were to assess the frequency and characteristics of electrocardiographic findings in the first stage of HAT, to compare these findings to those of second stage patients and healthy controls and to assess any potential effects of different therapeutic antiparasitic compounds with respect to ECG changes after treatment.

*Methods*

406 patients with first stage HAT were recruited in the Democratic Republic of Congo, Angola and Sudan between 2002 and 2007 in a series of clinical trials comparing the efficacy and safety of the experimental treatment DB289 to the standard first stage treatment, pentamidine. These ECGs were compared to the ECGs of healthy volunteers (n=61) and to those of second stage HAT patients (n=56)

*Results*

In first and second stage HAT, a prolonged QTc interval, repolarization changes and low voltage were significantly more frequent than in healthy controls. Treatment in first stage was associated with repolarization changes in both the DB289 and the pentamidine group to a similar extent. The QTc interval did not change during treatment.

*Conclusions*

Cardiac involvement in HAT, as demonstrated by ECG alterations, appears early in the evolution of the disease. The prolongation of the QTC interval comprises a risk of fatal arrhythmias if new drugs with an additional potential of QTC prolongation will be used. During treatment ECG abnormalities such as repolarization changes consistent with peri-myocarditis occur frequently and appear to be associated with the disease stage, but not with a specific drug.
