# Supplementary material for: Cardiac Alterations in Human African Trypanosomiasis (T.b. gambiense) with Respect to the Disease Stage and Antiparasitic Treatment
Source: PLoS Negl Trop Dis. 2009 Feb 17;3(2):e383. doi: 10.1371/journal.pntd.0000383 (PMC2640099; doi:10.1371/journal.pntd.0000383)
Supplement: Poster S1 — Poster on preliminary results #1 (0.03 MB DOC) [file pntd.0000383.s002.doc]

**Phase III trial of pafuramidine maleate (DB289), a novel, oral drug for treatment of first stage sleeping sickness**

Christian Burri^1^, Gabriele Pohlig^1^, Sonja Bernhard^1^, Alain Mpanya Kabeya^2^, Jean-Pierre Fina Lubaki^3^, Alfred Mpoo Mpoto^3^, Kambau Manesa Deo Gratias^4^, Florent Mbo Kuikumbi^2^, Alain Fukinsia Mintwo^2^, Auguy Kayeye Munungi^2^, Jose Tito Bage^5^, Stephen Macharia^6^, Constantin Miaka Mia Bilenge^2^, Victor Kande Betu Ku Mesu^2^, Jose Ramon Franco^6^, Ndinga Dieyi Dituvanga^5^ & Carol Olson^7^.

^1^Swiss Tropical Institute, Swiss Center for International Health, Basel, Switzerland; ^2^Programme Nationale de Lutte contre la Trypanosomiase Humaine Africaine, Kinshasa, R.D. Congo; ^3^Evangelic Hospital, Vanga, R.D. Congo; ^4^Evangelic Hospital, Kikongo, R.D. Congo; ^5^Instituto de Combate e de Controlo das Tripanossomíases, Luanda, Angola; ^6^Malteser International, Yei, South Sudan; and ^7^Immtech Pharmaceuticals Inc., Vernon Hills IL, USA.

Only a very limited number of drugs are available for treatment of sleeping sickness and none of them is applicable by the oral route. After successful preclinical testing, the oral prodrug pafuramidine was selected for clinical development for the treatment of first stage sleeping sickness in the year 2000 by the international consortium to discover new drugs for the treatment of parasitic diseases. The consortium is lead by the University of North Carolina, Chapel Hill, USA, and funded through the Bill & Melinda Gates Foundation.

The compound successfully underwent extensive evaluation in several Phase I (healthy volunteers) and Phase II (proof of concept in patients) clinical trials. The clinical studies demonstrated the efficacy and good tolerability of pafuramidine, in particular as compared to standard treatment pentamidine. In the Phase II trials, the dosing regimen for pafuramidine was extended from 5 to 10 days in order to achieve sustained efficacy.

A protocol for a pivotal Phase III confirmatory trial was developed in close collaboration with the US FDA. This open-label (sponsor-blinded), randomized, controlled clinical trial comparing pafuramidine with pentamidine was initiated in August 2005. Patients were enrolled in four centers in the Democratic Republic of Congo and in one center each in Angola and South Sudan. Enrollment of 274 patients, including adolescents and pregnant and lactating women, was completed in March 2007. Follow up of the patients will continue for two years. An interim analysis by the DSMB is planned in July 2007 after half of the recruited subjects have undergone the 12 month follow up examination. Should the outcome of this analysis be satisfactory, all subjects will undergo the 12 month follow up evaluation, the primary endpoint for the trial, and a registration dossier for submission to the US FDA will be prepared.

Results will be presented on the safety and preliminary efficacy of pafuramidine.
