# Supplementary material for: Cardiac Alterations in Human African Trypanosomiasis (T.b. gambiense) with Respect to the Disease Stage and Antiparasitic Treatment
Source: PLoS Negl Trop Dis. 2009 Feb 17;3(2):e383. doi: 10.1371/journal.pntd.0000383 (PMC2640099; doi:10.1371/journal.pntd.0000383)
Supplement: Poster S2 — Poster on preliminary results #2 (2.09 MB PPT) [file pntd.0000383.s003.ppt]

## Slide 1
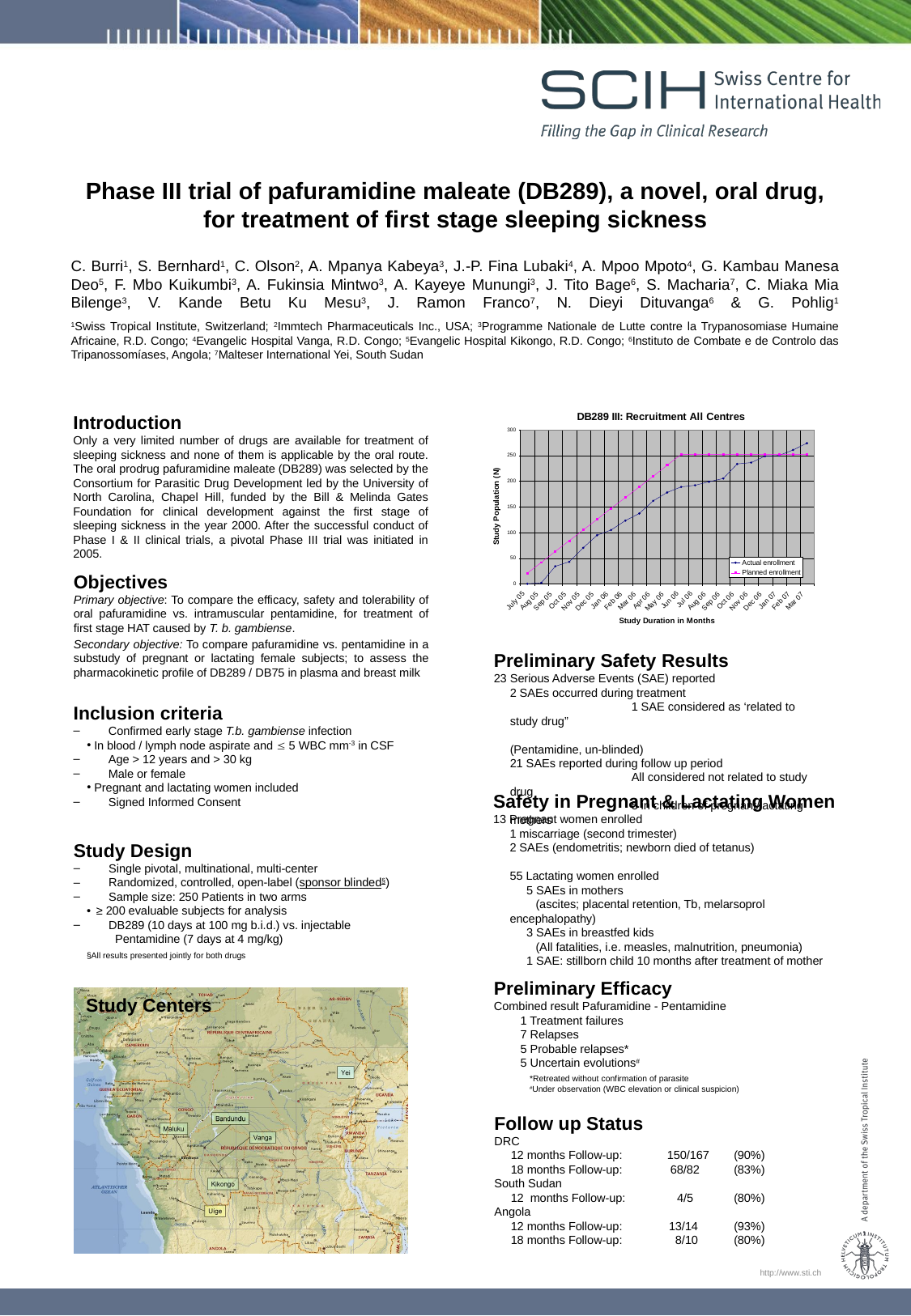

# Phase III trial of pafuramidine maleate (DB289), a novel, oral drug, for treatment of first stage sleeping sickness
C. Burri1, S. Bernhard1, C. Olson2, A. Mpanya Kabeya3, J.-P. Fina Lubaki4, A. Mpoo Mpoto4, G. Kambau Manesa Deo5, F. Mbo Kuikumbi3, A. Fukinsia Mintwo3, A. Kayeye Munungi3, J. Tito Bage6, S. Macharia7, C. Miaka Mia Bilenge3, V. Kande Betu Ku Mesu3, J. Ramon Franco7, N. Dieyi Dituvanga6 & G. Pohlig1
1Swiss Tropical Institute, Switzerland; 2Immtech Pharmaceuticals Inc., USA; 3Programme Nationale de Lutte contre la Trypanosomiase Humaine Africaine, R.D. Congo; 4Evangelic Hospital Vanga, R.D. Congo; 5Evangelic Hospital Kikongo, R.D. Congo; 6Instituto de Combate e de Controlo das Tripanossomíases, Angola; 7Malteser International Yei, South Sudan
Introduction
Only a very limited number of drugs are available for treatment of sleeping sickness and none of them is applicable by the oral route. The oral prodrug pafuramidine maleate (DB289) was selected by the Consortium for Parasitic Drug Development led by the University of North Carolina, Chapel Hill, funded by the Bill & Melinda Gates Foundation for clinical development against the first stage of sleeping sickness in the year 2000. After the successful conduct of Phase I & II clinical trials, a pivotal Phase III trial was initiated in 2005.
Objectives
Primary objective: To compare the efficacy, safety and tolerability of oral pafuramidine vs. intramuscular pentamidine, for treatment of first stage HAT caused by T. b. gambiense.
Secondary objective: To compare pafuramidine vs. pentamidine in a substudy of pregnant or lactating female subjects; to assess the pharmacokinetic profile of DB289 / DB75 in plasma and breast milk
Preliminary Safety Results
23 Serious Adverse Events (SAE) reported
	2 SAEs occurred during treatment
		1 SAE considered as ‘related to study drug”
			(Pentamidine, un-blinded)
	21 SAEs reported during follow up period
		All considered not related to study drug
		5 in children of pregnant/lactating mothers
Inclusion criteria
 Confirmed early stage T.b. gambiense infection
 In blood / lymph node aspirate and ≤ 5 WBC mm-3 in CSF
 Age > 12 years and > 30 kg
 Male or female
 Pregnant and lactating women included
 Signed Informed Consent
Safety in Pregnant & Lactating Women
13 Pregnant women enrolled
	1 miscarriage (second trimester)
	2 SAEs (endometritis; newborn died of tetanus)
55 Lactating women enrolled
	5 SAEs in mothers
		(ascites; placental retention, Tb, melarsoprol encephalopathy)
	3 SAEs in breastfed kids
		(All fatalities, i.e. measles, malnutrition, pneumonia)
	1 SAE: stillborn child 10 months after treatment of mother
Study Design
 Single pivotal, multinational, multi-center
 Randomized, controlled, open-label (sponsor blinded§)
 Sample size: 250 Patients in two arms
≥ 200 evaluable subjects for analysis
 DB289 (10 days at 100 mg b.i.d.) vs. injectable
 Pentamidine (7 days at 4 mg/kg)
§All results presented jointly for both drugs
Preliminary Efficacy
Combined result Pafuramidine - Pentamidine
	1 Treatment failures
	7 Relapses
	5 Probable relapses*
	5 Uncertain evolutions#
		*Retreated without confirmation of parasite
		#Under observation (WBC elevation or clinical suspicion)
Study Centers
Follow up Status
DRC
	12 months Follow-up: 	150/167 	(90%)
	18 months Follow-up: 	68/82 	(83%)
South Sudan
	12 months Follow-up:	 4/5 	(80%)
Angola
	12 months Follow-up:	13/14 	(93%)
	18 months Follow-up: 	 8/10 	(80%)
http://www.sti.ch
