# Supplementary material for: Cardiac Alterations in Human African Trypanosomiasis (T.b. gambiense) with Respect to the Disease Stage and Antiparasitic Treatment
Source: PLoS Negl Trop Dis. 2009 Feb 17;3(2):e383. doi: 10.1371/journal.pntd.0000383 (PMC2640099; doi:10.1371/journal.pntd.0000383)
Supplement: Protocol S1 — (0.34 MB PDF) [file pntd.0000383.s004.pdf]

IMMTECH

Swiss Tropical Institute
Institut Tropical Suisse
Schweizerisches Tropeninstitut

Swiss Centre for
International Health

Clinical Protocol

International Randomized Controlled Phase 3 Trial of DB289 versus Pentamidine for the Treatment of First Stage Human African Trypanosomiasis

Protocol Number	289-C-010, C05-010
Document Date:	26 May 2005
Study Coordinator CRO:	Gabriele Pohlig, PhD<br>Swiss Tropical Institute<br>Socinstrasse 57,<br>CH-4002 Basel, Switzerland<br>Tel +41 61 225 26 62<br>Fax +41 61 225 26 78<br>E-mail: Gabriele.Pohlig@unibas.ch
Sponsor Contact:	Carol Olson, MD, PhD<br>Immtech International, Inc.<br>150 Fairway Drive, Suite 150<br>Vernon Hills, Illinois, 60061 USA<br>Tel +1 847-573-0033<br>FAX +1 847-573-8288<br>E-mail: colson@immtech-international.com

Funding for this trial is provided by a grant from the Bill and Melinda Gates Foundation. The grant is administered by the University of North Carolina, Chapel Hill. USA.

CONFIDENTIALITY STATEMENT

This document contains information which is confidential and therefore is provided to you in confidence for review by you, your staff, an applicable Ethics Committee / Institutional Review Board and regulatory authorities. It is understood that this information will not be disclosed to others without prior written approval from Immtech International Inc., except to the extent necessary to obtain informed consent from those persons to whom the drug may be administered.

1.1 Signatures of Agreement for Protocol

____ __________

R.R. Tidwell, PhD, Grant Administrator and Program Scientific Director — Date
University of North Carolina, Chapel Hill, NC 27599, USA

____ __________

C.A. Olson, MD, PhD, Vice President and Chief Medical Officer — Date
Immtech International, Inc., Vernon Hills, IL 60061, USA

____ __________

J.L. Allen, PhD, Vice President of Regulatory Affairs — Date
Immtech International, Inc., Vernon Hills, IL 60061, USA

____ __________

Chr. Burri, MSc, PhD, Study Director, Head of CRO — Date
Swiss Tropical Institute, CH-4002 Basel, Switzerland

____ __________

J. Blum, MD, Principal Investigator — Date
Swiss Tropical Institute, CH-4002 Basel, Switzerland

1.2 Abstract

Human African Trypanosomiasis (HAT), or sleeping sickness, caused by *Trypanosoma brucei gambiense* has made a spectacular return during the last decade, and in many places the number of infected individuals largely surpasses the capacities of the treatment centers. Treatment of the disease remains unsatisfactory. All currently used drugs must be administered parenterally, treatment is lengthy, and adverse drug reactions frequent. There are currently no drugs that are easily administered and have low toxicity, and might thus be used as tools to support disease control.

The study objectives are to compare the safety and efficacy of DB289, a new, orally administered dication prodrug to pentamidine intramuscular (i.m.) injection for the treatment of first stage HAT. The project will be executed within the framework of an international consortium consisting of several partners from academia, industry and from the Ministries of Health of the participating countries.

Design

This is a multi-center, multi-country open label (sponsor blinded), parallel group, comparator controlled and randomized Phase 3 trial. Approximately 250 subjects = 12 years of age are to be enrolled at 5-7 sites in order to obtain approximately 200 clinically evaluable subjects in the per protocol population at the primary endpoint (12 months post treatment).

Objectives

1. The primary objective of this study is to compare the efficacy, safety and tolerability of oral DB289 versus intramuscular pentamidine, for treatment of first stage HAT caused by *T. b. gambiense*.

2. In a substudy of pregnant or lactating female subjects, to compare the efficacy, safety and tolerability of DB289 versus pentamidine for treatment of first stage HAT, and to assess the pharmacokinetic profile of DB289 and DB75 in plasma and breast milk in this population.

2.0 Table of Contents

1.0 Clinical Protocol 1

1.1 Signatures of Agreement for Protocol 2

1.2 Abstract 3

2.0 Table of Contents 4

List of Tables 7

List of Figures 7

Abbreviations 8

3.0 Introduction 10

3.1 Overview of Human African Trypanosomiasis 10

3.2 Treatment of HAT 11

3.3 Development of Novel Aromatic Dicationic Analogs of Pentamidine to Treat Microbial Infections 13

3.4 Oral Activity of the Prodrug DB289 against Experimental African Trypanosomiasis 13

3.5 Safety and Pharmacokinetics of Single Oral Doses of DB289 in Humans 15

3.6 Safety and Pharmacokinetics of Multiple Oral Doses of DB289 in Humans 16

3.7 Preliminary Results of Pilot Phase 2a Trial in the Treatment of First Stage HAT 17

3.8 Preliminary Results of Comparative Phase 2b Trial in the Treatment of First Stage HAT 18

3.9 Overview of the Safety and Tolerability of DB289 20

3.9.1 Serious and Significant Adverse Events 21
3.9.2 Incidence and Severity of Individual Adverse Events 21
3.9.3 Electrocardiographic Findings 22
3.9.4 Pregnancies in First Stage HAT Trials 23
3.9.5 Reproductive Studies in Animals and Relevance to Treatment of Pregnant and Lactating Women 23

4.0 Objectives 24

4.1 Primary Objective 24

4.2 Secondary Objective 24

5.0 Investigational Plan 24

5.1 Overall Study Design 24

5.1.1 Discussion of Study Design and Choice of Control Groups 26

5.2 Study Population 28

5.2.1 Criteria for Inclusion 28
5.2.2 Criteria for Exclusion 28
5.2.3 Criteria for Discontinuation 29

5.3 Treatments ... *30*
5.3.1 Treatments Administered ... 30
5.3.2 Identification and Handling of Investigational Product ... 30
5.3.3 Method of Assigning Subjects to Treatment Groups ... 31
5.3.4 Selection of Doses and Timing of Dose ... 32
5.3.5 Blinding ... 33
5.3.6 Prior and Concomitant Therapy ... 33
5.3.7 Treatment Compliance ... 33

5.4 Study Plan ... *34*
5.4.1 Efficacy and Safety Measurements Assessed and Flow Chart ... 34

5.5 Efficacy and Safety Variables ... *43*
5.5.1 Appropriateness of Measurements ... 43
5.5.2 Efficacy Variables ... 43
5.5.3 Safety Variables ... 44
5.5.4 Drug Concentration Measurements ... 45

6.0 Statistical Methods ... 46

6.1 Statistical and Analytical Plan ... *46*
6.1.1 Data Sets Analyzed ... 46
6.1.2 Demographics and Other Baseline Characteristics ... 47
6.1.3 Efficacy Analyses ... 47
6.1.4 Safety Analyses ... 49
6.1.5 Interim Analyses ... 50
6.1.6 Determination of Sample Size ... 50

7.0 Protocol Deviations ... 51

8.0 Ethics and Regulatory Requirements ... 51

8.1 Independent Ethics Committee (IEC) or Institutional Review Board (IRB) ... *51*

8.2 Ethical Conduct of the Study ... *51*

8.3 Subject Information and Consent ... *52*

8.4 Subject Confidentiality ... *52*

8.5 Data and Safety Monitoring Board (DSMB) ... *52*

8.6 Investigator(s) and Study Site(s) ... *52*

9.0 Data Quality Assurance ... 52

10.0 Other Administrative and Regulatory Procedures ... 52

10.1 Source Documents, Case Report Form Completion, Monitoring and Inspections, and Maintenance of Records ... *52*

10.2 Completion of the Study ... *53*
10.2.1 Final Report ... 53
10.2.2 Use of Information ... 53

10.3 Publication ... *53*

10.4 Sponsor Information ... *53*

10.5 Contract Research Organization ... *53*

10.6 Clinical Supply Management 53

10.7 Laboratories 54
10.7.1 Clinical Laboratory Tests and Normal Laboratory Values 54
10.7.1 Identification of Laboratories 54
10.7.2 Clinical Supply Management 54

11.0 Country Specific Investigator's Signature Page 55

Appendix 1 Guidance to Investigator for Exclusion Criteria Number 2 56

Appendix 2 Glasgow Coma Scale (adapted) 57

Appendix 3 Toxicity Grading Scale 58

Appendix 4 Administrative Procedures for the Reporting of Adverse Events 62

Appendix 5 Ethical Considerations and Human Subject Protection 65

Appendix 6 Other Administrative and Regulatory Procedures 72

Appendix 7 Overdose Instructions 76

Appendix 8 Instructions for the Collection, Handling, and Shipping of Samples. 77

Appendix 9 Publication Policy 80

Appendix 10 Essential Documents 81

Appendix 11 Efficacy and Safety Measurements Assessed and Flow Chart 82

Appendix 12 Grading of Clinical Signs and Symptoms of HAT[59] 84

REFERENCES 85

List of Tables

Table 3.7.a **Mean (±SEM) Pharmacokinetic Parameters of DB289 and DB75 ....18**
Table 3.8.a **Efficacy in Phase 2 Trypanosomiasis Trials 20**
Table 3.9.2.b **Adverse Events reported in Phase 2b clinical trials of first stage HAT, without regard to association to study drug 22**
Table 5.4.1.4.a **Classification of first stage patients during follow up & actions taken 40**
Table 5.4.1.6.a **Toxicity Grading and Actions Taken in Response to Toxicity....42**
Table 5.5.2.a **Clinical Response Definitions 44**

List of Figures

Figure 5.1.a **Study Design Schematic....25**

Abbreviations

AIDS	Acquired immune deficiency syndrome
AUC	Area under the curve
BID	Twice daily
CATT	Card agglutination test for trypanosomes
CDTC	Centre de Dépistage, Traitement et Contrôle
CFR	Case fatality rate
Cl /F	Apparent oral clearance
C_{max}	Maximal concentration
C_{min}	Minimal concentration
CNS	Central nervous system
CRF	Case report form
CSF	Cerebrospinal fluid
DSMB	Data and safety monitoring board
ECG	Electrocardiogram
HAT	Human African trypanosomiasis
HIV	Human immunodeficiency virus
i.m.	Intramuscular administration
ITT	Intention to treat
i.v.	Intravenous administration
ICCT	Instituto de Combate e de Controlo da Tripanossomíase, Angola
K_{el}	Elimination constant
LOCF	Last observation carried forward
MSF	Medecins Sans Frontières
m-AECT	Mini-anion exchange centrifugation technique
mITT	Modified intention to treat
n.d.	not done
Neg	Negative
PCP	*Pneumocystis jiroveci* pneumonia
PCR	Polymerase chain reaction
Pos	Positive
PNLTHA	Programme Nationale de Lutte contre la Trypanosomiase Humaine Africaine

QD	Once daily
REM	Rapid eye movement
s.c.	Subcutaneous administration
SOREMP	Sleep onset rapid eye movement periods
STI	Swiss Tropical Institute
T_{max}	Time to maximal concentration
ULN	Upper limit of normal
UNC	University of North Carolina, Chapel Hill, USA
WBC	White blood cells (leukocytes)
WHO	World Health Organization
WNL	Within normal limits

3.0 Introduction

3.1 Overview of Human African Trypanosomiasis

Human African Trypanosomiasis (HAT), or sleeping sickness, caused by the protozoan parasites *Trypanosoma brucei gambiense* (West African form of the disease) and *Trypanosoma brucei rhodesiense* (East African form of the disease) affects 36 African countries where all or part of the population is at risk of infection. Some 60 million people live at risk of contracting the disease, but no more than 3 to 4 million are under adequate surveillance. WHO estimates that at least 300,000 individuals are infected, and the incidence of reported new cases is approximately 20,000 – 40,000, which certainly is an underestimation. HAT due to *T. b. gambiense* is characterized by a chronic progressive course, which may last from months to several years before death occurs. In the form due to *T .b. rhodesiense*, the disease is usually acute and death occurs within weeks or months after infection.

The initial symptoms, such as headache, general malaise and fever, are very non-specific and thus often misinterpreted or overlooked. In the early hemolymphatic stage of the disease, fever, headache, joint pains, weight loss and pruritus are common symptoms. Thereafter a generalized lymphadenopathy develops. The posterior cervical lymph nodes often enlarge ("Winterbottoms sign") and can be easily palpated. All these signs and symptoms will generally become worse as time passes. Intermittent headaches and fevers will become persistent. Generalized endocrine disorders like reduced libido, amenorrhea, abnormal thirst or appetite, and prominent anemia are frequent. Thrombocytopenia is usual in both forms of the disease[1]. The liver and the spleen may be slightly enlarged and a localized edema may be observed in the eyelids, perineum and the skin of the back.

The onset of the clinically different second or late stage is defined by the invasion of the central nervous system (CNS) by trypanosomes. It results in a chronic meningoencephalitis; the meninges are infiltrated with lymphocytes, plasma cells and occasional morular (Mott) cells. The inflammatory cell infiltrate extends along the Virchow-Robin spaces into the substance of the brain producing the characteristic picture of perivascular cuffing[1]. The number of white blood cells (WBC), as well as the protein content is elevated in the cerebrospinal fluid (CSF) after invasion of the CSF by trypanosomes. This observation is currently used for discrimination of first and late stage patients: arbitrarily, if more than 5 WBC per mm^3 and/or protein content above 25 mg per 100 mL (method of Siccard & Cantaloube) and/or trypanosomes are found in the CSF, the patient is considered to be late stage and treated with melarsoprol[2]. Recent investigations have indicated that the WBC count is more reliable than the protein determination[3]. In addition, an alternative WBC count cut off of 20 cells has been suggested, based on the assumption that the higher rate of treatment failures will be balanced by the decreased use of dangerous second stage drugs[4,5]. The Angolan

authorities have already adopted this strategy for staging of patients, but so far no data have been published.

Early signs of late infection are changes of the personality and behavior, and may be very subtle. Speech may become indistinct and slow, and frequently extrapyramidal signs occur, with tremors of the tongue and the fingers.

The most impressive sign is a deregulation of the 24-h distribution of the sleep-wake alternation and an alteration of the sleep structure, with frequent sleep onset rapid eye movement (REM) periods (SOREMPs). Epileptiform seizures, euphoria and maniacal changes are observed and the patient becomes indifferent to his environment. The final phase is characterized by progressive mental deterioration and general wasting. Death results from the sleeping sickness itself, concurrent infections, often pneumonia, or malnutrition.

3.2 Treatment of HAT

Only a very limited number of drugs are available for treatment of the disease and there is no vaccination. For treatment of first stage *T.b. gambiense* HAT, pentamidine administered by the i.m. route is the drug of choice; for *T. b. rhodesiense*, suramin is used. The organo-arsenical compound melarsoprol (Arsobal®) is the principal drug used in the late stage *T. b. gambiense* HAT. Eflornithine® (α-difluoromethylornithine) was registered in 1990 by the FDA for use against *T. b. gambiense* HAT[6]. Its major drawback is the complicated application requiring sophisticated logistics, which is beyond the possibilities of most facilities.

Pentamidine is a synthetic aromatic diamidine with a molecular weight of 340 g mol^{-1} (base), or 593 g mol^{-1} (isethionate)[7], and the pKa is 11.4. It has an established place in the treatment of trypanosomiasis due to *T. b. gambiense*, antimony-resistant leishmaniasis and *Pneumocystis jiroveci* pneumonia (formerly known as *Pneumocystis carinii* pneumonia, or PCP)[7]. Until recently, two salts of the molecules were available: pentamidine isethionate (Pentacarinat®, Aventis) and methanesulfonate (Lomidine®, formerly Rhône-Poulenc Rorer), but the latter is no longer produced. One vial costs about US$ 30. Pentamidine is given free of charge by Aventis to WHO for the treatment of first stage HAT and is available through WHO or MSF either free or for a modest charge. The most commonly used dosage regimen of pentamidine isethionate is 4 mg kg^{-1} daily or on alternate days for 7 to 10 injections.

Generally, pentamidine is well tolerated, especially as an intramuscular injection. However, minor adverse reactions are common[8]: Immediate adverse drug reactions include hypotension (9.6%) with dizziness, sometimes collapse and shock. The frequency of a hypotensive reaction can be as high as 75%, if the drug is given as an intravenous injection. Simultaneous application of dextran and a prolonged infusion time may alleviate this reaction, but close monitoring of pulse rate and blood pressure are still mandatory. Therefore the drug is usually given by the i.m. route for treatment of first

stage HAT. Nausea and/or vomiting are reported in 2.4%. Local reactions at the site of injection include pain (18.3%) and sterile abscesses or necrosis (6.7%) after i.m. application. Systemic reactions are azotemia due to a nephrotoxic effect (23%), leucopenia (14.5%), abnormal findings in liver function tests (11%), hypoglycemia (8.4%) and hyperglycemia (5%). Persistent manifestation of diabetes is rare, but is the most important long term consequence[9,10]. Adverse reactions to pentamidine are usually reversible. There is no clinical or laboratory evidence for mutagenicity or fetotoxicity[11].

The drug accumulates extensively and trough concentrations increase progressively without achieving steady state throughout treatment. In patients who received multiple doses of 3 mg kg^{-1} of pentamidine for PCP, the elimination after the first dose followed a three compartment model and the terminal half life ($t_{1/2}\gamma$) was estimated at 29 h. After the last of an average of 13 daily injections, the mean elimination half-life was prolonged to about 12 days and pentamidine could still be detected in plasma six weeks after the last application[12]. The apparent volume of distribution is extremely large, 11.850 L after single dose application[13] and 35.000 L after multiple dosing[12].

The results found in patients treated for *T .b. gambiense* with 10 i.m. injections of pentamidine methanesulfonate (3.5 - 4.5 mg kg^{-1} on alternate days) are comparable. The maximum plasma levels were generally reached within 1 hour after injection and varied extensively (420 – 13,420 nmol l^{-1}). The median plasma concentration after the last dose was about five times higher than after the first. The median half-lives associated with the first, second and third phase were 4 minutes, 6.5 hours and 512 hours, respectively[14].

Tissue binding is high and total plasma protein binding is estimated at 70%[13]. Pentamidine binds strongly and extensively to lysosomes[15], and it was thought to be deposited in tissues, mostly the kidneys and liver[16]. This finding is of interest since nephrotoxicity is the most common adverse drug reaction of the drug[17].

Renal clearance only accounts for about 2%[12] to 12%[13,17] of the plasma clearance. Therefore dose adjustment is not recommended for renal impairment[12]. Pentamidine is converted to at least seven primary metabolites by the cytochrome P450 dependent oxygenases in rat liver homogenates and rat liver microsomes[18], and only 1% of the drug can be found unchanged in the urine and that metabolism was the major route of drug elimination mainly into the urine[17]. The two main metabolites, the 2- and 3-pentanol analogues of pentamidine, were found to be conjugated with sulphate or glucuronic acid[19,20].

The mode of action of pentamidine is unknown. Mechanisms that may play a role have been reviewed by Wang[21]. In summary, various bindings to nucleic acids, disruption of kinetoplast DNA, inhibition of RNA-editing in trypanosomes and inhibition of mRNA trans-splicing may be involved. The drug has also been shown to inhibit trypanosomal S-adenosyl-L-methionine decarboxylase, thus interfering with polyamine biosynthesis[22], but recent findings suggest that inhibition of this enzyme is not the main mode of action[23]. Additionally, pentamidine interacts with nucleic acids, thus affecting DNA

biosynthesis[9]. It has also been shown that the drug inhibits the plasma-membrane Ca^{2+}-ATPase of the parasites[24]. The drug is actively transported into bloodstream forms of *T. b. brucei* leading to an accumulation within trypanosomes[25]. Resistance can easily be induced in the laboratory[23,26] and is primarily due to a lack of capacity to import pentamidine.

Pentamidine is highly efficient for cure of first stage HAT (i.e. no trypanosomes in the CSF and a WBC count of < 5 mm^{-3}).

The reported treatment failure rate after a course of five injections is approximately 7%[27,28]. Part of such treatment failures may be explained by second stage infections, which were misdiagnosed as first stage disease, rather than by pentamidine resistant trypanosomes, which have not been described so far in the field. Neujean and Evens[29] reported that 16% of pentamidine-treated patients relapsed but could subsequently be cured with melarsoprol.

3.3 Development of Novel Aromatic Dicationic Analogs of Pentamidine to Treat Microbial Infections

Intensive research has been conducted to develop novel aromatic dicationic compounds as potential new drugs active against multiple AIDS-associated opportunistic pathogens, including *Pneumocystis jiroveci*, *Cryptococcus neoformans*, *Candida albicans* and *Cryptosporidium parvum*[30,31,32,33,34,35,36,37,38,39,40,41]. Criteria for new candidate drugs include increased broad spectrum antimicrobial activity, reduced toxicity and improved pharmacokinetic properties compared to pentamidine. A number of promising dications have been identified with improved efficacy and reduced toxicity in animal models of pneumocystosis and cryptosporidiosis[33,36,37,38,40,41]. A dicationic compound, the diphenylfuran DB75, was selected as the lead compound. DB75 has excellent broad spectrum antimicrobial activity against other opportunistic pathogens when administered intravenously, but it has poor oral bioavailability. Thus, it is orally effective against the gastrointestinal parasite, *C. parvum*, but is not orally active against *P. jiroveci* and other systemic fungal infections in the animal models.

3.4 Oral Activity of the Prodrug DB289 against Experimental African Trypanosomiasis

The diphenylfuran diamidine compound, DB75, was shown to be active when parenterally administered to mice and monkeys infected with the African trypanosome, *T. b. rhodesiense*[42,43,44]. DB75, however, was orally active only when given at very high doses. The markedly enhanced oral anti-*P. jiroveci* activity of its prodrug, DB289, encouraged testing of DB289 as a potential oral agent active against African trypanosomes. Mice were infected with a monomorphic strain of *T. b. brucei* (S427, clone 22) and the infection was allowed to develop for three days before treatment began. All untreated control mice died, with a mean survival time of 5.4 days. Trypanosomes were rapidly cleared from blood of mice when treated with one oral dose (9.5 mg/kg) of

DB289 and all treated mice survived 30 days post treatment with no recrudescence of infection, and were thus considered cured in this acute model of infection. Intravenous injection of one dose (1.9 mg/kg) of DB289 or pentamidine also cured all animals. No overt toxicity was observed in animals treated with DB289 orally or intravenously. Next, an oral dose response experiment was performed. DB289 was given as a single dose orally by gavage over the range of 0.38 to 38 mg/kg three days after infection, and a 50% effective dose (ED_{50}) value calculated from the number of animals considered cured 30 days after treatment. DB289 had an excellent dose response, with an ED_{50} of 2.7 mg/kg. No adverse reactions were observed for any of the oral doses tested.

DB289 was also effective orally in a chronic (i.e., late-stage-like) mouse model of trypanosome infection. Mice were infected with a pleomorphic strain of *T. b. brucei* (GuTat 3.1) and the infection was allowed to develop for 28 days before treatment began. Untreated control animals survived a mean of 41.8 days post infection. Animals were treated orally with DB289 (9.5 mg/kg/day) or intravenously with pentamidine or DB75 (1.5 and 1.7 mg/kg/day, respectively) on Days 28, 29 and 30 post infection. All treatments reduced parasitemia to below detectable limits within 72 hours after treatment. Parasitemia in all animals treated with intravenous pentamidine or DB75 recrudesced, however, within the 30 day post treatment observation period. In contrast, parasitemia in animals treated orally with DB289 remained below limits of microscopic detection throughout the 30 day post treatment period. At the termination of the experiment, tissue extracts (brain, liver, spleen) were prepared and aliquots injected into cyclophosphamide immunosuppressed mice. The recipient mice were examined for a subsequent 30 day period. All mice injected with brain extracts eventually presented with positive parasitemia, indicating oral treatment for 3 days at 9.5 mg/kg/day was not sufficient to completely eliminate trypanosomes from the central nervous system in this model.

DB289 was effective in a vervet monkey model of *T. brucei rhodesiense* infection. In this model, vervet monkeys (3per dose level) were infected with *T. b. rhodesiense* and treated with DB289 beginning on Day 7 of the infection. DB289 was administered orally for five days at dosages of 1, 3 and 10 mg monomaleate salt/kg. These dosages were equivalent to approximately 0.7, 2 and 6.7 mg free base DB289 per kg. Parasitemia were cleared in blood and CSF at all dose levels after the five-day treatment. Recrudescence was not evident until 30-60 days after the start of treatment at the 0.7 and 2 mg/kg dosages. At the 6.7 mg/kg dosage, all three animals remained free of infection in the blood and CSF for at least 180 days.

The data summarized above demonstrate that DB289 potentially represents a significant improvement over the drugs currently used to treat human African trypanosomiasis. Firstly, DB289 is orally available, which would greatly facilitate administration of therapy in the field and make it available not only in specific sleeping sickness centers, but also in selected public health facilities. Secondly, high doses of DB289 have been remarkably well tolerated in animal models of trypanosomiasis and *P. jiroveci*. Thirdly, the prodrug DB289 and its active metabolite, DB75, have increased efficacy compared to pentamidine in animal models of *T. b. rhodesiense* infection. *In vitro* studies indicate that

the IC_{50} for DB75 against *T. b. rhodesiense* is 1.7 ng/ml, and pharmacokinetic studies performed to date in rats and primates indicate that blood and tissue concentrations of DB75 are well above the levels needed for effective antiprotozoal activity can be readily achieved and sustained with oral DB289 administration.

3.5 Safety and Pharmacokinetics of Single Oral Doses of DB289 in Humans

DB289 was administered to healthy male volunteers to evaluate the safety of a single dose and the pharmacokinetics of DB289 after oral administration. This clinical trial was a double-blind, placebo-controlled, sequential dose-escalation study to determine the maximal tolerated dose. At each dose level, a total of 4 subjects received DB289 and 2 subjects received matching placebos. Subjects were monitored for 144 hours post dose and underwent regular follow-up, including ECG and complete hematology, chemistry, and urinalysis testing, and were assessed for treatment-emergent adverse events.

A total of 48 subjects were enrolled in the dose escalation study. The safety of DB289 was evaluated at 25, 50, 100, 200, 400, and 600 mg. The 25 and 600 mg dose levels were repeated because one of the four subjects dosed with DB289 in each treatment group experienced a dose-limiting toxicity.

In this study, one subject experienced a serious adverse event of diverticulitis, considered probably not associated with study drug, and one subject experienced a significant adverse event of ventricular extrasystoles, detected 4 days after the last dose of study drug, considered possibly associated with study drug. All other adverse events were of mild or moderate intensity and resolved spontaneously without treatment. Adverse events noted included headache, muscle ache, abdominal pain, diarrhea, vomiting, sleepiness, drowsiness, nausea, transient fever and epistaxis. There were no clinically relevant laboratory anomalies, including no elevation of liver enzymes. Beside the cardiac rhythm anomaly observed in one subject, there were no abnormal ECG findings. Overall, treatment with DB289 was well tolerated, and the maximally tolerated dose was not identified.

Analysis of DB289 and DB75 levels in the plasma indicate that DB289 was rapidly absorbed after oral intake and metabolized to DB75. There was large inter-individual variability in exposure to DB289 as measured by AUC and C_{max}. In addition to this variability, one subject in the 600 mg group (subject with ventricular extrasystoles already mentioned above) was an outlier, with C_{max} and AUC for DB289 well above the average of the other subjects at this dose group. DB289 showed less than dose proportional increases in C_{max} and AUC. For this reason, dose escalation in this study was interrupted at 600 mg. Thus, the maximum tolerated dose as defined in the protocol was not reached.

To further understand the safety and tolerability of DB289 administered with and without food, a single dose of 300 mg was evaluated in a larger number of subjects. In this phase,

14 male healthy volunteers were treated with DB289 in a double-blind, placebo-controlled, two sequence, balanced, cross-over design, with a 10-day wash-out between each sequence. Eleven subjects received DB289 and 3 received placebo. Subjects received their allocated treatment after a high fat meal in one sequence and on an empty stomach in another sequence.

Two subjects did not complete the study because they were withdrawn for protocol violations unrelated to the study drug. A total of 12 male healthy volunteers completed the trial, 9 treated with DB289, and 3 with matching placebo.

During follow-up, no relevant changes occurred in vital signs, 12-lead ECG, Holter-monitoring or laboratory assessments (including tests for blood in stool). Adverse events were reported by three subjects including one event each of mild headache, epistaxis, chills, vomiting and drowsiness accompanied with moderate fever, which resolved after treatment with 500 mg of paracetamol.

Although there was high variability between subjects, there was an observable increase of both the peak plasma concentration of DB289 and total exposure (as estimated by the area under the plasma concentration time curve) when DB289 was administered after a meal. Although the effect of food on absorption of DB289 was statistically significant, the presence of food did not affect the peak concentration or total exposure to DB75.

3.6 Safety and Pharmacokinetics of Multiple Oral Doses of DB289 in Humans

DB289 was administered to healthy male volunteers to evaluate the safety of DB289 and the pharmacokinetics of DB289 and DB75 after multiple dose oral administration. This was a double-blind, placebo-controlled, sequential dose-escalation study. At each dose level, 8 subjects received DB289 and 4 subjects received matched placebos. Subjects were monitored for 144 hours post-dose and underwent regular follow-up treatment for emergent signs or symptoms of drug exposure including ECG/Holter and complete hematology, chemistry, and urinalysis testing. A total of 36 subjects were enrolled in three dose groups. Based on the results of the single dose and food-effect study, subjects were treated with 25, 50, or 100 mg oral DB289 given twice a day with food for 5 ½ days.

Two subjects were withdrawn from the study for adverse events. One subject in the 25 mg dose group had a bacterial thrombophlebitis of the sampling vein and subcutaneous phlegmon with mild fever and local pain, starting on study Day 4. This adverse event resolved with antibiotic treatment and was assessed by the investigator as not related to DB289. One subject in the 100 mg dose group developed mild erythema on the chest and face. The erythema was considered possibly drug related, and the subject was withdrawn on study Day 5. The event resolved without intervention.

Overall, multiple dose treatment with DB289 was very well tolerated up to the maximum dose of 100 mg twice a day.

3.7 Preliminary Results of Pilot Phase 2a Trial in the Treatment of First Stage HAT

Preliminary, partial and unaudited results are available from this pilot, open label, non-controlled Phase 2a study to evaluate the safety and efficacy of DB289 treatment with first stage *T. b. gambiense* sleeping sickness. Two sites, in Democratic Republic of Congo and Angola, participated in this trial. Subjects were aged 16 years or older with a minimal weight of 45 kilograms and had *T. b. gambiense* present in blood and/or lymph but not in the CSF. Subjects were treated with 100 mg of DB289 orally twice a day for 5 days. Subjects were hospitalized for 144 hours post dose and underwent regular follow-up for treatment-emergent signs and symptoms, including ECG, hematology, chemistry and urinalysis testing.

A total of 32 subjects were enrolled and treated in this trial. Two subjects were withdrawn from the study for adverse events, one patient for high blood pressure and one for persistent high fever. Both adverse events were classified as probably not associated with the study drug. One patient was withdrawn from treatment due to administrative reasons. Beside these two adverse events, clinical tolerance was excellent, and the other reported adverse events were headache, intermittent fever and pruritus.

Clinical laboratory exams indicated a mild (< twice upper limit of normal) increase in liver enzymes (AST and/or ALT) after DB289 treatment compared to baseline in four subjects.

The parasite was eradicated from blood and lymph in 27/29 subjects at testing on Study Day 7 (2 days after DB289 treatment completion). During the 12 month follow-up period, four subjects experienced a relapse (one each at 6 and 8 months post treatment, and 2 at 17 months post treatment). Fifteen of 23 eligible subjects returned for the 24 month follow-up evaluation and one additional relapse has been reported 30 months post treatment.

Pharmacokinetic Analysis

The protocol specified the drawing of 12 blood samples per patient for the analysis of plasma concentrations for the parent drug, DB289, and its active metabolite, DB75. The subjects received ten doses of DB289 on a 100 mg BID schedule, with five of the blood samples being collected immediately predose to doses #1, #4, #6, #8 and #10. The remaining seven samples were collected sequentially following the final dose (#10) so as to describe the steady state pharmacokinetics and the elimination rates of DB289 and DB75. These samples were collected 1, 2, 4, 8, 24, 72 and 144 hours after administration of dose #10. The pharmacokinetic data from this trial are summarized in Table 3.7.a below.

Table 3.7.a Mean (±SEM) Pharmacokinetic Parameters of DB289 and DB75

Parameter	Units	DB289	DB75
		Mean ± SEM	Mean ± SEM
C_{max}	ng/mL	9.44 ± 1.97	26.4 ± 4.1
T_{max}	hr	2.86 ± 0.35	4.17 ± 0.55
C_{min} (predose)	ng/mL	0.899 ± 0.182	13.1 ± 1.5
K_{el}	1/hr	0.175 ± 0.020	0.0136 ± 0.0006
$T_{1/2}$	hr	5.03 ± 0.49	53.1 ± 2.2
$AUC_{(0-12)}$	ng•hr/mL	38.7 ± 5.3	230 ± 27
$AUC_{(0-8)}$	ng•hr/mL	51.2 ± 7.2	692 ± 55
Cl/F	L/hr	3722 ± 598	471 ± 51
Accumulation Factor	ratio	1.31 ± 0.06	3.35 ± 0.19
Metabolite Ratio	mole/mole	-- ± --	9.15 ± 1.40

The pharmacokinetic data in subjects with first stage HAT demonstrated large between-patient variability and rapid attainment of steady-state. The active/prodrug ratio has a wide range and the terminal phase involves a small fraction of drug; simple prospective simulation of DB75 concentrations in the range of 100 mg BID was confirmed by clinical data from subjects in this trial. Pharmacokinetic parameters for male and female subjects were similar, and body size appears to have little impact on DB75 exposure.

The efficacy data at the primary endpoint (End of Treatment), excellent safety profile, and pharmacokinetic profile of DB289/DB75 in patients were considered adequate and appropriate to pursue the next study. No significant safety concerns were identified and the cure rate at the end of treatment was 93% (24 hours post last dose of study drug; primary efficacy endpoint of this study). The plasma profile of DB75 was adequate to treat parasites in this compartment.

3.8 Preliminary Results of Comparative Phase 2b Trial in the Treatment of First Stage HAT

Preliminary, partial and unaudited results are available from an ongoing, open label, randomized and controlled Phase 2b study to evaluate the safety and efficacy of DB289 treatment with first stage *T. b. gambiense* sleeping sickness. Two sites in Democratic Republic of Congo participated in this trial. Subjects were aged 15 years or older with a minimal weight of 35 kilograms and had *T. b. gambiense* present in blood and/or lymph but not in the CSF. Subjects were randomized to treatment with either 100 mg of DB289 orally twice a day for 5 days or a 4 mg/kg pentamidine intramuscular injection daily for 7 days. Subjects remained under daily observation through Day 7 and underwent regular

follow-up for treatment-emergent signs and symptoms, including ECG and hematology, biochemistry and urinalysis testing.

A total of 81 subjects were enrolled and treated in the first phase of this trial. One patient died in the hospital after treatment, before reaching the first follow up, from a probable (undetected) second stage disease with rapid progression and death due to arsobal encephalopathy (DB289 treatment group). One patient died subsequent to the 3 month follow-up assessment from other causes probably not related to trypanosomiasis (pentamidine treatment group). Clinical tolerance of DB289 was good; adverse events and laboratory abnormalities were less frequent and less severe for DB289-treated subjects than for subjects treated with intramuscular pentamidine.

In 35/40 subjects treated with DB289, the parasite was eradicated from blood/lymph upon testing at Study Day 7 (24 hours after DB289 treatment completion). The 41 subjects treated with pentamidine demonstrated eradication at the End of treatment.

Subjects are under follow-up, and > 80% follow-up was achieved at the 3 and 6 month evaluations. Sixty of 76 subjects completed the three-month follow up and 68 of 74 eligible subjects attended the 6 month follow up; however, 7 subjects refused lumbar puncture at this evaluation. Of the 71 eligible subjects, 54 have completed the 12 month follow up evaluation; however, 11 declined to undergo lumbar puncture. No relapses in the DB289 treatment group and one suspected relapse in the pentamidine group have been reported to date.

Enrollment in the randomized portion of the trial was discontinued after enrollment of 81 subjects, as 5 subjects in the DB289 treatment group experienced treatment failure at the End of Treatment evaluation. The protocol was amended to continue to enroll subjects into an open-label treatment with DB289 100 mg BID for 10 days (Section 2 of the Phase 2b trial). Thirty subjects were enrolled in this portion of the trial. Although 2 subjects still had evidence of infection at Day 7 of treatment, all were negative for parasites at Day 12 (24 hours post treatment), and no relapses have been reported to date. At the 3 month follow up (primary efficacy endpoint for this trial), as of December 15, 2004, 15 of 20 eligible subjects have been evaluated, with two subjects declining to undergo lumbar puncture. Subjects will continue to be followed through the 24 month time point. No serious adverse events were reported in this portion of the trial, with DB289 administered as 100 mg BID for 10 days.

DB289 administered for either 5 days or 10 days was well tolerated. Most subjects reported adverse events (83% of 10-day DB289, 50% of 5-day DB289, and 95% of pentamidine treatment groups for any adverse event). The most commonly occurring adverse events for the 3 treatment groups were vertigo, nausea, vomiting, injection site pain and injection site reaction (pentamidine group only), pyrexia, increased AST and ALT, headache and hypotension. All AST and ALT elevations in subjects treated with DB289 were characterized as mild, while 15/41 subjects in the pentamidine treatment

group experienced Grade 3 liver enzyme elevation, and one patient was reported to have Grade 3 hypertension during treatment.

Table 3.8a (below) summarizes the efficacy data from the Phase 2 program for first stage HAT. DB289 was administered as 100 mg BID in all Phase 2 studies.

Table 3.8.a Efficacy in Phase 2 Trypanosomiasis Trials (as of December 2004)

	Phase 2a	Phase 2b		Phase 2b-2
	**DB289 5 days**	**DB289 5 days**	**Pentamidine 7 days**	**DB289 10 days**
Treatment Completed	29	40	41	30
Treatment Failures	2	5	0	0
Relapses	5	0	0	0
Fatalities during Follow-up	0	1	1	0
Cure Rate	76%	85%	98%	100%

The efficacy data at the primary endpoint (three months post treatment) and excellent safety profile in subjects were considered adequate and appropriate to pursue the Phase 3 pivotal program. No significant safety concerns were identified, and DB289 was significantly better tolerated than pentamidine (full details of the safety profile are presented below). The Phase 2a and Phase 2b-1 trials established the minimum effective dose to be = 100 mg BID x 5 days. To date there have been no treatment failures and no relapses in the subjects treated with DB289 100 mg BID for 10 days.

3.9 Overview of the Safety and Tolerability of DB289

Ongoing safety evaluations in the clinical trials include assessment of individual serious and significant (including severe) adverse events, overall incidence and severity of adverse events, and laboratory assessments. Specific criteria for serious and significant adverse events include one or more of the following: meet criteria for a serious adverse event, as defined by Good Clinical Practice (International Conference on Harmonization and US Code of Federal Regulations); lead to withdrawal of study drug; categorized as severity of Grade III or higher; unexpected (i.e., not previously reported in studies of DB289); and associated with use of study drug. Overall incidence of adverse events is evaluated across all studies and across doses (assessment of potential dose correlation). Laboratory tests are analyzed for change from baseline and for reversibility.

3.9.1 Serious and Significant Adverse Events

Two subjects have died while participating in DB289 trials; both were participating in trials of first stage HAT. One patient died in the hospital after treatment, before reaching the first follow up from a probably undetected second stage disease with rapid progression and death due to arsobal encephalopathy (DB289 treatment group). A second subject (pentamidine treatment group) died 3 months after completion of treatment for trypanosomiasis from unknown causes, probably unrelated to trypanosomiasis, and possibly related to complications of lumbar puncture.

Seven subjects have experienced other serious adverse events during or subsequent to treatment with DB289. In Phase 1 healthy volunteer studies, events of diverticulitis (unlikely associated with study drug), and acute hepatitis with onset of symptoms 11 days after last dose of study drug (possibly associated with study drug) were reported. In the Phase 2 trypanosomiasis trials, 1 subject treated with DB289 for 5 days experienced pyrexia difficult to control with antipyretics (unlikely associated with study drug). Four subjects in the Pneumocystis Phase 2 trial experienced serious adverse events; progression of underlying renal insufficiency (possibly related to study drug), recurrent fever (unlikely association to study drug), leg pain with suspected peripheral neuropathy (possibly related to study drug), and axonal polyradiculopathy in a patient with history of recurrent cryptococcal central nervous system infection (unlikely association to study drug). The events of leg pain with suspected peripheral neuropathy and axonal polyradiculopathy occurred subsequent to the 21 day treatment period.

Based on these initial reports of significant adverse events, body systems have been identified for ongoing surveillance for potential drug toxicity. These include the liver, neurological and/or muscular systems, and the cardiovascular system.

3.9.2 Incidence and Severity of Individual Adverse Events

In clinical trials conducted to date, DB289 has been well tolerated. A few subjects have experienced mild to moderate elevations of AST and ALT. Observations of elevations of liver enzymes in HAT subjects were concurrent with treatment, resolved spontaneously (within 2-4 days), and were asymptomatic. There was no evidence for increased adverse events with longer duration of therapy; adverse events for 5 day and 10 day treatment with DB289 were similar. As the liver was identified as a target organ for toxicity in the preclinical animal studies, significant focus is placed on assessing potential toxicity in humans. At this time, liver enzyme elevations have not been observed in all trials, including those with higher drug accumulation.

Table 3.9.2.b below delineates the frequency of adverse events (all adverse events without regard to association to study drug) that were reported in the Phase 2b trial of DB289 for treatment of trypanosomiasis.

Table 3.9.2.b Adverse Events reported in Phase 2b clinical trials of first stage HAT, without regard to association to study drug

Adverse Event	DB289 10 days n=32	DB289 5 days n=40	Pentamidine 7 days n=41
Abdominal Pain	0%	0%	5%
ALT	3%	3%	68%
AST	3%	13%	83%
Asthenia	7%	3%	0%
Blood Creatinine	0%	3%	4%
Cough	3%	3%	0%
Diarrhea	0%	3%	5%
Enteritis	0%	3%	0%
Headache	57%	25%	24%
Hemoglobin Decreased	0%	3%	0%
Hypertension	0%	0%	2%
Hypoglycemia	0%	0%	2%
Hypotension	17%	5%	12%
Injection Site Pain	0%	0%	20%
Injection Site Reaction	0%	0%	10%
Nausea	13%	3%	10%
Neck Pain	0%	0%	5%
Prothrombin Time Increased	0%	3%	0%
Pyrexia	20%	10%	20%
Vertigo	17%	13%	15%
Vomiting	10%	3%	7%

3.9.3 Electrocardiographic Findings

ECG analyses of data collected in the Phase 2a and 2b studies demonstrated no life threatening cardiac adverse event; there was no pathologic QT prolongation under treatment. T wave changes before and under treatment were considered to be compatible with a HAT myocarditis and the inflammatory response to treatment of HAT, a cardiotoxic effect of DB289 is less likely. One patient was noted to have AV bloc II

during therapy, which was not noted in the pretreatment ECG. One other subject was noted to have AV block I. The relationship to study drug needs further evaluation, as cardiac effects of trypanosomiasis may potentially cause AV block. However, the available literature on this topic is very limited[45,46,47,48,49,50,51,52].

3.9.4 Pregnancies in First Stage HAT Trials

Two pregnancies have been reported in female subjects undergoing DB289 treatment. The first occurred in the Phase 2a trial. The patient was found to have a positive pregnancy test after treatment; thus, treatment occurred in first trimester. The child was reported to be normal after birth, and continues to be followed with the mother at regular evaluations. The second patient was found to have a positive pregnancy test during the 10 day treatment with DB289 (first trimester exposure). The pregnancy is progressing normally, and the delivery is due in February 2005.

3.9.5 Reproductive Studies in Animals and Relevance to Treatment of Pregnant and Lactating Women

Reproductive studies of DB289 in animals have not indicated any embryo or fetal toxicity, or other effects on reproductive function of adult male and female rats or rabbits. In the CD rat, none of the studies indicated any selective adverse effects of DB289 on reproductive parameters. Litter survival and postnatal growth gave no cause for concern. Functional development and sexual performance of offspring did not indicate any latent effect of maternal treatment but it was outside the scope of these studies to evaluate any direct effects of DB289 on the juvenile population. In the rabbit, there was no evidence of any significant impairment of embryo-fetal growth or development in surviving litters.

The milk from CD rats was tested for the presence of DB289 and metabolites, including DB75. Rats were administered a single oral dose of ^{14}C-DB289 at 10 mg/kg on Day 12 post-partum. Plasma and milk were collected for measurement of radioactivity and quantification of DB289 and DB75 at multiple time points during the 24 hour period after dosing. Radioactivity was detected in milk during the 0.5 to 6 hour period after dosing. The milk to plasma AUC_{1-24h} ratio for radioactivity was 8:1. The major component in milk at early times after dosing was DB289, and the estimated dose to pups was 20 μg equivalent DB289, about 0.5% of the maternal dose. The major component in milk at later times was DB810 (ratio of DB810 to DB289 was 9:1). Thus, suckling neonates were exposed to low levels of DB289 and its metabolite in milk; no significant level of DB75 was detected.

Therefore, it is appropriate to proceed with studies of pregnant and lactating women. Enrolling these subjects in this Phase 3 trial will provide further safety and efficacy data for both DB289 and pentamidine in these special populations.

4.0 Objectives

4.1 Primary Objective

The primary objective of this study is to compare the efficacy, safety and tolerability of oral DB289 versus intramuscular pentamidine, for treatment of first stage HAT caused by *T. b. gambiense*.

4.2 Secondary Objective

In a substudy of pregnant or lactating female subjects, to compare the efficacy, safety and tolerability of DB289 versus pentamidine for treatment of first stage HAT, and to assess the pharmacokinetic profile of DB289 and DB75 in plasma and breast milk in this population.

5.0 Investigational Plan

5.1 Overall Study Design

This is a multi-center, multi-country, open label (sponsor blinded), parallel group, comparator controlled, randomized Phase 3 trial. Subjects who are = 12 years of age with first stage HAT caused by *T. b. gambiense* will be randomized to receive either 100 mg of DB289 orally twice a day for 10 days or a 4 mg/kg pentamidine intramuscular injection daily for 7 days.

A total of 250 subjects who satisfy the inclusion and exclusion criteria will be enrolled in this trial in order to obtain approximately 200 clinically evaluable subjects in the Per Protocol population at the primary endpoint (12 months post treatment). Enrollment is planned to begin third quarter 2005 and to be completed in approximately 12 months and the last follow up evaluation is expected to be completed approximately 36 months after the initiation of enrollment. Each patient is expected to participate for 24 months.

Five to seven sites will be selected in the Democratic Republic of Congo, Angola, and South Sudan. Additional countries may also be considered, if appropriate sites with adequate numbers of subjects are identified. Selection of sites in some of these countries will depend on the security situation at the time of study initiation.

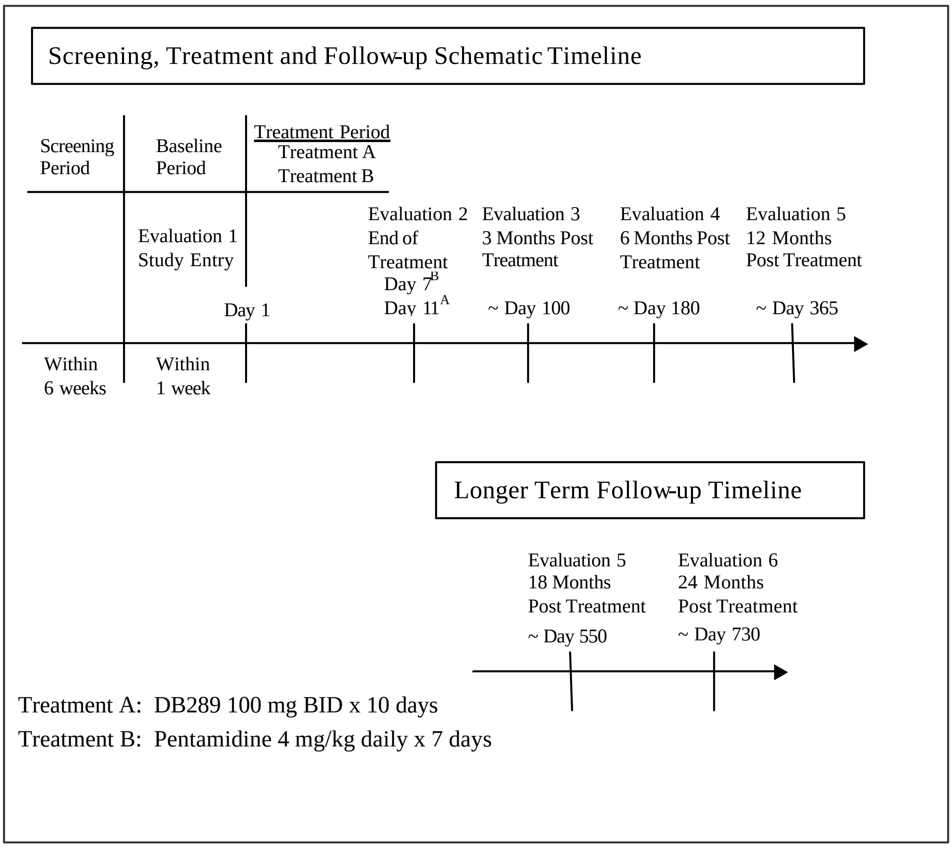

Figure 5.1.a Study Design Schematic

All subjects will undergo screening to demonstrate the presence of trypanosomes in the blood and/or lymph, and exclude evidence for second stage disease (no trypanosomes detected in the CSF and = 5 WBC per mm^3 in CSF). Subjects will undergo safety evaluations including vital signs, physical examination, adverse event monitoring, and collection of concomitant medications throughout the treatment period and at the End of Treatment evaluation. Subjects will also undergo laboratory testing for chemistry (AST, ALT, total bilirubin, glucose, creatinine), hemoglobin, and ECG monitoring to the extent possible at each site at Baseline and End of Treatment evaluations.

Children ages 12 to 15 years and weighing = 30 kg can be enrolled at sites with adequate facilities to perform laboratory testing for chemistry, hemoglobin and ECGs (i.e., sites with adequate electrical power). Subjects ages 12 -15 will also undergo safety laboratory testing at the 3 month post treatment visit.

Pregnant and lactating female subjects may be enrolled in this trial with the understanding that they will be asked to participate in additional safety measurements. These subjects may be enrolled at sites capable of collecting and processing samples for pharmacokinetic analysis and laboratory testing for chemistry, hemoglobin and ECGs

(i.e., sites with adequate electrical power). Pregnant and lactating female subjects will undergo blood sampling and lactating female subjects will also undergo milk sampling in order to assess the pharmacokinetic profiles of DB289 and DB75 in these body fluids.

Efficacy will be evaluated at End of Treatment and at 3, 6, 12 and 18 months post treatment in the blood and lymph for the absence of trypanos omes. Lumbar puncture to rule out second stage disease will be performed pretreatment and at 6, 12 and 18 months post treatment, and at any other evaluation where relapse is suspected or demonstrated in blood or lymph nodes. Additional assessments of clinical efficacy will be performed at 24 months post treatment. The 12 month post treatment evaluation will be defined as the Test of Cure evaluation, based on Draft Informal Consultation from WHO (9Sep2004).

At any time during the study, the patient or investigator may elect to discontinue a patient's participation in the study.

5.1.1 Discussion of Study Design and Choice of Control Groups

This Phase 3, open label, comparator controlled, parallel group, randomized, multi-center, international study is designed to compare the efficacy and safety of DB289 100 mg BID for 10 days and pentamidine 4 mg/kg per day for 7 days in subjects with first stage HAT. The card agglutination trypanosomal test[53] (CATT) for *T. b. gambiense* will be used for screening subjects. Diagnosis of trypanosomiasis and parasitological response to therapy will be assessed by the use of the following tests: microscopic examination of blood (thin and/or thick smear), hematocrit centrifugation of blood (WOO)[54], microscopic examination of lymph node aspirate, microscopic examination of blood after m-AECT concentration[55], and WBC and microscopic examination of CSF fluid.

Study efficacy parameters and timing of post treatment evaluations are based on WHO Draft Informal Consultation on the Conduct of Clinical Trials in Human African Trypanosomiasis (9Sep2004).

Two types of clinical sites will enroll subjects in this trial. Some sites are equipped with ECG and sufficient clinical laboratory (e.g., CDTC Maluku, CDTC Bandundu, Evangelic Hospital of Vanga, CDTC Uíge, Malteser Hospital Yei). Other sites have only very rudimentary laboratory facilities (e.g., Evangelic Hospital of Kikongo).

Although 18 or 24 months post treatment is preferred to assess clinical cure in HAT control programs, the drop out rate increases significantly after 6-12 months; approximately 40-80% of subjects will be lost to follow up by 18 months post treatment. Therefore, the Per Protocol data set at the 12 month evaluation was chosen as the primary endpoint, in order to maintain a robust dataset for the analyses. Secondary analyses will be performed, using appropriate measures to handle drop outs, on all data sets, including the 18 and 24 month post treatment assessments.

The study will be conducted as an open label design, as DB289 is administered orally, while pentamidine is administered intramuscularly. The primary efficacy variable for the trial is objective, i.e., absence or presence of trypanosomes in blood, lymph nodes or CSF. Therefore, blinding would not be necessary to prevent bias in the assessment of efficacy. In addition, a double blind study would cause significant hardship to the patients and sites conducting this trial. However, the sponsor (Immtech International, Inc.) will be blinded to the randomization assignment.

Pentamidine administered via the intramuscular route is the only recognized therapy for first stage HAT caused by *T. b. gambiense* and is the appropriate control for this trial.

Generally, the safety and efficacy of a new drug should be established in a trial population representative of the target population. In the case of *T. b. gambiense* infection, the target population does include a significant proportion of pregnant women, lactating women and children of all ages; currently, they are treated with pentamidine i.m., which has not been well-studied in these patient populations[56,57,58]. For all these patients, treatment with an effective drug is required to prevent the potential for progression to the second stage of African trypanosomiasis.

In the course of normal clinical practice in the countries where *T. b. gambiense* is endemic, testing for pregnancy by urine or blood test is not available, and therefore, detection of early pregnancy would be dependent on menses history and physical exam. This makes even more critical the study of this patient population in the scope of a controlled clinical trial, as these patients are likely to receive treatment with DB289 following licensure.

Reproductive studies of DB289 in animals have not indicated any embryo or fetal toxicity, or other effects on reproductive function of adult male and female rats or rabbits. Therefore, it is appropriate to proceed with studies of pregnant and lactating women. Enrolling these subjects in the Phase 3 trial will provide further safety and efficacy data for both DB289 and pentamidine. Thus, this Phase 3 trial will include pregnant and lactating female subjects and children = 12 years of age. Younger children will be studied in separate protocols.

No DB289 dosing adjustment is expected to be needed for treatment of pregnant women, regardless of trimester of pregnancy. Current data on the pharmacokinetics of DB289 and DB75 have identified some parameters, such as food intake, that impact DB289 concentrations but do not affect circulating levels of DB75. Observed plasma concentrations of DB289 and DB75 have shown a wide range of variability, with no apparent relationship to body weight or body size. The additional factor of progression of pregnancy is not expected to result in added variability beyond that currently observed. Subjects will be monitored for clinical outcome and pharmacokinetic values on an ongoing basis, and if indicated, dosing in this population may be amended.

Subjects older than 65 years may be enrolled in this Phase 3 pivotal trial, and no upper age limit has been defined.

5.2 Study Population

Both male and female subjects 12 years of age or older, who meet all of the inclusion criteria listed in Section 5.2.1 and exhibit none of the exclusion criteria listed in Section 5.2.2 of this protocol, will be eligible for enrollment.

5.2.1 Criteria for Inclusion

1. The patient has first stage *T. b. gambiense* infection; i.e., parasitologically confirmed infection in the blood or lymph node aspirate and = 5 WBC mm^{-3} detected in the CSF by microscopic examination.

2. Patient is male or female = 12 years of age and = 30 kg.

3. Patient has understood and signed the Informed Consent. If the patient is minor or mentally impaired, a legal guardian has also signed the Informed Consent.

5.2.2 Criteria for Exclusion

1. The patient has possible or confirmed second stage *T. b. gambiense* infection; i.e., presence of parasite in the CSF upon microscopic examination or a WBC count in the CSF of > 5 mm^{-3}.

2. Active clinically relevant medical conditions that in the Investigator opinion may jeopardize subject safety or interfere with participation in the study, including but not limited to: significant liver diseases, chronic pulmonary diseases, significant cardiovascular diseases, diabetes, thyroid diseases, gout, infection including known HIV infection, CNS trauma or seizure disorders (A list of typical signs and symptoms is provided for guidance of the investigator in Appendix 1).

3. Coma Score of less than 9 on the Glasgow Coma Scale (Appendix 2).

4. Any condition which compromises ability to communicate with the investigator as required for the completion of this study.

5. The subject has been previously treated for HAT.

6. The subject has been previously enrolled in the study.

5.2.3 Criteria for Discontinuation

5.2.3.1 Discontinuation of Individual Subjects

A subject can be discontinued from the study for the following reasons:

1. Withdraws voluntarily from the study

2. Lost to follow-up

3. Dose limiting toxicity, defined as an adverse event or biological anomaly at least possibly related to treatment with a severity Grade 3 or higher on the toxicity scale (see Appendix 3) or graded as severe or intolerable by the Investigator.

4. At the discretion of the Principal Investigator for the safety of the subject or to maintain the integrity of the trial.

5. At the discretion of the Principal Investigator if the patient is not compliant to the requirements of the protocol.

If, for any reason, a subject is discontinued from the study before the End of Treatment evaluations (Day 7 for the pentamidine group and Day 11 for the DB289 group), the Investigator is required, as feasible, to perform the safety procedures planned for the End of Treatment.

Subjects in the DB289 group who have to be withdrawn from the trial prior to completion of the treatment will be advised and offered a standard course of pentamidine or other appropriate treatment.

If a subject develops an adverse event of Grade 2 or higher, he/she will remain under observation until the adverse event is resolved, stabilized, or is otherwise explained.

Discontinued subjects will not be replaced.

5.2.3.2 Discontinuation of the Entire Study

Immtech International, Inc. may terminate this study prematurely, either in its entirety or at this site, for reasonable cause provided that written notice is submitted a reasonable time in advance of the intended termination. The Investigator may also terminate the study at their site for reasonable cause, after providing written notice to the Sponsor a reasonable time in advance of the intended termination. Neither party requires advance notice if the study is stopped due to safety concerns. If Immtech International, Inc. chooses to terminate the study for safety reasons, it will immediately notify the investigator and subsequently provide written instructions for study termination.

5.2.3.3 Scientific Advisory Board

The study will be supervised by a scientific advisory board appointed and chaired by Dr. Fred Sparling, Professor of Medicine and Infectious Diseases, UNC, Chapel Hill, USA.

5.2.3.4 Data and Safety Monitoring Board

A formal Data and Safety Monitoring Board (DSMB) will be established to oversee the trial. The DSMB will have a charter and formal stopping rules for the trial. The DSMB will evaluate the data at the interim analysis and provide recommendations to the sponsor with regard to continuation of the trial.

5.3 Treatments

5.3.1 Treatments Administered

Treatments will be administered according to the following schedule.

	Dose	Regimen	Duration
DB289	100 mg<br>(1 tablet of 100mg)	Twice a day (morning and evening)	10 Days<br>(20 doses)
Pentamidine	4 mg/kg<br>for injection	Once a day (morning)	7 Days

Pentamidine will be administered according to the procedure routinely used by the National program or the responsible agency for the respective treatment center.

5.3.2 Identification and Handling of Investigational Product

5.3.2.1 Identity of Investigational Product

Immtech International, Inc. will provide DB289 for the study.

	Presentation	Dose / Tablets	Tablets / Bottle	Storage
DB289	Tablets	100 mg	50	Ambient

Pentamidine will be obtained locally by the agency responsible for each center as pentamidine isethionate (Aventis) for injection in single dose vials at 200 mg per vial.

5.3.2.2 Packaging and Labeling

Each bottle of DB289 will be labeled with a single-panel, computer generated label that will include at minimum the following information:

- Sponsor identification
- Protocol number
- Required cautionary statements
- Drug identification, including lot number
- Storage conditions
- Dosing instructions

5.3.2.3 Storage and Disposition

Investigational products are for investigational use only and the study drug supplied for this study is intended for use only within the context of this study. The study drug supplied for this study should also be stored in a secure place and maintained under adequate security until dispensed for subject use or returned to Immtech International, Inc.

DB289 tablets should be stored at < 30°C (86°F); limited excursions to 15°- 35°C (59°-95°F), are permitted. Excursions up to 40°C are permitted as long as they do not exceed 24 hours.

5.3.2.4 Drug Accountability

The investigator, or their designee, will verify that study drug supplies are received intact and in the correct amounts by signing and dating the Drug Inventory Log (includes: Lot number, expiry date, number of bottles received and dispensed). The sites will also maintain patient specific drug dispensing logs.

An overall accountability of study drug will be performed and verified throughout the study and at the site closeout visit. All used and unused study drug supplies will be inventoried, accounted, and be destroyed by the CRO or returned to Immtech International, Inc. at the end of the study. By signing the Investigator Agreement page of this protocol, the investigator agrees not to supply study drug to any person(s) not enrolled in the study or named as a sub-investigator.

5.3.3 Method of Assigning Subjects to Treatment Groups

The subjects will be randomized in blocks of variable size in the order in which they are enrolled according to a randomization schedule prepared by Immtech International, Inc.

Each study site will be provided with series of individual envelopes each containing a card with the treatment assignment for one patient and a control number. After a patient has signed the informed consent and inclusion/exclusion criteria have been confirmed,

the Investigator will open the next envelope in the randomization list to obtain treatment assignment for that patient. The Investigator will then transfer the control number to the patient's CRF.

If a randomized patient does not receive treatment for any reason, his/her randomization number will not be reused.

5.3.4 Selection of Doses and Timing of Dose

5.3.4.1 Support for DB289 Dose and Dosing Frequency

The pharmacokinetics of DB289 and DB75 were evaluated in healthy male volunteers. Most of the subjects were Caucasians. Administration of 100 mg of DB289 twice a day resulted in sustained DB75 plasma levels above the target anti-trypanosomal concentration known to be effective *in vitro* and *in vivo*. Doses of 50 mg and higher resulted in sustained blood levels in the range of the plasma concentrations that were effective in animal experiments. In the single dose study, administration of doses higher than 100 mg DB289 did not result in a substantial increase in the plasma AUC for DB289, which is indicative of saturation of absorption and/or metabolites formation. The twice-daily regimen resulted in moderate accumulation of DB75. In the multiple dose study, 100 mg DB289 given twice a day was very well tolerated.

Pharmacokinetic sampling in the Phase 2a trial in first stage HAT demonstrated that the terminal half-life of DB289 was approximately 5 hours, consistent with twice daily dosing.

Administration of DB289 with food (standard high fat meal) resulted in an approximate two-fold increase in DB289 mean plasma concentrations in healthy male volunteers. However, the increase of DB75 was less and did not reach statistical significance. Although important for the understanding of the pharmacology of DB289, this finding has little clinical significance. High proportions of subjects with sleeping sickness in Africa are malnourished and usually have only one meal a day with low fat content. In the first trial of DB289 in *T. b. gambiense* sleeping sickness, DB289 was given twice a day with a glass of reconstituted powder milk. Even this simple measure proved difficult to implement at the sites in the prior trials; requiring specific restrictions for dosing relative to meals will become impractical in rural areas once the drug is licensed for use.

These data and the expected utilization patterns support the choice of a 100 mg dose given twice daily without restrictions to timing with respect to meals.

5.3.4.2 Support for Duration of Dosing

Experimental results in monkey models of trypanosomiasis indicated that DB289 effectively clears the parasites from the blood of infected animals with treatment duration of 3-7 days. This duration of treatment is consistent with the established efficacy of pentamidine against *T. b. gambiense* when administered for 7 to 10 days.

In the Phase 2 trials, DB289 has been administered to subjects at 100 mg twice a day for 5 days. Two treatment failures in the Phase 2a study in one site in Angola and five treatment failures were observed at one site in Democratic Republic of Congo in the Phase 2b trial, with detection of the parasite only in the lymphatic system. This experience leads to the hypothesis that the extent of drug exposure is within the efficacy range in most but not all subjects, and that the failure rate might be reduced further if exposure to DB75 was increased.

Data obtained *in vitro* with *T. b. brucei* indicate that parasite elimination is dependent on the duration of exposure to DB75, rather than increasing concentrations of drug. Also the uniformity of the pharmacokinetics over time, and the rapidity with which DB75 concentrations approach steady-state, all support the option for increasing the duration of treatment from 5 to 10 days to maximize anti-trypanosomal activity.

Longer exposure to DB75 is supported by the excellent safety and tolerability observed to date with the current dose of 100 mg DB289 BID for 5 days or 10 days. No increase in frequency or severity of adverse events was noted for the 10 day treatment, compared to the 5 day duration of therapy. Moreover, preliminary safety information for the treatment duration of 21 days from a trial of DB289 in *P. jiroveci* pneumonia (PCP) in AIDS subjects indicates that a longer duration of therapy with DB289 is well tolerated.

These data support the duration of treatment of 10 days in this Phase 3 trial of first stage HAT.

5.3.5 Blinding

No blinding of study drug will be performed in this study at the investigation sites or the CRO responsible for the monitoring of the trial. However, the sponsor (Immtech International, Inc.) will be blinded to the randomization assignment.

5.3.6 Prior and Concomitant Therapy

Any medication the subject has received within 7 days prior to enrollment, is receiving at the time of enrollment or receives during the study between the first dose of study drug and the End of Treatment evaluation at Day 12 will be recorded in the subject's medical record and on the appropriate CRF, along with the reason for use, dates of administration, and dosages.

5.3.7 Treatment Compliance

Treatment compliance will be guaranteed by the direct administration of the drugs exclusively by the assigned study center staff.

5.4 Study Plan

5.4.1 Efficacy and Safety Measurements Assessed and Flow Chart

Refer to Appendix 11 for the Study Flow Chart.

5.4.1.1 Pretreatment Period

5.4.1.1.1 Screening Evaluation

The subjects will be recruited among the patients reporting to the Trypanosomiasis Treatment Centers or being detected during mobile diagnostic campaigns and referred to the study centers for treatment. Patients diagnosed at the centers with first stage African Trypanosomiasis will be considered candidates for this trial.

The following examination will be performed during screening:

- CATT[53] test for *T. b. gambiense*
- Microscopic examination (thin and/or thick smear) of blood and lymph node aspirate for trypanosomes

Although the screening information will be collected on the CRF, it will be considered as historical pre-study information and it will be repeated independently during the baseline examination at the center.

Subjects will be screened within 7 weeks prior to dosing with DB289 or pentamidine (within 6 weeks prior to the baseline evaluation).

5.4.1.1.2 Baseline Evaluation

The following tests and procedures are to be performed:

1. Demographic information with height, weight and body mass index (calculated).

2. Complete medical history. For all women, the fertility status will be noted (parity, childbirth within 12 months, menopause).

3. Signs and symptoms typical of HAT will be queried and evaluated, if present, according to the HAT symptom grading scale outlined in Appendix 12[59]: lymphadenopathy, temperature, headache, pruritus, daytime sleep, nighttime sleep, tremor, speech impairment, abnormal movements, walking disability, general motor weakness, unusual behavior, inactivity, aggressive behavior, and disturbance of appetite.

4. Concomitant diseases and pretreatment medications taken for the 7 day period prior to dosing.

5. Vital signs including blood pressure, heart rate, respiratory rate and temperature, taken in the sitting position.

6. Physical examination and Glasgow Coma Scale.

7. Confirmatory tests for presence of trypanosomes in blood and/or lymph, and lumbar puncture to exclude presence of second stage HAT, as outlined below.

The following battery of tests will be performed in the order indicated to confirm the diagnosis of the screening examination within a maximum of one week prior to treatment initiation. Tests must be performed in the order as indicated below; if a test is found to be positive for trypanosomes, subsequent tests need not be performed:

- Microscopic examination of blood (thin and/or thick smear)
- Hematocrit single and double centrifugation of blood (WOO)[54]
- Microscopic examination of lymph node aspirate
- Microscopic examination of blood after m-AECT[55] concentration

When a lymph node aspirate is not feasible (for example absence of adenopathy or node impossible to puncture) this will be noted. Five ml of whole blood will be collected for parasite strain genotyping by DNA analysis in order to attempt the distinction between relapses and reinfection in case of recurrence of the parasite. The method is experimental and not yet validated.

A sample of CSF will be obtained by lumbar puncture and the following tests will be performed:

- The WBC in the CSF will be counted.
- Microscopic examination of CSF for trypanosomes will be performed (if possible, the modified single centrifugation technique[60] should be done).
- Latex IgM agglutination test[61] will be done. The method is experimental and not yet validated.

The patients, who qualify for the study after the above standard procedures have been performed, will be asked to provide written informed consent prior to undergoing any study specific procedures or treatment.

The subjects enrolled at a site properly equipped will undergo the following exams within 72 hours prior to dosing:

- Hematology: hemoglobin
- Chemistry profile: serum glucose, creatinine, AST, ALT, total bilirubin
- ECG

For women of child bearing potential, a urine pregnancy test will be done before treatment.

Eligibility will be determined by the inclusion/exclusion criteria. Subjects may be admitted as inpatients to the clinical site for the treatment period. Those who are able to come back on a daily (pentamidine) or twice daily (DB289) basis will be admitted as outpatients. However, all subjects qualifying to be treated as outpatients will be instructed to stay within reach of the clinical research facility during the 11 days treatment/observation period so they can report for each scheduled evaluation for treatment and observations.

Diagnosis and treatment of concomitant diseases such as malaria, diarrhea or filaria will be done according to current guidelines at the site before initiation of treatment with DB289 or pentamidine. Treatment of filariasis will be postponed until completion of the application of trypanocidal drugs. Malaria will be tested on thin and/or thick smear of blood. Any diagnoses will be recorded as a part of the Medical History and any medications prescribed to treat newly diagnosed diseases will be recorded (See Section 5.3.6 Prior and Concomitant Therapy).

Pregnant or lactating women can be enrolled in this trial. Both the mother and the child should be evaluated at each study evaluation (see Section 5.4.1.5)

Subjects meeting all eligibility criteria will then be assigned a specific study number in sequence and randomized to either DB289 or pentamidine treatment (see Section 5.3.3).

5.4.1.2 Treatment Period

5.4.1.2.1 Study Drug Administration

DB289 will be given to the patient under direct observation of an authorized staff member at approximately 09:00 and 17:00 hours each day for 10 days. The treatment will be started in the evening of Day 1.

Pentamidine will be injected via the intramuscular route every morning at approximately 09:00 hours daily from Day 1 to Day 7.

One hour before treatment start (Day 1), an ECG will be performed. One hour after the first dose of study drug administration, the vital signs will be assessed and recorded. On subsequent treatment days (Day 2-7 for pentamidine and Day 2-11 for DB289), the vital signs will be assessed and recorded 1 hour after the morning dose of study drug.

Treatment compliance will be guaranteed by the direct administration of the drugs exclusively by the assigned study center staff.

5.4.1.2.2 Logistics of Treatment

Subjects will be admitted to the facility to undergo pretreatment procedures, and subsequently receive the first dose of study drug on Day 1. Subjects will remain hospitalized and will be discharged one day after the last dose, given all laboratory values are normal or Grade 1 (Day 7 for pentamidine; Day 11 for DB289) or they will be treated as outpatients for the same duration (see Section 5.4.1.1.2). An indemnity will be paid to the patient to cover cost of living expenses for the accompanying family during the treatment and observation period. The indemnity will be adapted according to local conditions and will be stated in the patient information of the respective centers.

5.4.1.2.3 Procedures and Observations

1. The subjects will receive either DB289 or pentamidine treatment according to their randomization.
2. Any concurrent medication taken during the treatment period will be recorded.
3. Any adverse signs and symptoms reported by the patient or noted during contact with the patient arising during the treatment period will be recorded. Refer to Appendix 4 for information regarding adverse event reporting requirements.
4. Vital signs will be recorded daily in the morning after each treatment dose (Days 1-11 for DB289 treatment group and Days 1-7 for pentamidine treatment group.)
5. Pregnant or lactating female subjects will undergo pharmacokinetic blood or milk sampling as outlined in Section 5.5.4.1.

5.4.1.3 End of Treatment

On study Day 7 for the pentamidine treatment group and Day 11 for the DB289 treatment group, the following battery of tests will be performed in the order indicated. If a test is found to be positive for trypanosomes, subsequent tests need not be performed.

- Microscopic examination of blood (thin and/or thick smear)
- Hematocrit centrifugation of blood (WOO)
- Microscopic examination of lymph node aspirate
- Microscopic examination of blood after m-AECT miniature anion exchange concentration

When a lymph node aspirate is not feasible (for example absence of adenopathy or node impossible to puncture) this will be noted.

The subjects enrolled at a site properly equipped will undergo the following exams:

- Hematology: hemoglobin
- Chemistry profile: serum glucose, creatinine, AST, ALT, total bilirubin
- ECG

In case of laboratory values = Grade 2, repeat two days thereafter. In case of treatment failure, 5 ml of whole blood will be collected for PCR testing. The method is experimental and not yet validated.

For female subjects, a urine pregnancy test will be performed.

Pregnant or lactating female subjects will undergo pharmacokinetic blood or milk sampling as outlined in Section 5.5.4.1.

Signs or symptoms of HAT will be queried and graded as outlined in Appendix 12: lymphadenopathy, temperature, headache, pruritus, daytime sleep, nighttime sleep, tremor, speech impairment, abnormal movements, walking disability, general motor weakness, unusual behavior, inactivity, aggressive behavior, and disturbance of appetite.

Concurrent medication taken during the study post treatment period (last day of study drug through discharge from the treatment center) will be noted.

Any adverse signs and symptoms reported by the patient or noted during contact with the patient arising between the initial dose of study drug and the End of Treatment evaluation will be recorded. For the purpose of this trial, disease progression and relapse will be considered as treatment failure, not as an adverse event. Refer to Appendix 4 for information regarding adverse event reporting requirements.

Before final discharge from the treatment center, a physical examination, including vital signs and Glasgow Coma Scale, will be performed. Any deterioration in physical examination compared to the baseline examination should be reported as an adverse event.

All subjects and parents or guardians of adolescents will be educated to watch for potential adverse events that may develop after the patient is discharged from the treatment center, including signs and symptoms of hepatitis, hypoglycemia or hyperglycemia. Should new adverse events present, the subjects should immediately return to the treatment center for evaluation.

5.4.1.4 Subject Long-Term Follow-up

The treating organization is responsible for the correct follow up of the subjects according to the National Rules. In addition to the regular forms of the National Sleeping Sickness Program, the supplementary CRF forms provided for follow up by STI / Immtech International, Inc. must be used for each patient.

Subjects ages 12 -15 will undergo the following exams at the 3 month post treatment visit:

- Hematology: hemoglobin
- Chemistry profile: serum glucose, creatinine, AST, ALT, total bilirubin
- ECG

Parasitological cure/treatment failure will be assessed by examination for the presence of parasite in blood and lymph node aspirate at 3, 6, 12 and 18 months post treatment. The following battery of tests will be performed in the order indicated:

- Microscopic examination of blood (thin and/or thick smear)
- Hematocrit centrifugation of blood (WOO)
- Microscopic examination of lymph node aspirate
- Microscopic examination of blood after m-AECT miniature anion exchange concentration

When a lymph node aspirate is not feasible (for example absence of adenopathy or node impossible to puncture) this will be noted.

A lumbar puncture will be done at month 6, 12, and 18 months post treatment. The WBC in the CSF will be counted. Microscopic examination of CSF for trypanosomes will be performed (whenever possible, modified concentration technique should be done). A latex IgM test of the CSF will be done.

At month 24 an oral interview and physical examination will be performed. In case a lumbar puncture can be performed at this evaluation, the data will be recorded.

Signs and symptoms typical of HAT will be queried and graded as outlined in Appendix 12: lymphadenopathy, temperature, headache, pruritus, daytime sleep, nighttime sleep, tremor, speech impairment, abnormal movements, walking disability, general motor weakness, unusual behavior, inactivity, aggressive behavior, and disturbance of appetite.

For the purposes of determining appropriate clinical evaluation and follow up, subjects will be classified according to the following descriptions as favorable evolution, uncertain evolution, probably relapse, relapse, or death (Table 5.4.1.4.a), and followed at the indicated intervals or treated as indicated under “Action”. Subjects who are classified

as “Uncertain Evolution” will be followed at more frequent intervals than mandated by the protocol.

Table 5.4.1.4.a Classification of first stage patients during follow up & actions taken

**Category**	**Patient Characteristics**
Favorable Evolution	• Only for patients who undergo lumbar puncture (no hemorrhagic LP)<br>• Patients with = 5 WBC/µl CSF and no parasitological evidence of relapse<br>• **Action**: Continue follow up as scheduled
Uncertain Evolution	• Patients without parasitological evidence of relapse AND with 6-20 WBC/µl CSF<br>• Patients without parasitological evidence of relapse in blood and lymph who refuse lumbar puncture OR whose CSF sample is hemorrhagic AND<br>• who do not present clinical signs of HAT and/or a marked clinical deterioration compared to the previous evaluation AND who in the opinion of the investigator do not require immediate rescue treatment<br>• Patients without parasitological evidence of relapse in blood and lymph AND with = 5 WBC/µl CSF AND an at least fourfold increase in the Latex IgM CSF titer compared to the last evaluation<br>• **Action**: Additional follow up after 1-3 months with clinical evaluation and evaluation of blood, lymph and CSF.
Probable Relapse	• Patients without parasitological evidence of relapse AND with > 20 WBC/µl CSF<br>• Patients without parasitological evidence of relapse in blood and lymph who refuse lumbar puncture OR whose CSF sample is hemorrhagic AND who in the opinion of the investigator require immediate rescue treatment based on e.g. prominent clinical signs of HAT and/or a marked deterioration of their clinical condition (relative to the previous visit) unlikely to be due to another disease than HAT<br>• **Action**: Rescue treatment
Relapse	Trypanosomes have been detected in any body fluid<br>Action: Rescue treatment
Death	Death of patient during treatment or follow up; death will be categorized based on likely or definite cause of death as:<br>HAT<br>Adverse event of treatment of HAT<br>HAT and treatment unrelated causes<br>Unknown causes

In case of suspicion of relapse, 5 ml of whole blood will be collected for PCR testing. The method is experimental and not yet validated.

Any adverse signs and symptoms which are spontaneously reported between the End of Treatment evaluation and 30 days post treatment will be recorded. The Investigator will report all serious adverse events, regardless of the time of the event relative to the completion of treatment. Refer to Appendix 4 for information regarding adverse event reporting requirements.

The patient will be asked about his/her current health status (well or unwell). If the patient is not available at any post treatment evaluation, data about the status of the patient (alive and well or unwell; death and cause of death) will be gathered from family, friends or local authorities.

5.4.1.5 Monitoring of Children of Pregnant and Lactating Women

Information on pregnancy, delivery and early childhood development will be collected at each scheduled evaluation (Baseline, End of Treatment, and each Follow up) to include:

- Pregnancy
- Delivery
- Presence of birth defect(s)
- Early development

All children of women who are pregnant or lactating at the time of treatment will be evaluated at each follow-up visit (3 months to 24 months post treatment).

5.4.1.6 Monitoring for Safety

Treatment emergent adverse signs or symptoms will be recorded in the adverse event section of the Case Report Form, along with date(s) of occurrence, duration, degree of severity, and probable relationship to study drug.

The observation time for adverse events starts when the treatment is initiated and continues until discharge from the facility at the End of Treatment. Any adverse signs and symptoms which are spontaneously reported between the End of Treatment evaluation and 30 days post treatment will be recorded. The Investigator will report all serious adverse events, regardless of the time of the event relative to the completion of treatment. For the purpose of this trial, disease progression and relapse will be considered as treatment failure, not as an Adverse Event.

Toxicity will be graded on a scale of 0 (no toxicity) to 4 using criteria in Appendix 3. Actions to be taken by the Investigator in response to any toxicity or adverse experience that is judged to be at least possibly related to the study medications are based on the

grade level assigned, according to the directions outlined in the next table, Table 5.4.1.6.a. For adverse effects that are not listed, the grade of toxicity will be assigned according to the definitions outlined as follows:

Table 5.4.1.6.a Toxicity Grading and Actions Taken in Response to Toxicity

**Toxicity**	**Action Taken**
Grade 1:<br>Mild toxicity, usually transient, requiring no special treatment and generally, not interfering with usual daily activities	Observe patient closely and monitor laboratory parameters as needed. Patient may continue with study medication.
Grade 2:<br>Moderate toxicity ameliorated by simple maneuvers	Observe patient closely and monitor laboratory parameters as needed. Patient may need to discontinue study medication.<br>If grade 2 toxicity is tolerated by patient, action same as grade 1.
Grade 3:<br>Severe toxicity which requires therapeutic intervention and interrupts usual activities; hospitalization may be prolonged.	NOTIFY STUDY COORDINATOR IMMEDIATELY.<br>Withhold test material. Monitor patient until event or toxicity decreases to Grade 2 or less.<br>Discuss with study director whether to reinstitute treatment.
Grade 4:<br>Extremely severe or life-threatening	NOTIFY STUDY COORDINATOR IMMEDIATELY.<br>Discontinue test material treatment; monitor patient closely until event decreases to Grade 2 or less.

5.4.1.7 Collection and Reporting of Adverse Events

Instructions for definitions, collecting and reporting of adverse events are included in Appendix 4.

5.5 *Efficacy and Safety Variables*

5.5.1 Appropriateness of Measurements

Refer to Section 5.1. for a discussion of the measurements to be used in the trial.

5.5.2 Efficacy Variables

5.5.2.1 Primary Efficacy Variables

The primary efficacy variable will be the combined rate of clinical and parasitological cure (Table 5.5.2.a) at the Test of Cure evaluation (12 month evaluation) in the Per Protocol dataset (Section 6.1.1.). The combined rate of clinical and parasitological cure is defined as the proportion of treated subjects who have no clinical signs and symptoms of trypanosomiasis and no evidence for trypanosomes in any body fluid examined at all post treatment evaluations and not treated with other trypanosomiasis agent for any reason (early or late failure). In subjects who have a 12 month lumbar puncture performed, cerebrospinal fluid (CSF) should contain =5 WBC/µl.

5.5.2.2 Secondary Efficacy Variables

Parasitological cure, clinical cure, probable relapse, relapse and death rates at the End of Treatment and at the 3, 6, and 18 month evaluations will also be determined. Parasitological cure, probable relapse, relapse, and death rates will also be assessed at the 12 month Test of Cure evaluation and at the 24 month evaluation; the clinical cure will be considered equivalent to the parasitological cure at the 24 month evaluation.

Clinical Response Definitions, based on WHO Draft Informal Consultation are outlined in Table 5.5.2.a (below). For purposes of statistical analyses, each patient will be defined within one of the following categories at each post treatment assessment, based on the appropriate characteristics.

Table 5.5.2.a Clinical Response Definitions

Category	WHO Term	Patient Characteristics
Parasitological Cure	Cure	Lumbar puncture performed: No evidence for parasitological relapse and = 5 WBC/mm^3 in CSF
Clinical Cure	Probable Cure	No evidence for parasitological relapse in absence of lumbar puncture (no clinical signs; symptoms / signs attributable to other disease; investigator decides no retreatement necessary) **or**<br>No parasitological evidence of relapse with 6-20 WBC/mm^3 in CSF<br>**Action:** No retreatment
Probable Relapse	Probable Relapse	No evidence of parasitological relapse and > 20 WBC/mm^3 in CSF **or**<br>No evidence of parasitological relapse in a patient who refuses lumbar puncture **and** who presents with clinical signs of HAT and/or marked deterioration of clinical condition relative to previous evaluations that is unlikely due to another disease **and** for whose clinical status all other reasons have been excluded in the opinion of the investigator, **and** who in the opinion of the investigator require rescue treatment<br>**Action:** Retreatment
Relapse	Relapse	Trypanosomes have been detected in any body fluid<br>**Action:** Retreatment
Death	Death	Death of patient during treatment or follow up; death will be categorized based on likely or definite cause of death as:<br>HAT<br>Adverse event related to treatment of HAT<br>Causes unrelated to HAT and treatment<br>Unknown causes

5.5.3 Safety Variables

The safety variables which will be evaluated through the End of Treatment evaluation in this Phase 3 study include: adverse events, laboratory results, vital sign measurements, physical examinations, and the use of any concomitant medications. Adverse events which are spontaneously reported between the End of Treatment evaluation and 30 days post treatment will also be collected. The Investigator will report all serious adverse events, regardless of the time of the event relative to the completion of treatment.

5.5.4 Drug Concentration Measurements

5.5.4.1 Pharmacokinetic Procedures

Pharmacokinetic sampling will be conducted only in the sub-populations of pregnant or lactating female subjects who are randomized to treatment with DB289. Blood samples for the determination of plasma concentrations of DB289 and DB75 will be obtained from pregnant and lactating women at study sites where there are adequate facilities to freeze and store the samples after collection. Milk samples will also be obtained from lactating and breast feeding female subjects enrolled at study sites where there are adequate facilities to freeze and store samples. No samples will be collected from subjects who are randomized to treatment with pentamidine.

In recognition of the special status of these patient populations, minimal pharmacokinetic sampling is scheduled. Three samples will be obtained from each eligible patient near the end of the treatment regimen; the samples should be collected between Day 6 and Day 11 of treatment. One sample is to be obtained predose in the morning and one sample 4 to 6 hours post dose (any day of Day 6 through Day 11). One sample will also be obtained 24 hours after the last dose of DB289 on Day 11.

Breast milk (3 samples from each patient) will be collected at similar time points from lactating female subjects.

5.5.4.2 Pharmacokinetic Analysis

Given the sparse sampling procedures involved, the pharmacokinetic analysis will focus on comparisons to previous pharmacokinetic results collected from adult patient populations.

The preferred sampling times have been selected to provide information about the approximate peak concentrations (4 to 6-hour sample) in the pregnant women patient population, and estimates of the extent of drug accumulation (pre-dose and 24 hours after the last dose samples). Comparison to existing pharmacokinetic data will be made to quantify the relative extent of exposure in this special patient population compared to the more general population for which more detailed information exists. In addition, evaluation of the concentration difference between the 4 to 6-hour and 24-hour samples will be used to estimate the elimination rates of DB289 and DB75 in this special population.

The milk samples from the lactating women will be assayed for DB289 and DB75 concentrations. The measured concentrations will be used to estimate total daily exposure of an infant to DB289 and DB75 via its mother's milk. Since no samples are anticipated to be obtained from the breast-feeding infants, the actual systemic exposure will not be able to be determined (i.e., the impact of oral bioavailability, metabolism and excretion patterns in the infant will not be able to be assessed).

5.5.4.2 Handling of samples

Instructions for collecting, storing and shipping plasma samples and breast milk samples are provided in Appendix 8.

6.0 Statistical Methods

6.1 Statistical and Analytical Plan

All tests will be two-tailed at alpha equal to 0.05 unless stated otherwise. P-values will be rounded to three decimal places before assessing for statistical significance.

The percent of subjects who prematurely discontinue treatment and the percent of subjects who are lost to follow-up will be summarized by treatment group. Summaries by reason and across time will be provided as appropriate.

6.1.1 Data Sets Analyzed

Four datasets will be defined for analysis:

- Safety dataset: All subjects who receive randomized study drug and have at least one safety evaluation after dosing.

- Primary Per Protocol dataset: This Per Protocol dataset for the primary efficacy analysis is defined as all subjects who have parasitologically-confirmed infection prior to treatment, who receive a minimum of 7 days of DB289 or 5 injections of pentamidine and who have a Test of Cure assessment at 12 months post treatment or have reached an efficacy endpoint of death, non-response or relapse. Subjects who refuse to undergo lumbar puncture at 12 months post treatment will have a primary efficacy endpoint assessment based on clinical signs and symptoms and parasitological outcome of any body fluid examined, and will be included in the primary analysis. Subjects who receive the defined minimum amount of study drug and subsequently discontinue therapy for any reason will have a primary efficacy endpoint assessment at 12 months and will be included in the analysis. Any subject who discontinues therapy for an adverse event prior to receiving the defined minimum amount of study drug will be considered as a treatment failure in the analysis. No other anti-trypanosomal agent was administered during the period within 8 weeks prior to start of study drug to Test of Cure, unless the subject was considered a study treatment failure.

- Supportive Per Protocol dataset: This Per Protocol dataset for the supportive efficacy analyses is defined as all subjects who receive a

minimum amount of randomized study drug (7 days for DB289 or 5 injections for pentamidine), and have a Test of Cure assessment or have reached an efficacy endpoint (e.g., death, non-response, relapse, etc.). All subjects must have parasitologically confirmed infection with *T. b. gambiense* prior to treatment. No other anti-trypanosomal agent was administered during the period within 8 weeks prior to start of study drug to Test of Cure, unless the subject was considered a study treatment failure. Any subject who discontinues therapy for an adverse event prior to receiving the defined minimum amount of study drug will be considered as a treatment failure in the analysis.

- Modified ITT dataset: All subjects who receive the minimum amount of randomized study drug and for whom an End of Treatment assessment and at least one follow-up efficacy assessment are available. All subjects must have parasitologically confirmed infection with *T. b. gambiense* prior to treatment. Any subject who discontinues therapy for an adverse event prior to receiving the defined minimum amount of study drug will be considered as a treatment failure in the analysis.

- Intent-to-treat (ITT) dataset: All subjects who receive at least one dose of study drug. Subjects who are lost to followup or discontinued from the study for any reason (subjects with missing data) are considered as failures in the ITT dataset. All subjects must have parasitologically-confirmed infection with *T. b. gambiense* prior to treatment.

6.1.2 Demographics and Other Baseline Characteristics

Comparability of baseline characteristics between treatment groups will be assessed for quantitative variables with the one-way analysis of variance (ANOVA) and for qualitative variables with Fisher's exact test (or its generalization to tables larger than 2x2).

6.1.3 Efficacy Analyses

6.1.3.1 Primary Efficacy Analysis

Demonstrating the non-inferiority of DB289 to pentamidine in the combined rate of clinical and parasitological cure is the primary objective of this study. The non-inferiority comparison will be conducted with alpha equal to 0.048 and non-inferiority margin (i.e., delta) of 0.15. The comparison will be made with a one-sided 97.6% confidence interval for the treatment difference in parasitological cure rate. The normal approximation to the binomial distribution with continuity correction will be used to construct the confidence interval. The primary dataset for efficacy analysis will be the Per Protocol dataset as defined in Section 6.1.1.

The delta of 0.15 is an appropriate criterion for establishing non-inferiority of DB289 to pentamidine in first stage HAT. This disease is considered universally fatal[59] if untreated. Therefore, the delta would readily distinguish the efficacy of DB289 from placebo.

A delta of 0.15 between DB289 and the control treatment pentamidine can also be justified by the expected advantages of the new orally administered drug. It will facilitate treatment of much larger numbers of patients, as it can be administered in selected public health facilities, in addition to the limited number of specialized sleeping sickness treatment centers where parenteral treatment is currently administered In addition to the individual benefit to greater numbers of patients, decreasing the number of infected individuals will likely decrease the transmission rate in the endemic foci.

6.1.3.2 Supportive Analyses

Secondary analyses will be conducted to assess the parasitological cure rate at the Test of Cure evaluation. Only subjects who attended the Test of Cure evaluation, and who underwent lumbar puncture, as described in Table 5.5.2.a will be included in this analysis..

Similarly constructed one-sided 97.5% confidence intervals on the treatment difference in parasitological cure rate and clinical cure rate will be provided for the ITT and modified ITT datasets. These additional confidence intervals do not provide definitive evidence of non-inferiority but do provide information on consistency of treatment differences across methods of handling subjects for whom the primary efficacy endpoint is missing. It is anticipated that most of the missing primary endpoints will be due to subjects who cannot be assessed at the Test of Cure evaluation or who decline to undergo lumbar puncture at the Test of Cure evaluation.

Estimates of the clinical cure rate and parasitological cure rate will be provided for subsets of subjects defined by sex, age, body mass index, and country. No formal statistical testing will be conducted for these subset analyses.

The following supportive WHO-defined efficacy endpoints will be summarized at Test of Cure with point estimates and one-sided 97.5% confidence intervals for the difference between treatments.

- Treatment failure rate: death, non-response at end of treatment, relapse, probable relapse
- Relapse rate: relapse and probable relapse
- Parasitologically confirmed relapse rate: relapse
- Probable relapse rate
- Treatment fatality rate: all deaths through Test of Cure evaluation
- Clinical cure rate: cure, probable cure
- Parasitological cure rate (cure in WHO definitions)

All of the preceding endpoints will be summarized for the Per Protocol, ITT, and modified ITT datasets. For modified ITT datasets, missing data will be estimated for both treatment groups according to the LOCF principle in the WHO Draft Informal Consultation of 09Sep04. Descriptive summaries of these variables will be provided at evaluations before Test of Cure as appropriate. In addition, the treatment response rate will be summarized at the End of Treatment evaluation.

Last observation carried backwards may be used to account for missing data at an earlier evaluation. For example, a subject who does not attend the 12 month evaluation, but attends the 18 month evaluation will be considered evaluable for the 12 month evaluation and the parasitological outcome at the 18 month evaluation will be used for the missing data.

6.1.4 Safety Analyses

The safety dataset will consist of all subjects who receive randomized study drug and have at least one safety evaluation after dosing.

6.1.4.1 Adverse Events

Adverse events will be coded with MedDRA, and the percentage of subjects reporting treatment emergent adverse events will be summarized for each treatment group at the System Organ Class (SOC) and High Level Group Term (HLGT) levels. Treatment emergent adverse events are defined as adverse events that begin or worsen in severity after the first dose of study drug. Treatment group differences at the HLGT level will be assessed with Fisher's exact test.

Treatment emergent adverse events will be summarized descriptively by investigator-specified relationship to study drug, severity, and time. Severity will be categorized by WHO toxicity grade. Incidence is defined as the percentage of subjects who first report the adverse event during the treatment period (e.g., Days 1-11).

The incidence of serious adverse events will be summarized for each treatment group at the SOC and HLGT levels for Days 1-11, Day 12 through Test of Cure, and for Day 1 through Test of Cure. The incidence of adverse events resulting in study drug discontinuation will be summarized for Days 1-11.

6.1.4.2 Clinical Laboratory Results

The mean, standard deviation and other descriptive statistics will be calculated for clinical laboratory results at all scheduled assessments. The treatment group difference of laboratory parameters in mean change from baseline to each scheduled assessment will be tested statistically with the one-way ANOVA. Baseline will be defined as the last clinical laboratory result obtained before study drug is given. If 2 or more laboratory results are assigned to the same scheduled assessment, the earliest result will be used in the statistical analysis.

The percentage of subjects whose baseline laboratory result is within the normal laboratory reference range at baseline but meets WHO toxicity grade III or higher at later scheduled assessments will be summarized for each treatment group at each scheduled assessment.

6.1.4.3 Vital Signs and ECG

Vital signs and ECG will be summarized in the same manner as clinical laboratory results. The QT interval of the ECG will be adjusted for heart rate with the Bazett, Fridericia, and Framingham formulae.

6.1.5 Interim Analyses

An interim analysis will be conducted when one-half of the enrolled subjects have reached the 12 month post treatment endpoint. The sponsor will remain blinded to these data. These data will be provided to the DSMB for evaluation. Based on these data, the DSMB will make appropriate recommendations to the sponsor regarding continuation of the study. The study may be stopped if:

- any new untoward safety issues are identified in the DB289 treatment group such that DB289 would be significantly less safe than pentamidine.
- the re-estimated sample size exceeds 500 subjects to achieve 90% power for the primary efficacy endpoint
- efficacy analysis indicates that DB289 is significantly more effective than pentamidine ($p<0.002$).

A preliminary report of Test of Cure results will be provided to regulatory authorities once all subjects have completed the 12 month post treatment evaluation. This report will provide primary evidence for accelerated approval of DB289 in the treatment of first stage HAT caused by *T. b. gambiense*. The final report of study results will be provided to regulatory authorities once all subjects have completed the 24 month post treatment evaluation. The final report will provide primary evidence for final approval of DB289. No p-value adjustment will be made for the preliminary report.

6.1.6 Determination of Sample Size

A total of approximately 250 subjects, 125 subjects per treatment group, will be treated with study drug. Of these, an estimated 200 subjects, 100 per treatment group, will be included in the Primary Per Protocol dataset. This sample size provides more than 90% power to demonstrate non-inferiority of DB289 to pentamidine for the primary endpoint, when the study drugs have equivalent probable cure rates of 95% in the Primary Per Protocol population.

The literature estimates of efficacy for pentamidine are highly variable. Older studies have rates ranging from 16% to 100%[27,28,29], but cannot be used to predict current efficacy. Recent literature gives ranges from 94%[4] to 97%[62], and the recent Phase 2b study has shown efficacy of 97.5% (one relapse of 41 subjects) at the 12 month follow up, with 75% of the subjects having completed this evaluation as of 15 Dec 2004. The use of more sensitive diagnostic tests, such as the m-AECT[55], and particularly using accurate cell counts[2,3] in of the CSF to exclude potential second stage disease, have improved the ability to select appropriate first stage patients for clinical trials, and exclude second stage patients, who require different therapy. Therefore, 95% has been chosen as the expected efficacy of pentamidine in this study.

7.0 Protocol Deviations

When a variation from the protocol is deemed necessary for an individual subject, the investigator or other physician in attendance must contact one of the Medical Monitors listed in Appendix 4 (Section 4.4).

Such contact must be made as soon as possible to permit a decision as to whether or not the subject is to continue in the study. The deviation from the protocol will be authorized only for that subject.

All deviations related to study inclusion and exclusion criteria and significant deviations to subject management and protocol procedures must be documented on the appropriate case report form.

Refer to Appendix 6 for further information regarding changes in protocol.

8.0 Ethics and Regulatory Requirements

8.1 Independent Ethics Committee (IEC) or Institutional Review Board (IRB)

Good Clinical Practice requires that the clinical protocol, any protocol amendments, the Investigator's Brochure, the Informed Consent, and all other forms of subject information related to the study and any other necessary documents be reviewed by an Independent Ethics Committee (IEC) or Institutional Review Board (IRB). Specific information regarding investigator responsibilities regard IEC/IRB review and approval are provided in Appendix 5.

8.2 Ethical Conduct of the Study

The study will be conducted in accordance with the protocol, all ICH and GCP regulations governing clinical study conduct; ethical principles that have their origin in the Declaration of Helsinki (Appendix 5), and all applicable local laws and regulations. The investigator must assure that the study is conducted in accordance with the

provisions as stated in the US FDA regulations and complies with prevailing local laws and customs. Responsibilities of the Investigator are specified in Appendix 6.

8.3 Subject Information and Consent

The investigator or his/her representative will explain the nature of the study to the subject, and answer all questions regarding this study, prior to obtaining informed consent. Specific instructions for obtaining informed consent are included in Appendix 5.

8.4 Subject Confidentiality

The investigators must ensure that the subject's anonymity will be maintained. Subjects will be identified on the Case Report Forms by the Subject Number and Subject's Initials in addition to center and study identification information. The investigators will keep a separate confidential enrollment log that matches identifying codes with the subjects' names and residencies.

8.5 Data and Safety Monitoring Board (DSMB)

A formal Data and Safety Monitoring Board (DSMB) will be established to oversee the trial. The DSMB will establish a charter and formal stopping rules for the trial.

8.6 Investigator(s) and Study Site(s)

Investigative sites will be selected by STI and Immtech International, Inc. Five to seven sites in DR Congo, Angola and South Sudan will be selected to enroll subjects for this study. Investigators will be selected on their ability to enroll subjects as well as the adequacy of their sites to manage study-related activities and requirements
(see Appendix 5).

9.0 Data Quality Assurance

Refer to Appendix 6 for information regarding Data Quality Assurance.

10.0 Other Administrative and Regulatory Procedures

10.1 Source Documents, Case Report Form Completion, Monitoring and Inspections, and Maintenance of Records

Refer to Appendix 6 for instructions regarding Source Documents, Case Report Forms, Monitoring and Inspections, and Maintenance of Records

10.2 Completion of the Study

The investigator will conduct this study in compliance with the protocol, and will complete the study in satisfactory compliance with the protocol within 8 weeks after the last evaluation of the last subject or within 8 weeks of the designated completion date. Continuation of the study beyond this time must be mutually agreed upon in writing by both the investigator and Immtech International, Inc. The investigator will provide a summary of the study's outcome to the IEC/IRB following conclusion of the study, and will forward a copy of this summary to Immtech International, Inc. or their designee.

10.2.1 Final Report

Refer to Appendix 6 regarding the Final Study Report.

10.2.2 Use of Information

Requirements for use of confidential information are provided in Appendix 6.

10.3 Publication

Information regarding publication of the results of this study is outlined in Appendix 9.

10.4 Sponsor Information

The sponsor, Immtech International, Inc., will coordinate the activities for initiating this multi-center clinical study. The administration of the study will be managed by Immtech International, Inc. and Swiss Tropical Institute (CRO). The protocol will be prepared by Immtech International, Inc in consultation with STI. A statistician identified by Immtech International, Inc. will be responsible for the statistical analyses of the data.

10.5 Contract Research Organization

Immtech International, Inc. will assign the obligation of qualification of investigators and investigative sites, pre-study visits, initiation visits, site monitoring, post-study visits, as well as clinical trial supply accountability to STI (the CRO). In addition, STI will be responsible for developing the Case Report Forms and sample informed consent form. The Sponsor (Immtech International, Inc.) and CRO (STI) will maintain constant contact for adequate management of the study progress.

10.6 Clinical Supply Management

Refer to Appendix 6, Disposition of Clinical Supplies.

10.7 Laboratories

10.7.1 Clinical Laboratory Tests and Normal Laboratory Values

Safety laboratory testing of blood samples (hematology, chemistry) will be performed by local laboratories. Refer to Appendix 6 for further information regarding clinical laboratory tests and normal laboratory values.

10.7.1 Identification of Laboratories

Tandem Labs will be responsible for analysis of the pharmacokinetic samples of blood and breast milk collected during the trial. Information about the laboratory, including contact personnel and shipping address, is included in Appendix 8.

10.7.2 Clinical Supply Management

Immtech International, Inc., either directly or by delegation to STI, will prepare or send all clinical supplies to investigative sites for the study. Immtech International, Inc. will delegate to STI the authority to release the clinical supplies to each site when the appropriate essential documents have been received from the respective sites (see Appendix 10).

11.0 Country Specific Investigator's Signature Page

1. I have received and reviewed the DB289 Investigator's Brochure.
2. I have read this protocol and agree to conduct the study as outlined and in accordance with all applicable local, state, and federal regulations.
3. I agree to maintain the confidentiality of all information received or developed in connection with this protocol.

____ ____________________

Signature of Local Principal Investigator Date

____

Printed Name of Local Principal Investigator

____ ____________________

Signature of Country Coordinator Date

____

Printed Name of Country Coordinator

Appendix 1 Guidance to Investigator for Exclusion Criteria Number 2

The objective of the exclusion criteria is to exclude patient with significant organ dysfunction. Because of the local conditions and the absence of laboratory equipment, this exclusion will be assessed mainly on patient medical history and careful collection of signs and symptoms.

Coagulation: current or history of bleeding (e.g. epistaxis, hematemesis)
Nutrition: adequate food intake not possible
Diarrhea: = 7 stools per day, or incontinence or severe abdominal cramps
Vomiting: = 2 episodes per day
Stomatitis: painful erythema, edema or ulcers, cannot eat solids
Liver: clinical jaundice
Proteinuria: = ++
Hematuria: gross
Diabetes and/or Glycosuria: = ++
Bilirubinuria: = ++
Dyspnea when walking at normal speed
Cyanosis
Respiratory rate = 24 / min at rest
Angina even without sign of infarction
Recurrent arrhythmia upon auscultation or clinical palpitations
Symptomatic pericarditis (rub, pain, effusion)
Hypertension with systolic blood pressure = 180
Congestive heart failure
Tachycardia (heart rate = 120)
Skin: generalized symptomatic eruption
Chronic fever: = 39ºC
Neurological: unable to walk, severe somnolence, agitation, confusion, disorientation or hallucination
History or current seizure disorder
Infection: Ongoing acute infection requiring treatment, not curable with short term treatment on site before application of trypanocidal drugs. Known HIV infection

Appendix 2 Glasgow Coma Scale (adapted)

Adults	Reaction	Score
**Best Verbal Response**	**Oriented**	**5**
	**Confused**	**4**
	**Inappropriate Words**	**3**
	**Incomprehensible Sounds**	**2**
	**None**	**1**
**Best Motor Response**	**Obeys Commands**	**6**
	**Directed Defensive Response**	**5**
	**Non-directed Defensive Response**	**4**
	**Flexion to Pain**	**3**
	**Extension to Pain**	**2**
	**None**	**1**
	**Total**	**2-11**
	**(considered unrousable coma**	**= 7)**

Appendix 3 Toxicity Grading Scale

WHO (World Health Organization) Toxicity Criteria by Grade

Category	Toxicity	Grade 0	Grade 1	Grade 2	Grade 3	Grade 4
Haematology	WBC ($x10^3$/l)	> 4	3.0 - 3.9	2.0 - 2.9	1.0 - 1.9	< 1.0
Haematology	Platelets ($x10^3$/l)	WNL	75.0 - normal	50.0 - 74.9	25.0 - 49.9	< 25.0
Haematology	Haemoglobin (g/dl)	WNL	10.0 - normal	8.0 - 9.9	6.5 - 7.9	< 6.5
Haematology	Granulocytes/ Bands ($x10^3$/l)	> 2	1.5 - 1.9	1.0 - 1.4	0.5 - 0.9	< 0.5
Haematology	Lymphocytes ($x10^3$/l)	> 2	1.5 - 1.9	1.0 - 1.4	0.5 - 0.9	< 0.5
Haematology	Haemorrhage	None	mild, no	gross, 1 - 2 units transfusion per episode	gross, 3 - 4 units transfusion per episode	massive, > 4 units transfusion per episode
Coagulation	Fibrinogen	WNL	0.99 - 0.75 x N	0.74 - 0.50 x N	0.49 - 0.25 x N	< 0.25 x N
Coagulation	Prothrombin time(Quick)	WNL	1.01 - 1.25 x N	1.26 - 1.50 x N	1.51 - 2.00 x N	> 2.00 x N
Coagulation	Partial thrombo-plastin time	WNL	1.01 - 1.66 x N	1.67 - 2.33 x N	2.34 - 3.00 x N	> 3.00 x N
Metabolic	Hyperglycaemia (mg/dl)	< 116	116 - 160	161 - 250	251 - 500	> 500 or ketoacidosis
Metabolic	Hypoglycaemia (mg/dl)	> 64	55 - 64	40 - 54	30 - 39	< 30
Metabolic	Amylase	WNL	< 1.5 x N	1.5 - 2.0 x N	2.1 - 5.0 N	> 5.0 x N
Metabolic	Hypercalcaemia (mg/dl)	< 10.6	10.6 - 11.5	11.6 - 12.5	12.6 - 13.4	13.5
Metabolic	Hypocalcaemia (mg/dl)	> 8.4	8.4 - 7.8	7.7 - 7.0	6.9 - 6.1	6
Metabolic	Hypomagnesaemia (mg/dl)	> 1.4	1.4 - 1.2	1.1 - 0.9	0.8 - 0.6	0.5
Gastrointestinal	Nausea	None	able to eat reasonable intake	intake significantly decreased but can eat	no significant intake	—
Gastrointestinal	Vomiting	None	1 episode in 24 hrs	2 - 5 episodes in 24 hrs	6 - 10 episodes in 24 hrs	> 10 episodes in 24 hrs or requiring parenteral support

Appendix 3 Toxicity Grading Scale (continued)

Category	Toxicity	Grade 0	Grade 1	Grade 2	Grade 3	Grade 4
Gastrointestinal	Diarrhoea	None	increase of 2 - 3 stools / day over pre-Rx	increase of 4 - 6 stools / day, or nocturnal stools, or moderate cramping	increase of 7 - 9 stools / day, or incontinence, or severe cramping	increase of > 10 stools / day or grossly bloody diarrhoea, or need for paren-teral support
Gastrointestinal	Stomatitis	None	painless ulcers, erythema, or mild soreness	painful erythema, oedema, or ulcers but can eat solids	painful erythema, oedema, or ulcers and cannot eat solids	requires paren-teral or enteral support for alimentation
Liver	Bilirubin (N = 17 µmol/L)	WNL	-----	< 1.5 x N	1.5 - 3.0 x N	> 3.0 x N
Liver	Transaminase (SGOT, SGPT)	WNL	2.5 x N	2.6 - 5.0 x N	5.1 - 20.0 x N	> 20.0 x N
Liver	Alk Phos or 5 nucleotidase	WNL	< 2.5 x N	2.6 - 5.0 x N	5.1 - 20.0 x N	> 20.0 x N
Liver	Liver- clinical	No change from baseline	-----	-----	precoma	hepatic coma
Kidney, bladder	Creatinine	WNL	< 1.5 x N	1.5 - 3.0 x N	3.1 - 6.0 x N	> 6.0 x N
Kidney, bladder	Proteinuria	No change	1 (+) or < 0.3 g% or 3 g/L	2 - 3 (+) or 0.3 - 1.0 g% or 3 - 10 g/L	4 (+) or > 1.0 g% or > 10g/L	nephrotic syndrome
Kidney, bladder	Haematuria	Negative	microscopic only	gross, no clots no Rx needed	gross and clots bladder irrigation	requires trans-fusion or cystectomy
Kidney, bladder	Weight gain/ loss	< 5.0 %	5.0 - 9.9 %	10.0 - 19.9 %	20.00%	-----
Pulmonary	Pulmonary	none or no change	asymptomatic, with abnormal-ity in PFTs	dyspnoea on significant exertion	dyspnoea at normal level of activity	dyspnoea at rest
Cardiac	Cardiac arrhythmias	none	asymptomatic, transient, requiring no therapy	recurrent or persistent, no therapy required	requires treatment	requires moni-toring; or hypo-tension, or ventricular tachycardia or fibrillation
Cardiac	Cardiac function	none	asymptomatic, decline of resting ejection fraction by less than 20 % of baseline value	asymptomatic, decline of resting ejection fraction by more than 20 % of baseline value	mild CHF, responsive to therapy	severe of refractory CHF
Cardiac	Cardiac ischaemia	none	non-specific T-wave flattening	asymptomatic, ST and T wave changes suggesting ischaemia	angina without evidence of infraction	acute myocardial infarction

Appendix 3 Toxicity Grading Scale (continued)

Category	Toxicity	Grade 0	Grade 1	Grade 2	Grade 3	Grade 4
Cardiac	Cardiac-pericardial	none	asymptomatic effusion, no intervention required	pericarditis (rub, chest pain, ECG changes)	symptomatic effusion; drainage required	tamponade; drainage urgently required
Cardiac	Hypertension	none or no change	asymptomatic, transient increase by greater than 20 mm Hg (D) or to > 150 / 100 if previously WNL. No treatment required.	recurrent or persistent increase by greater than 20 mm HG (D) or to > 150 / 100 if previously WNL. No treatment required.	requires therapy	hypertensive crisis
Cardiac	Hypotension	none or no change	changes requiring no therapy (incl-uding transient orthostatic hypo-tension)	requires fluid replacement or other therapy but not hospitalisation	requires therapy and hospitalisation; resolves within 48 hours of stopping the agent	requires therapy and hospitalis- ation for > 48 hrs after stopping the agent
Neurologic	Neuro: sensory	none or no change	mild paraesthesias; loss of deep tendon reflexes	mild or moderate objective sensory loss moderate paraesthesias	severe objective sensory loss or paraesthesias that interfere with function	-----
Neurologic	Neuro: motor	none or no change	subjective weak-ness; no objective findings	mild objective weakness without significant impair- ment of function	objektive weak-ness with impairment of function	paralysis
Neurologic	Neuro: cortical	none	mild somnolence or agitation	moderate somnolence or agitation	severe somnolence, (>50 % waking hours), agitation, confusion, disorientation or hallucinations	coma, seizures, toxic psychosis
Neurologic	Neuro: cerebellar	none	slight incoordination, dysdiadochokinesia	intention tremor, dysmetria, slurred speech, nystagmus	locomotor ataxia	cerebellar necrosis
Neurologic	Neuro: mood	no change	mild anxiety or depression	moderate anxiety or depression	severe anxiety or depression	suicidal ideation

Appendix 3 Toxicity Grading Scale (continued)

Category	Toxicity	Grade 0	Grade 1	Grade 2	Grade 3	Grade 4
Neurologic	Neuro: headache	none	mild	moderate or severe but transient	unrelenting and severe	-----
Neurologic	Neuro: constipation	none or no change	mild	moderate	severe	ileus > 96 hrs
Neurologic	Neuro: hearing	none or no change	asymptomatic, hearing loss on audiometry only	tinnitus	hearing loss interfering with function but correctable with hearing aid	deafness not correctable
Neurologic	Neuro: vision	none or no change	-----	-----	symptomatic subtotal loss of vision	blindness
Pain	Pain	none	mild	moderate	severe	reg. narcotics
Skin	Skin	none or no change	scattered macular ot papular eruption or erythema that is asymptomatic	scattered macular or papular eruption or erythema with pruritus or other associated symptoms	generalised symptomatic macular, papular or vesicular eruption	exfoliative dermatitis or ulcerating dermatitis
Alopecia	Alopecia	no loss	mild hair loss	pronounced or total hair loss	-----	-----
Allergy	Allergy	none	transient rash, drug fever < 38°C (100.4°F)	urticaria, drug fever 38°C (100.4°F), mild bronchospasm	serum sickness, bronchospasm requiring parenteral medication	anaphylaxis
Local	Local	none	pain	pain and swelling with inflammation or phlebitis	ulceration	plastic surgery indicated
Fever of unknown origin	Fever of unknown origin	none	37.1 - 38.0°C 98.7 - 100.4°F	38.1 - 40.0°C 100.5 - 104°F	> 40.0°C (> 104.0°F) for less than 24hrs	> 40.0°C (>104°F) for more than 24 hrs or accompanied by hypotension
Infection	Infection	none	mild	moderate	severe	life-threatening

Appendix 4 Administrative Procedures for the Reporting of Adverse Events

4.1 Adverse Events

The Investigator will be required to provide appropriate information concerning any findings that suggest significant hazards, contraindications, side effects, or precautions pertinent to the safety of the drug under investigation.

4.2 Types of Adverse Events

The term “adverse event” could include any of the following events which develop or increase in severity during the course of the study:

a. Any signs or symptoms whether thought to be related or unrelated to the condition under study;
b. Any clinically significant laboratory abnormality;
c. Any abnormality detected during physical examination.

These data will be recorded on the appropriate CRFs, regardless of whether they are thought to be associated with the study or the drug under investigation. Associated with the use of the drug means that there is a reasonable possibility that the event may have been caused by the drug.

Adverse signs or symptoms will be graded by the Investigator as mild, moderate, severe or intolerable according to the following definitions:

Grade	Definition
Mild (1):	Causing no limitation of usual activities.
Moderate (2):	Causing some limitation of usual activities.
Severe (3):	Causing inability to carry out usual activities.
Intolerable (4):	Intolerable or life threatening.

The observation time for adverse events starts when the treatment is initiated and continues until discharge from the facility at the End of Treatment. Any adverse signs and symptoms which are spontaneously reported between the End of Treatment evaluation and 30 days post treatment will be recorded. For the purpose of this trial, disease progression and relapse will be considered as treatment failure, not as an Adverse Event.

4.3 Serious Adverse Events

A "serious" adverse event is defined as any event that suggests a significant hazard, contraindication, side effect, or precaution. A serious adverse event includes any event that:

1. is fatal;
2. is life threatening, meaning, the subject was, in the view of the Investigator, at immediate risk of death from the reaction as it occurred, i.e., it does not include a reaction that, had it occurred in a more serious form, might have caused death;
3. is a persistent or significant disability or incapacity, i.e., the event causes a substantial disruption of a person's ability to conduct normal life functions;
4. requires, or prolongs in-patient hospitalization;
5. is a congenital anomaly or birth defect;
6. is an important medical event, based upon appropriate medical judgment, that may jeopardize the patient or subject or may require medical or surgical intervention to prevent one of the other outcomes defining serious.

An unexpected event is any adverse event that is not identified in nature, severity, or frequency in the Investigational Brochure.

A "severe" adverse event does not necessarily meet the criteria for a "serious" adverse event.

4.4 Reporting Obligations

All "Serious" events, whether or not unexpected or considered to be associated with the use of the drug, and regardless of the timing of their occurrence after the first dose of study drug, must be communicated immediately upon discovery of the event, and within 24 hours of discovery, either by telephone or fax.

Contact: Study Coordinator
Gabriele Pohlig, PhD
Swiss Tropical Institute,
Socinstrasse 57,
CH-4002 Basel,
Fax +41 61 225 26 78,
Tel +41 61 225 26 62
e-mail: Gabriele.Pohlig@unibas.ch

Alternative: Study Director
Christian Burri, MSc, PhD
Swiss Tropical Institute,
Socinstrasse 57,
CH-4002 Basel,
Fax +41 61 225 26 78
Tel +41 61 225 26 61
e-mail: Christian.Burri@unibas.ch

Back up (night, weekends):

Doctor of the STI on Call at +41 79 200 25 07 (cell phone of Dr. J. Blum)
Doctor of the STI on Call at +41 61 284 81 44 (emergency phone STI)

The Study Director will inform the sponsor and file the report with the Ethics Committee, the Scientific Advisory Board at UNC and the Data and Safety Monitoring Board.

The Study Coordinator will then advise the Investigator regarding the nature of any further information or documentation that is required.

Each Investigator will receive notification of these events, and each Investigator must promptly inform his or her Ethics Committee of any serious, unexpected adverse event that is considered possibly related to the study drug.

4.5 Follow-up of Adverse Events

All Serious Adverse Events must be followed with appropriate medical management until resolved or until considered chronic and stable or otherwise explained If the treatment was interrupted due to an adverse event, it may be resumed if considered both safe and ethical. The minimum coherent duration of treatment must be maintained if the patient is retreated.

Appendix 5 Ethical Considerations and Human Subject Protection

5.1 Investigators and Study Sites

The Investigators who are responsible for the conduct of this study, in compliance with this protocol, are identified on the Signatures of Agreement page.

5.2 Ethics Committee Acceptance

It is required that a valid Ethics Committee approves in writing the conduct of this clinical study, together with the Investigator's informed consent document, prior to study initiation.

The trial protocol was developed in accordance with the Declaration of Helsinki, the ICH Guidance on Good Clinical Practice and the World Health Organization Guidelines for Good Clinical Practice.

In performing this study, both the Investigator and Sponsor endorse, as a minimum, the standards for conduct of clinical research activities as set forth in the Declaration of Helsinki, ICH guidelines and local country laws and regulations.

The Study Director will submit the protocol and informed consent for Ethics Committee acceptance. This will be appropriately documented. The Ethics Committee should be asked to give its acceptance in writing. The names and qualifications of the members of the review committee will be recorded and submitted to Immtech International, Inc. together with the written acceptance for the conduct of the study. The members of the Ethics Committee accepting must be independent of the sponsor and the Investigator. The written acceptance should consist of a completed Institutional Review Board / Ethics Committee Acceptance form or written documentation from the Ethics Committee containing the same information.

Until written acceptance by the Ethics Committee has been received by the Sponsor, no subject may undergo any procedures solely for the purpose of determining eligibility for this study.

Protocol amendments must also be reviewed and accepted by the Ethics Committee and written acceptance from the committee or at least the chairperson (or a designated committee member) must be received by Immtech International, Inc. before implementation. This written approval will consist of a completed Institutional Review Board Approval / Ethics Committee Acceptance form or written documentation from the Ethics Committee containing the same information.

5.3 Declaration of Helsinki

World Medical Association Declaration of Helsinki: Recommendations Guiding Physicians in Biomedical Research Involving Human Subjects

Adopted by the 18th World Medical Assembly, Helsinki, Finland, 1964 and as revised by the World Medical Assembly in Tokyo, Japan in 1975, in Venice, Italy in 1983, and in Hong Kong in 1989.

Introduction

It is the mission of the physician to safeguard the health of the people. His or her knowledge and conscience are dedicated to the fulfillment of this mission.

The Declaration of Geneva of the World Medical Association binds the physician with the words, "The health of my patient will be my first consideration", and the International Code of Medical Ethics declares that, "A physician shall act only in the patient's interest when providing medical care which might have the effect of weakening the physical and mental condition of the patient."

The purpose of biomedical research involving human subjects must be to improve diagnostic, therapeutic and prophylactic procedures and the understanding of the aetiology and pathogenesis of disease.

In current medical practice most diagnostic, therapeutic or prophylactic procedures involve hazards. This applies especially to biomedical research.

Medical progress is based on research which ultimately must rest in part on experimentation involving human subjects.

In the field of biomedical research a fundamental distinction must be recognized between medical research in which the aim is essentially diagnostic or therapeutic for a patient, and medical research, the essential object of which is purely scientific and without implying direct diagnostic or therapeutic value to the person subjected to the research.

Special caution must be exercised in the conduct of research which may affect the environment, and the welfare of animals used for research must be respected.

Because it is essential that the results of laboratory experiments be applied to human beings to further scientific knowledge and to help suffering humanity, the World Medical Association has prepared the following recommendations as a guide to every physician in biomedical research involving human subjects. They should be kept under review in the future. It must be stressed that the standards as drafted are only a guide to physicians all over the world. Physicians are not relieved from criminal, civil, and ethical responsibilities under the laws of their own countries.

I. Basic Principles

1. Biomedical research involving human subjects must conform to generally accepted scientific principles and should be based on adequately performed laboratory and animal experimentation and on a thorough knowledge of the scientific literature.

2. The design and performance of each experimental procedure involving human subjects should be clearly formulated in an experimental protocol which should be transmitted for consideration, comment and guidance to a specially appointed committee independent of the investigator and the sponsor provided that this independent committee is in conformity with the laws and regulations of the country in which the research experiment is performed.

3. Biomedical research involving human subjects should be conducted only by scientifically qualified persons and under the supervision of a clinically competent medical person. The responsibility for the human subject must always rest with a medically qualified person and never rest on the subject of the research, even though the subject has given his or her consent.

4. Biomedical research involving human subjects cannot legitimately be carried out unless the importance of the objective is in proportion to the inherent risk to the subject.

5. Every biomedical research project involving human subjects should be preceded by careful assessment of predictable risks in comparison with foreseeable benefits to the subject or to others. Concern for the interests of the subject must always prevail over the interests of science and society.

6. The right of the research subject to safeguard his or her integrity must always be respected. Every precaution should be taken to respect the privacy of the subject and to minimize the impact of the study on the subject's physical and mental integrity and on the personality of the subject.

7. Physicians should abstain from engaging in research projects involving human subjects unless they are satisfied that the hazards involved are believed to be predictable. Physicians should cease any investigation if the hazards are found to outweigh the potential benefits.

8. In publication of the results of his or her research, the physician is obliged to preserve the accuracy of the results. Reports of experimentation not in accordance with the principles laid down in this Declaration should not be accepted for publication.

9. In any research on human beings, each potential subject must be adequately informed of the aims, methods, anticipated benefits and potential hazards of the study and the discomfort it may entail. He or she should be informed that he or she is at liberty to abstain from participation in the study and that he or she is free to withdraw his or her consent to participation at any time. The physician should then obtain the subject's freely-given informed consent, preferably in writing.

10. When obtaining informed consent for the research project the physician should be particularly cautious if the subject is in a dependent relationship to him or her or may consent under duress. In that case the informed consent should be obtained by a physician who is not engaged in the investigation and who is completely independent of this official relationship.

11. In case of legal incompetence, informed consent should be obtained from the legal guardian in accordance with national legislation. Where physical or mental incapacity makes it impossible to obtain informed consent, or when the subject is a minor, permission from the responsible relative replaces that of the subject in accordance with national legislation.

    Whenever the minor child is in fact able to give a consent, the minor's consent must be obtained in addition to the consent of the minor's legal guardian.

12. The research protocol should always contain a statement of the ethical considerations involved and should indicate that the principles enunciated in the present Declaration are complied with.

II. Medical Research Combined With Professional Care (Clinical Research)

1. In the treatment of the sick person, the physician must be free to use a new diagnostic and therapeutic measure, if in his or her judgment it offers hope of saving life, reestablishing health or alleviating suffering.

2. The potential benefits, hazards, and discomfort of a new method should be weighed against the advantages of the best current diagnostic and therapeutic methods.

3. In any medical study, every patient - including those of a control group, if any - should be assured of the best proven diagnostic and therapeutic methods.

4. The refusal of the patient to participate in a study must never interfere with the physician-patient relationship.

5. If the physician considers it essential not to obtain informed consent, the specific reasons for this proposal should be stated in the experimental protocol for transmission to the independent committee (I, 2).

6. The physician can combine medical research with professional care, the objective being the acquisition of new medical knowledge, only to the extent that medical research is justified by its potential diagnostic or therapeutic value for the patient.

III. Non-Therapeutic Biomedical Research Involving Human Subjects (Non-Clinical Biomedical Research)

1. In the purely scientific application of medical research carried out on a human being, it is the duty of the physician to remain the protector of the life and health of that person on whom biomedical research is being carried out.

2. The subjects should be volunteers - either healthy person or patients for whom the experimental design is not related to the patient's illness.

3. The investigator or the investigating team should discontinue the research if in his/her or their judgment it may, if continued, be harmful to the individual.

4. In research on man, the interest of science and society should never take precedence over considerations related to the well-being of the subject.

5.4 Informed Consent

The Investigator will obtain informed consent from each subject enrolled in the study, in accordance with the Declaration of Helsinki, the current version of the ICH guidelines and the laws and regulations of the country in which the investigation is being conducted.

The Ethics Committee must accept the informed consent document to be used by the Investigator. It is the responsibility of the Investigator to assure that the patient (or guardian or legal representative) has signed the Informed Consent before any activity or treatment is undertaken which is not part of routine care. This includes, but is not limited to, the performance of diagnostic or therapeutic procedures and the administration of the first dose of study medication.

5.5 Elements of Informed Consent

This informed consent must include the following items:
1. A statement that the study involves research.
2. An explanation of the purposes of the research.
3. A statement approximating the number of subjects expected to be involved in the study.
4. A statement regarding the expected duration of the subject’s participation in the study.
5. An explanation of the study treatments and the probability for random assignment to each treatment arm.
6. Explanation of the experimental procedures.
7. The study procedures to be followed, including all invasive procedures.

8. The subject’s responsibilities.
9. A description of the reasonably foreseeable risks or inconveniences to the subject and, when applicable, to an embryo, fetus or nursing infant.
10. A statement that the treatment may involve risks that are currently unforeseeable.
11. A disclosure of anticipated expenses, if any, to the subject for participating in the study.
12. A description of the expected benefits. When there is no intended clinical benefit to the subject, the subject should be made aware of this.
13. A statement regarding the anticipated prorated payment, if any, to the subject for participating in the study.
14. A disclosure of alternative procedure(s) or course(s) of treatment that may be available to the subject, and their important potential benefits and risks.
15. A statement that the subject or the subject’s legally acceptable representative will be informed in a timely manner if information becomes available that may be relevant to the subject’s willingness to continue participation in the study.
16. A statement explaining compensation and/or treatment available to the subject in the event of study-related injury.
17. Identification of the person(s) to contact for further information regarding the study.
18. Whom to contact about the rights of study subjects.
19. Whom to contact in event of study-related injury.
20. A statement explaining that people from Immtech International, Inc. or independent companies monitoring the study and auditing the results on behalf of Immtech International, Inc., the researchers including the subject's study doctor, the ethics committee and domestic and foreign regulatory authorities will have access to your original medical records for the purpose of collecting data, verifying that the data is correct and checking that the study is conducted properly.
21. A statement explaining that records identifying the subject will be kept confidential, except to the extent shared according to this authorization, and, to the extent permitted by the applicable laws and/or regulations, will not be made publicly available. If the results of the study are published, the published report will not identify study subjects by name.
22. A statement explaining access to records.
You have the right to access your medical records as allowed by national law.
The Sponsor will take reasonable steps to protect your right to privacy.
This authorization has no expiration date. This is because information that is collected for research purposes continues to be analyzed for many years and it is not possible to determine when the analysis will be complete.
24. A statement explaining the potential uses of the study subject data.
Sample language: The information collected in this study will be processed to meet the purpose of the clinical study. Information may be used for seeking approval from domestic and/or foreign regulatory authorities to market the studied drug. It may also be used in reports of the study or for scientific presentations.
The Sponsor may also use the information from this study which relates to you for future medical research, which may be performed by the Sponsor together with other companies or researchers. Explaining the foreseeable circumstances and/or reasons under which the subject’s participation in the study may be terminated.

26. Procedures for orderly termination of participation.
27. A statement explaining that the subject’s participation is voluntary.
28. A statement that the subject may refuse to participate without penalty or loss of benefits to which the subject is otherwise entitled.
29. A statement explaining revocation of consent and use of pre-revocation data.
Sample language: You may withdraw from the study, at any time without penalty or loss of benefits. Withdrawal from the study does not automatically revoke the authorization to use or disclose personal information. The request to revoke authorization to use or disclose personal information must be received in writing.
The request to revoke authorization does not include information that has already been disclosed or information gathered prior to the revocation as a result of your participation in the study or is needed to preserve the scientific integrity of the study. The study data, which may include personal information, will continue to be used by the Sponsor to the extent that it has been relied upon.
30. A statement that a signed and dated copy of the consent is given to the subject or the subject's legally authorized representative.
31. A statement explaining that, by signing the informed consent form, the subject or the subject’s legally acceptable representative is authorizing such access to the subject’s personal data as described elsewhere in this consent.
32. A statement of agreement to participate, e.g., "I agree to participate..."
33. A place for the subject to sign and date.
34. A place for the subject's legally acceptable representative to sign and date (if applicable).
35. A place for the signature and date of the person who conducted the informed consent discussion.

Appendix 6 Other Administrative and Regulatory Procedures

6.1 Data Quality Assurance

Prior to the initiation of the study, an investigator's meeting will be held with the investigators and their study coordinators and staff from STI. This meeting will include a detailed discussion of the protocol, performance of study procedures, CRF completion, and specimen collection methods. In addition to the investigator's meeting, the study personnel at each site will be trained on the study procedures by STI personnel at a study initiation visit.

STI personnel will monitor each site throughout the study. Source document review will be performed against entries on the CRF and a quality assurance check will be performed to ensure that the investigator is complying with the protocol and regulations. In addition, after CRFs are retrieved by the STI personnel, a review of the data will be conducted by a physician and a clinical review team at Immtech International, Inc.

All data hand entered in the database will be verified by a double-key entry procedure. Any discrepancies will be reviewed against the hard copy CRF and corrected on-line. After completion of the entry process, computer logic checks will be run to check for such items as inconsistent study dates and outlying laboratory values. Any necessary corrections will be made to the database and documented via addenda or audit trail. A manual review of selected line listings will also be performed at the end of the study.

6.2 Clinical Laboratory Tests and Normal Laboratory Values (NLV)

Clinical laboratory tests should be performed by the same laboratory throughout the study. The normal ranges of the laboratory that will perform the tests required by the protocol must be transmitted to Immtech International, Inc. Any change in normal laboratory values during this study will be transmitted to Immtech International, Inc.

6.3 Monitoring

Monitors or monitors designated by Immtech International, Inc. will conduct site visits to the investigational facilities for the purpose of monitoring the study. The Investigator agrees to allow these monitors and other authorized Immtech International, Inc. personnel access to the clinical supplies dispensing and storage area and study documentation for the above-mentioned purpose and agrees to assist the site visitors in their activities, if requested. Requests by regulatory agencies to inspect study sites could possibly be made after notification. The Investigator agrees to allow inspectors from regulatory agencies to review records and is encouraged to assist the inspectors in their duties, if requested.

6.4 Auditing

Independent auditors designated by Immtech International, Inc. will conduct a systematic examination of study related activities, documents and selected sites to assess whether the evaluated study activities were conducted, and data were recorded, analyzed and accurately reported according to approved protocol, standard operating procedures current Good Clinical Practice, and the applicable regulatory requirements. Audit observations and findings will be documented and communicated to appropriate study personnel and management. A corrective and preventative action plan will be requested and documented in response to any audit observations. Audit reports and responses to audit observations must be returned to Immtech International, Inc. When required by regulations, Immtech International, Inc. will provide an audit certificate.

6.5 Source Documents and Case Report Forms (CRFs)

Subject source documents are the physician's subject records maintained at the study site. In most cases, the source documents will be the hospital's or the physician's chart. In cases where the source documents are the hospital's or the physician's chart, the information collected on the CRFs must match those charts. In some cases, a portion of the source documents for a given subject may be the CRFs.

The CRFs will be printed on NCR ("no carbon required") paper to permit multiple copies. The bottom copy is to be retained at the site for the Investigator's study file. All questions should be answered using a black ink ballpoint pen. If certain data are not available, not done, or not applicable: "NAV," "ND," or "NAP," respectively, will be entered in the appropriate space.

Twenty-four hour clock should be used for all time entries. Changes and/or additions to data entered on original CRFs must be made in the following manner: The original entry will be lined out with a single line drawn through the error (not erased or "whited out") so as to leave it still legible. The correction will be entered using a black ink ballpoint pen, initialed, and dated by the person making the correction. The Investigator or delegate (e.g., Sub-Investigator or study coordinator), may enter corrections on original CRFs. The monitoring team may make changes to the copies of CRFs based on information supplied by the Investigator and documented in the study file.

The procedure for submitting the CRFs to Immtech International, Inc. will be described to the study site personnel by the Monitor or delegate.

Periodically, where appropriate, the Monitor or other authorized Immtech International, Inc. personnel will visit the study site for the purpose of comparing the data on the CRFs with the source documents. The Investigator agrees to make source documents available for this purpose.

The CRFs should be completed as soon as possible after the data are available. They should be collected by the Monitor at the next site visit.

6.6 Disposition of Clinical Supplies

The Investigator will maintain adequate records showing the receipt, dispensing, return, or other disposition of the investigational drug, including the date, quantity, batch or code number, and identification of subjects (number, initials) who received study drug.

When the investigation is discontinued or completed, unused supplies of drug will be returned or disposed of, as directed by the Monitor or monitors designated by Immtech International, Inc.

Under no circumstances will the Investigator supply clinical supplies to other Investigators or clinics, or allow the supplies to be used other than as directed by this protocol without prior authorization from Immtech International, Inc.

6.7 Maintenance of Records

The Investigator will retain a copy of all study documents, including reports to the Ethics Committee and to Immtech International, Inc., in accordance with the FDA or local regulations, whichever are the more stringent.

The Investigator will maintain all study documents:

a. for a minimum of two years following the date the last marketing application (U.S. NDA/BLA or equivalent) is approved for the drug that was under clinical investigation

OR

b. for a minimum of two years following the release date of the final report, if no marketing application (U.S. NDA/BLA or equivalent) is to be filed by Immtech International, Inc., or if the marketing application (U.S. NDA/BLA or equivalent) is not approved for the indication for which the drug was under clinical investigation or is discontinued and the FDA has been notified.

OR

c. for any longer period that is specified by the regulatory requirements of the country in which the study site is located

If the Investigator relocates, retires, or for any reason withdraws from the study, the study records may be transferred to an acceptable designee, such as another Investigator, another institution, or to Immtech International, Inc. The Investigator must obtain Immtech International's written permission before disposing of any records.

It is the responsibility of the sponsor to inform the investigator/institution as to when these documents no longer need to be retained.

6.8 Final Report

Upon request by the Investigator, at the completion of the study and following analysis of the data, Immtech International, Inc. will supply a listing of the Investigator's subjects' treatment assignments, tabulated data, and statistical analyses, as appropriate. A copy of the final study report and corrected CRFs and/or change requisitions including a receipt to be signed and returned to Immtech International, Inc., will be provided to each Investigator following its release by Immtech International, Inc.

6.9 Changes in Protocol

Changes to the protocol (after Signatures of Agreement are obtained) that affect the decision of the Ethics Committee (e.g., more extensive procedures, increased risk to subjects, changes in the subject population, additional safety information, etc.) must be documented in the form of an amendment. This amendment must be signed by the appropriate Immtech International, Inc. personnel and the Investigator, and approved by the Ethics Committee before it may be implemented. If the amendment is minor or reduces the risk to the subject, the chairperson of the Ethics Committee alone may approve it. Ethics Committee acceptance is not necessary for protocol clarifications that consist of minor protocol changes such as correcting typographical errors, rewording for clarity, changes in monitoring personnel, or for other changes to the protocol that do not affect the conduct of the study, including changes in the plan for statistical analysis.

The only circumstances in which the amendment may be initiated without Ethics Committee acceptance is where the change is necessary to eliminate apparent immediate hazards to the subjects. In that event, the Investigator must notify the Ethics Committee in writing within five (5) working days after the implementation.

6.10 Use of Information

All information concerning DB289 or other Sponsor confidential information, including patent applications, formulas, manufacturing processes, basic scientific data, or and not previously published is considered confidential information.

The information developed during the conduct of this clinical study is also considered confidential and will be used by Immtech International, Inc. in connection with the development of DB289. This information may be disclosed as deemed necessary by Immtech International, Inc. to other clinical investigators, other pharmaceutical companies, to the US FDA and to other governmental agencies. To allow for the use of the information derived from this clinical study and to ensure complete and thorough analysis, the investigator is obligated to provide Immtech International, Inc. with complete test results and all data developed in this study and to provide direct access to source data/documents for trial-related monitoring, audits, IEC/IRB review, and regulatory inspection.

This confidential information shall remain the sole property of Immtech International, Inc., shall not be disclosed to others with out the written consent of Immtech International, Inc., and shall not be used except in the performance of this study.

Appendix 7 Overdose Instructions

No specific studies have been performed regarding the management of overdose with DB289. Administration of drug should be stopped and supportive measures and symptomatic treatment instituted.

Appendix 8 Instructions for the Collection, Handling, and Shipping of Samples

8.1 Plasma Samples for Pharmacokinetic Analysis

Samples may be affected by heat and must be handled and stored under appropriate conditions.

Maximum of 1 hour at room temperature, 4 hours at 4°C (refrigerator); storage at minimum –20°C.

Each sample (tube) is immediately labeled before processing; all tubes used during processing of the samples are labeled with the correct sample number!

The time points of sampling are described in the CRF and a special flowchart posted in the laboratory of the center.

Sample handling

- Collect blood (4-5 mL) by venipuncture into a 5 mL potassium EDTA Vacutainer tube. Invert the tube gently 10 times to mix the anticoagulant. Within 30 minutes of collection, the blood sample should be centrifuged at 2000 rpm for 10 minutes.

- The supernatant is aliquoted into 1.8 mL cryotubes (provide two (2) aliquots of 1.0 ml of each sample). The tubes are labeled "Trial patient #; Px (n/N), Date and exact time of collection (# is the patient number in the trial, Px is the sample number according to the protocol, N is the total number of aliquots stored of this sample and n the number of the aliquot). The number of tubes per drawn sample has to be indicated in the respective form of the CRF.

- All samples are frozen horizontally at –20°C, and shipped in liquid nitrogen dry shippers vertically if possible in order to detect thawed or partially thawed samples. All exceptions (i.e. power failure and direct storage in N_2) from this rule must be documented in form "observations" of the CRF. All samples should be frozen within 1 hour from blood draw.

8.2 Breast Milk Samples for Pharmacokinetic Analysis

- 10 ml of breast milk are collected or with a breast milk pump. The milk will be aliquoted into two 5 ml cryotubes tubes and immediately frozen at -20°C.

  (For detailed instructions for handling, labeling, freezing and storage conditions see 8.1, above).

8.3 Serum Samples for Clinical Chemistry Analysis

– The time points of sampling are described in the CRF and a special flowchart posted in the laboratory of the center.

– Serum is obtained by collection of blood into vacutainer tubes with coagulation activator. The tubes are gently inverted 10 times to mix the anticoagulant, allowed to stand for 10 min and centrifuged at 2000 RPM for 10 min.

– The supernatant is pipetted into a fresh tube, stored at 4°C and used for clinical chemistry immediately.

8.4 Blood Samples for PCR Genotyping of Trypanosomes.

Full blood will be collected by venipuncture in a 7ml vacutainer tube containing heparin, and will be centrifuged according to 8.1(above). The plasma (supernatant) is carefully discarded and the complete pellet (blood cells) including the buffy coat transferred into a 5 ml Cryotube containing 2 ml of lysis buffer (provided). The tube is closed, mixed well and kept in the refrigerator (preferably) or at room temperature.

8.5 Rules for Handling of Liquid Nitrogen (N_2)

- It is strictly forbidden for uninstructed personnel to handle liquid N_2, or containers

- Liquid nitrogen (N_2) is very harmful upon contact with skin or body parts !!!

- Gloves and protective glasses have to be used for all manipulations. Feet have to be covered by closed shoes (no rubber boots). Sample racks may only be touched with forceps.

- To check the N_2 level in liquid container, use the provided plastic ruler exclusively!

- Immediately close storage containers after use!

8.6 Samples Shipment Frozen in Liquid Nitrogen

The samples are preferably shipped in a liquid nitrogen shipping container (e.g. Chart EC VSS, CryoPort) to the analyzing laboratory. Alternatively a large Styrofoam box containing an adequate amount of dry ice may be used if transport is direct and guaranteed to last less than 24 hours. The transport is organized by the contact organization in the country with support and according to the directions of the STI.

A specimen inventory sheet needs to be included in each shipment. The specimen inventory sheet should include the following information:

- Subject Identifier such as ID#
- Collection time point

- Individual specimen identifier (if assigned)
- Total number of samples shipped
- Any discrepancies the shipper is aware of at the time of shipment

Before shipment the labels of the specimens checked for clarity, readability and correctness to allow cross-referencing to the specimen inventory sheet. The shipping documentation must be prepared in collaboration with the shipping agency. All relevant documents, including the applicable dangerous goods declarations must be prepared.

Specimens should be placed in an appropriate container to prevent breakage and to maintain organization. Examples of containers include cardboard or Styrofoam sample storage boxes with dividers. To meet Federal guidelines for the transportation of biological materials, specimen containers must be placed in primary and secondary leak proof containers. This can be accomplished by placing the specimen containers in two leak proof plastic bags.

The specimen inventory list and other documentation will be included in the container.

All specimens will be shipped to the following address:

Tandem Labs
1121 East 3900 South
Salt Lake City, UT 84124
USA

The outside of the shipping container should be labeled as follows:

PERISHABLE DIAGNOSTIC SAMPLES
TO BE FROZEN UPON RECEIPT
ORM-A

List the biological hazards status for the specimens on the outside of the container, if applicable.

Tandem Labs (formerly Northwest Bioanalytical) will be notified by the clinical site prior to shipment of samples by phone or fax. The clinical site will identify the number of packages, carrier, ship date and anticipated delivery date.

Please direct all information and inquiries regarding shipment receipt and analysis to Shaundel Percey, Project Manager, at 1-801-313-6448, Fax 1-801-293-2389.

Appendix 9 Publication Policy

The results of the trial will be reported to and discussed with Immtech International Inc. and the University of North Carolina, Chapel Hill, and subsequently made public by scientific publications. A publication policy observing the standards for authorship will be employed[63]. Any author must have made significant contributions to (a) the conception and design, or analysis and interpretation of data; and to (b) drafting the article or revising it critically for important intellectual content; and on (c) final approval of the version to be published. Each author must have participated sufficiently in the work to take public responsibility for the total content of a publication. Authorship will be determined by the sponsor, in discussion with the principle investigator and study director, and based on the contributions to the study. The publications generated as a result of the study will conform to the recommendations of the CONSORT statement[64].

Appendix 10 Essential Documents

Prior to the beginning of any clinical study, the investigator will be asked to provide the following documents to STI, the CRO, who will be responsible for transferring them to the Sponsor:

1. A signed and dated protocol for the study.

2. A signed and dated Form FDA 1572, or local derivative form, with Investigator information, address and specialty, certifying the investigator's agreement to comply with the appropriate (*e.g.*, United States 21 CFR) regulations governing the conduct of the study.

3. A current *curriculum vita* of the investigator. If sub-investigators will participate in the study, curriculum *vitae* for each are to be provided.

4. Requirements for the Independent Ethics Committee (IEC)/Institutional Review Board (IRB).

   – A copy of the signed and dated letter of approval of the IEC/IRB. The letter must specify that both the protocol and informed consent form were approved (unless separate documentation that the informed consent was approved is provided).

   – A dated list containing the names and affiliations of the members of the IEC/IRB, or the institution's General Assurance Number.

   – If the investigator and/or sub-investigator is a member of the IEC/IRB, a letter stating that he/she did not participate in the review or approval of the protocol or informed consent form.

5. A specimen copy of the IEC/IRB-approved informed consent document to be used in the study.

6. A list of reference ranges of values for all laboratory tests specified by the protocol.

Appendix 11 Efficacy and Safety Measurements Assessed and Flow Chart

Visits	SC[A]	BS[B]	Treatment period (days)												Follow up (months)				
Days	-48	-7 to -1	1	2	3	4	5	6	7	8	9	10	11	13					
Months															3	6	12	18	24
Diagnostic Procedures (Parasites in blood and lymph)	X	X							X[C]				X[C]		X	X	X	X	
Lumbar Puncture (Parasites and WBC in CSF, IgM Latex)		X														X	X	X	(X)
Informed Consent		X																	
Demographics		X																	
Medical History		X																	
HAT Signs and Symptoms		X							X[D]				X[D]		X	X	X	X	X
Vital Signs		X	X	X	X	X	X	X	X	X[E]	X[E]	X[E]	X[E]						
Inclusion / Exclusion Criteria		X																	
Physical Exam, Coma Scale		X							X[D]				X[D]						X
Laboratory Testing[F]		X							X[G]		(X[G])		X[G]	(X[G])	X[Q]				
ECG[F]		X	X						X[H]				X[H]						
Pregnancy Testing (urine)		X							X[D]				X[D]						
Evaluation of Infants of Pregnant & Lactating Women		X							X[C,J]				X[C,J]		X[J]	X[J]	X[J]	X[J]	X[J]
PCR Genotyping		X							X[K]				X[K]		X[K]	X[K]	X[K]	X[K]	X[K]
DB289 Oral Administration BID			X[L]	X[L]	X[L]	X[L]	X[L]	X[L]	X[L]	X[L]	X[L]	X[L]	X[L]						

**Visits**	**SC**[A]	**BS**[B]	**Treatment period (days)**												**Follow up (months)**				
**Days**	**-48**	**-7 to -1**	**1**	**2**	**3**	**4**	**5**	**6**	**7**	**8**	**9**	**10**	**11**	**13**					
**Months**															**3**	**6**	**12**	**18**	**24**
Pentamidine i.m. Injection QD			X[M]	X[M]	X[M]	X[M]	X[M]	X[M]	X[M]										
Treatment Emergent Adverse Events			X	X	X	X	X	X	X	X	X	X	X	X	X[N]	X[N]	X[N]	X[N]	X[N]
Concomitant Medications		X	X	X	X	X	X	X	X	X[O]	X[O]	X[O]	X[O]						
Pharmacokinetic Samples for Pregnant & Lactating Women								X[P]	X[P]	X[P]	X[P]	X[P]	X[P]						

[A]Screening performed within 6 weeks of baseline evaluation.
[B]Baseline performed within one week prior to first dosing
[C]Day 7 for Pentamidine, Day 11 for DB289
[D]Baseline & Day 7 for Pentamidine; Baseline & Day 11 for DB289
[E]Baseline & daily evaluation for 7 days for Pentamidine; Baseline & daily evaluation for 11 days for DB289
[F]Selected sites only
[G]Baseline & Day 7 for Pentamidine; Baseline & Day 11 for DB289; In case of laboratory values = Grade 2, repeat two days thereafter
[H]Baseline & Day 7 for Pentamidine; Baseline & Day 7 & Day 11 for DB289
[J]Delivery and early childhood development history
[K]In case of parasitological treatment failure or relapse
[L]Twice a day for DB289 for 11 days (20 doses), starting in the evening of Day 1, last dose morning of Day 11
[M]Once a day for Pentamidine for 7 days (7 doses), starting in the morning of Day 1, last dose morning of Day 7
[N]Collect and report all serious adverse events (see Appendix 4)
[O]DB289 subjects only.
[P]DB289 subjects only. Any day (Day 6 – 11), 1 sample each: pre-dose & 4-6 hours after drug application (maximum drug level); Day 11 – 1 sample 24 hours after last dose
[Q]Subjects ages 12 – 15 only.

Appendix 12 Grading of Clinical Signs and Symptoms of HAT[59]

	Grade 0	Grade 1	Grade 2
Lymphadenopathy (cervical posterior)	absent	palpable (> 1 cm)	---
Fever	absent	> 37.5ºC	
Headache	absent	present	unbearable
Pruritus	absent	present	visible traces of scratching
Daytime sleep	normal	repeatedly	continuously
Nighttime sleep	normal	few hours	rare
Tremor	absent	visible	severe
Speech impairment	absent	present	uninterpretable speech
Abnormal movements	absent	present	inability to perform daily tasks
Walking disability	absent	walking with difficulties	walking with help or inability to walk
General motor weakness	absent	ability to stand up from chair without use of hands	no ability to stand up from chair without use of hands
Unusual behavior	absent	present	severe
Inactivity	absent	reduced workforce	inability to perform daily tasks
Aggressive behavior	absent	sporadic	severe, requires observation
Appetite	normal	disturbed	severely disturbed

REFERENCES

[1] Greenwood BM, Whittle HC. The pathogenesis of sleeping sickness. Transactions of the Royal Society of Tropical Medicine and Hygiene 1980; 74:716-724.

[2] WHO. Control and surveillance of African trypanosomiasis. 1998.

[3] Miezan TW, Meda HA, Doua F, Yapo FB, Baltz T. Assessment of central nervous system involvement in gambiense trypanosomiasis: value of the cerebro-spinal white cell count. Tropical Medicine and International Health 1998; 3:571-5.

[4] Doua F, Miezan TW, Sanon Singaro JR, Boa Yapo F, Baltz T. The efficacy of pentamidine in the treatment of early-late stage Trypanosoma brucei gambiense trypanosomiasis. American Journal of Tropical Medicine and Hygiene 1996; 55:586-8.

[5] Anonymous editor. Preliminary results of a clinical trial comparing melarsoprol to pentamidine for the treatment of early stage two *T. b. gambiense* patients in Uganda. 1998.

[6] Nightingale S. Drug for sleeping sickness approved. Journal of the American Medical Association 1991; 265:1229.

[7] Gustafsson LL, Beerman B, Aden Abdi Y. Handbook of drugs for tropical parasitic infections. 1. ed. Basingstoke: Taylor & Francis, 1987.

[8] Sands M, Kron MA, Brown RB. Pentamidine, a review. Reviews of Infectious Diseases 1985; 7:625-634.

[9] Pepin J, Donelson JE. African Trypanosomiasis (Sleeping Sickness). In: Guerrant RL, Walker DH, Weller PF, editors. Tropical Infectious Diseases; Principles, Pathogens & Practice. 1 ed. Philadelphia: Churchill Livistone, 1999:774-784.

[10] Doua F, Yapo FB. Human trypanosomiasis in the Ivory Coast - therapy and problems. Acta Tropica 1993; 54:163-168.

[11] Stauffert I, Paulini H, Steinmann U, Sippel H, Estler CJ. Investigations on mutagenicity and genotoxicity of pentamidine and some related trypanocidal diamidines. Mutation Research 1990; 245:93-8.

[12] Conte JE. Pharmacokinetics of intravenous pentamidine in patients with normal renal function or receiving haemodialysis. Journal of Infectious Diseases 1991; 163: 169-175.

[13] Bronner U, Gustafsson LL, Doua F, Ericsson O, Miezan T, Rais M, et al. Pharmacokinetics and adverse reactions after a single dose of pentamidine in patients with Trypanosoma gambiense sleeping sickness. British Journal of Clinical Pharmacology 1995; 39: 289-95.

[14] Bronner U, Doua F, Ericsson O, Gustafsson LL, Miezan TW, Rais M, et al. Pentamidine concentration in plasma, whole blood and cerebrospinal fluid during treatment of *Trypanosoma gambiense* infection in Cote d'Ivoire. Trans. Royal Soc. Trop. Med. 1991; 85:608-611.

[15] Glaumann H, Bronner U, Ericsson Ö, Gustafsson LL, Rombo L. Pentamidine accumulates in rat liver lysosomes and inhibits phospholipid degradation. Pharmacology & Toxicology 1994; 74:17-22.

[16] Waalkes TP, Denham C, DeVita VT. Pentamidine: clinical pharmacologic correlations in man and mice. Clinical Pharmacology and Therapeutics 1970; 11:505-12.

[17] Bronner U. Pharmacokinetics of pentamidine. Focus on treatment of *Trypanosoma gambiense* sleeping sickness. PhD Thesis, Karolinska Institute, Stockholm 1994.

[18] Berger BJ, Lombardy RJ, Marbury GD, Bell CA, Dykstra CC, Hall JE, et al. Metabolic N-hydroxylation of pentamidine *in vitro*. Antimicrobial Agents and Chemotherapy 1990; 34:1678-84.

[19] Berger BJ, Naiman NA, Hall JE, Peggins J, Brewer TG, Tidwell RR. Primary and secondary metabolism of pentamidine by rats. Antimicrobial Agents and Chemotherapy 1992; 36:1825-1831.

[20] Berger BJ, Reddy VV, Le ST, Lombardy RJ, Hall JE, Tidwell RR. Hydroxylation of pentamidine by rat liver microsomes. Journal of Pharmacology and Experimental Therapy 1991; 256:883-889.

[21] Wang CC. Molecular mechanisms and therapeutic approaches to the treatment of African trypanosomiasis. Annual Review of Pharmacology and Toxicology 1995; 35:93-127.

[22] Bitonti AJ, Dumont JA, McCann PP. Characterization of *Trypanosoma brucei* brucei S-adenosyl-L-methionine decarboxylase and its inhibition by Berenil, pentamidine and methylglyoxal bis(guanylhydrazone). Biochemical Journal 1986; 237:685-9.

[23] Berger BJ, Carter NS, Fairlamb AH. Polyamine and pentamidine metabolism in African trypanosomes. Acta Tropica 1993; 54:215-224.

[24] Benamin G, Lopez-Estrano C, Docampo R, Moreno SNJ. A calmodulin-stimulated Ca2+ pump in plasma-membrane vesicles from Trypanosoma brucei; selective inhibition by pentamidine. Biochemical Journal 1993; 296:756-763.

[25] Damper D, Patton CL. Pentamidine transport and sensitivity in brucei-group trypanosomes. Journal of Protozoology 1976; 23:349-356.

[26] Frommel TO, Balber AE. Flow cytofluorimetric analysis of drug accumulation by multidrug- resistant *Trypanosoma brucei* brucei and *T. b. rhodesiense*. Molecular and Biochemical Parasitology 1987; 26:183-191.

[27] Jonchère H. Traitement par les diamidines de la phase lymphatico-sanguine de la trypanosomiase humaine en Afrique Occidentale Française. Bulletin de la Société de Pathologie Exotique et ses Filiales 1951; 44:603-625.

[28] Dutertre J, Labusquiere R. La therapeutique de la trypanosomiase. Medecine Tropicale 1966; 26:342-356.

[29] Neujean G, Evens F. Diagnostic et traitement de la maladie de sommeil à *T. gambiense*. Bilan de dix ans d'activité au centre de traitement de Léopoldville. Académie Royale des Sciences Colonials: Classes des Sciences Naturelles et Médicales; Mémoires in 8° 1958; Tome VII.

[30] Bell CA, Hall JE, Kyle DE, Grogl M, Ohemeng KA, Allen MA, et al. Structure-activity relationships of analogs of pentamidine against Plasmodium falciparum and Leishmania mexicana amazonensis. Antimicrobial Agents and Chemotherapy 1990; 34:1381-1386.

[31] Bell CA, Dykstra CC, Naiman NA, Cory M, Fairley TA, Tidwell RR. Structure activity studies of dicationically substituted bis-benzimidazoles against Giardia lamblia: correlation of antigiardial activity with DNA binding affinity of giardial topoisomerase II inhibition. Antimicrobial Agents and Chemotherapy 1993; 37:(12)2668-2673.

[32] Tidwell RR, Jones SK, Geratz JD, Ohemeng KA, Bell CA, Berger BJ, et al. Development of pentamidine analogues as new agents for the treatment of *Pneumocystis carinii* pneumonia. Annals of the New York Academy of Sciences 1990; 616:421-441.

[33] Blagburn B, Drain KL, Land TM, Kinard RG, Moore PH, Lindsay DS, et al. Comparative efficacy evaluation of dicationic carbazole compounds, nitazoxanide and paromomycin against Cryptosporidium parvum infections in a neonatal mouse model. Antimicrobial Agents and Chemotherapy 1998; 42:2877-2882.

[34] Del Poeta M, Schell WA, Dykstra CC, Jones S, Tidwell RR, Czarny A, et al. Structure in-vitro activity relationship of pentamidine analogues and dication substituted bis-benzimidazoles as new antifungal agents. Antimicrobial Agents and Chemotherapy 1998; 42:2495-2502.

[35] Francesconi I, Wilson WD, Tanious FA, Hall JE, Bender BC, McCurdy DR, et al. 2,4-Diphenyl furan diamidines as novel anti-pneumocystis carinii pneumonia agents. J. Med. Chemistry 1999; 33:2260-2265.

[36] Patrick DA, Boykin DW, Wilson WD, Tanious FA, Spychala J, Bender BC, et al. Anti *Pneumocystis carinii* pneumonia activity of dicationic carbazoles. European Journal of Medicinal Chemistry 1997; 32:781-793.

[37] Patrick DA, Hall JE, Bender BC, McCurdy DR, Wilson WD, Tanious FA, et al. Synthesis and anti-*Pneumocystis carinii* pneumonia activity of novel dicationic dibenzothiophenes and diamidoxime prodrugs. European Journal of Medicinal Chemistry 1999; 34:575-583.

[38] Tidwell RR, Jones S, Naiman NA, Berger LC, Brake WB, Dykstra CC, et al. Activity of cationically substituted bis-benzimidazoles against experimental *Pneumocystis carinii* pneumonia. Antimicrobial Agents and Chemotherapy 1993; 37:1716.

[39] Del Poeta M, Schell WA, Dykstra CC, Jones SK, Tidwell RR, Kumar A, et al. In vitro antifungal activity of a series of dication-substituted carbazoles, furans and benzimidazoles. Antimicrobial Agents and Chemotherapy 1998; 42:2503-2510.

[40] Blagburn BL, Drain KL, Land TM, Hutton Moore P, Kinard RG, Lindsay DS, et al. Dicationic furans inhibit development of *Cryptosporidium parvum* in HSD/ICR suckling Swiss mice. J. Parasitology 1998; 84:851-856.

[41] Boykin DW, Spychala J, Zhou M, Lombardy RJ, Wilson WD, Dykstra CC, et al. Dicationic diaryl furans as anti-*pneumocystis carinii* agents. J. Med. Chemistry 1995; 38: 912-916.

[42] Steck E, Kinnanon KE, Davidson DE, Duxbury RE, Johnson AJ, Masters RE. *Trypanosoma rhodesiense*: evaluation of the antitrypanosomal action of 2,5-bis(4-guanylphenyl)furan dihydrochloride. Experimental Parasitology 1982; 53:133-144.

[43] Steck E, Kinnanon KE, Rane DS, Hanson WL. *Leishmania donovani*, *Plasmodium berghei*, *Trypanosoma rhodesiense*: Antiprotozoal effects of some amidine types. Experimental Parasitology 1981; 52:404-413.

[44] Das B and Boykin, DW, Synthesis and antiprotozoal activity of 2,5-bis(4-guanylphenyl)furans. J. Med. Chemistry 1977; 82:(B)531-536.

[45] Bertrand E. Cardiac involvement in human African trypanosomiasis. Med Trop (Mars) 1987; 47:91-3.

[46] Bertrand E, Serie F, Rive J et al. Current aspects of the cardiac symptoms in African human trypanosomiasis due to *Trypanosoma gambiense* (apropos of 194 cases). Acta Cardiol 1974; 29:363-81.

[47] Bertrand E, Sentilhes L, Baudin L, Barabe P, and Aye H. Conduction disorders in African human trypanosomiasis caused by *Trypanosoma gambiense*. Arch Mal Coeur Vaiss 1969; 62:247-53.

[48] Fouchet M and Gateff C. Development of cardiovascular involvement in African trypanosomiasis due to *Trypanosoma gambiense*. Med Trop (Mars) 1968; 28:583-90.

[49] Adams JH, Haller L, Boa FY, Doua F, Dago A, and Konian K. Human African trypanosomiasis (*T.b. gambiense*): a study of 16 fatal cases of sleeping sickness with some observations on acute reactive arsenical encephalopathy. Neuropathol Appl Neurobiol 1986; 12:81-94.

[50] Collomb H and Bartoli D. [The heart in human African trypanosomiasis caused by *Trypanosoma gambiense*]. Bull Soc Pathol Exot Filiales 1967; 60:142-56.

[51] Wang K, Asinger RW, and Marriott HJ. ST-segment elevation in conditions other than acute myocardial infarction. N Engl J Med 2003; 349:2128-35.

[52] Poltera AA, Cox JN, and Owor R. Cardiac valvulitis in human African trypanosomiasis. East Afr Med J 1977; 54:497-9.

[53] Magnus E, Vervoort T, et al, A card agglutination test with stained trypanosomes (CATT) for the serological diagnosis of *T.b. gambiense* trypanosomiasis, Annales de la Société Belge de Médecine Tropicale 1978; 58:169-176.

[54] Woo PT. The haematocrit centrifuge for the detection of trypanosomes in blood. Can J Zool 1969; 47:921-3.

[55] Lumsden WGR, Kimber CD, Evans DA, Doigs J. *Trypanosoma brucei:* Miniature anion exchange centrifugation for detection of low parasitemias: Adaptation for field use. Transactions of the Royal Society of Tropical Medicine and Hygiene 1979; 73:313-317.

[56] Stauffert I, Paulini H, Steinmann U, Sippel H and Estler CJ. Investigations on mutagenicity and genotoxicity of pentamidine and some related trypanocidal diamidines. Mutat Res 1990; 245:93-98.

[57] Turner PR and Denny WA. The mutagenic properties of DNA minor-groove binding ligands. Mutat Res 1996; 355(1-2):141-69.

[58] Connor T H and Trizna T. Pentamidine isethionate is negative in tests for microbial mutagenicity and chromosomal breakage *in vitro*. Toxicol Lett 1992; 63(1):69-74.

[59] Burri C, Nkunku S, Merolle A, Smith T, Blum J, Brun R. Efficacy of new, concise schedule for melarsoprol in treatment of sleeping sickness caused by *Trypanosoma brucei gambiense*: a randomised trial. The Lancet 2000;355:1419-1425.

[60] Miézan TW, Meda HA, et al. Single centrifugation of cerebrospinal fluid in a sealed pasteur pipette for simple, rapid and sensitive detection of trypanosomes. Trans R Soc Trop Med Hyg 2000; 94:293.

[61] Lejon V, Buscher P, Sema NH, Magnus E, Van MN. Human African trypanosomiasis: A latex agglutination field test for quantifying IgM in cerebrospinal fluid. Bulletin of the World Health Organization 1998; 76:553-558.

[62] Ruiz JA, Simarro PP, Josenando T. Control of human African trypanosomiasis in the Quicama focus, Angola. Bulletin of the World Health Organization 2002;80:738-745.

[63] Horton R, Smith R. Signing up for authorship. Lancet 1996; 347:780.

[64] Moher D, Schulz KF, Altman D for the CONSORT group. The CONSORT statement: revised recommendations for improving the quality of reports of parallel-group randomized trials. JAMA 2001; 285:1987-1991.
